# Care groups in an integrated nutrition education intervention improved infant growth among South Sudanese refugees in Uganda’s West Nile post-emergency settlements: A cluster randomized trial

**DOI:** 10.1371/journal.pone.0300334

**Published:** 2024-03-15

**Authors:** Joel J. Komakech, Sam R. Emerson, Ki L. Cole, Christine N. Walters, Hasina Rakotomanana, Margaret K. Kabahenda, Deana A. Hildebrand, Barbara J. Stoecker

**Affiliations:** 1 Department of Food Science, Nutrition, and Health Promotion, Mississippi State University, Starkville, Mississippi, United States of America; 2 Department of Nutritional Sciences, Oklahoma State University, Stillwater, OK, United States of America; 3 Research, Evaluation, Measurement, and Statistics Department, Oklahoma State University, Stillwater, OK, United States of America; 4 Department of Food Technology and Nutrition, Makerere University, Kampala, Uganda; Medical Research Council, SOUTH AFRICA

## Abstract

**Objective:**

This study examined the effects of a peer-led integrated nutrition education intervention with maternal social support using Care Groups on infant growth among South Sudanese refugees in Uganda.

**Methods:**

A community-based cluster-randomized trial (RCT) was conducted among 390 pregnant women (third trimester). Two intervention study arms were Mothers-only(n = 131) and Parents-combined (n = 142) with a Control (n = 117). WHO infant growth standards defined length-for-age z-scores (LAZ) for stunting, weight-for-age z-scores (WAZ) for underweight and weight-for-length z-scores (WLZ) for wasting. The Medical Outcomes Study (MOS) social support index was a proxy measure for social support. A split-plot ANOVA tested the interaction effects of social support, intervention, and time on infant growth after adjusting for covariates. Further, pairwise comparisons explained mean differences in infant growth among the study arms.

**Results:**

The mean infant birth weight was 3.1 ± 0.5 kg. Over the study period, infant stunting was most prevalent in the Control (≥ 14%) compared to Mothers-only (< 9.5%) and Parents-combined (< 7.4%) arms. There were significant interaction effects of the Care Group intervention and social support by time on infant mean LAZ (F _(6, 560)_ = 28.91, *p* < 0.001), WAZ (F _(5.8, 539.4)_ = 12.70, *p* = < 0.001) and WLZ (F _(5.3, 492.5)_ = 3.38, *p* = 0.004). Simple main effects by the end of the study showed that the intervention improved infant mean LAZ (Mothers-only vs. Control (mean difference, *MD)* = 2.05, *p* < 0.001; Parents-combined vs. Control, *MD* = 2.00, *p* < 0.001) and WAZ (Mothers-only vs. Control, *MD* = 1.27, *p* < 0.001; Parents-combined vs. Control, *MD* = 1.28, *p* < 0.001).

**Conclusion:**

Maternal social support with an integrated nutrition education intervention significantly improved infant stunting and underweight. Nutrition-sensitive approaches focused on reducing child undernutrition among post-emergency refugees may benefit from using Care Groups in programs.

**Trial registration:**

Clinicaltrials.gov, NCT05584969.

## Introduction

In 2020 the United Nations (UN) reported that of the 149.2 million stunted children aged 6–59 months the world over, the majority were in Africa and South Asia. However, Africa was the only continent where child stunting levels increased amidst reductions in other regions over the past two decades [1]. Undernutrition is the leading health risk faced by children in low-and middle-income countries (LMICs) [2, 3]. LMICs host the highest proportion of refugees, with the majority comprised of women and children [4]. Displaced children are more vulnerable to undernutrition and poor health [5–9]. Humanitarian agencies have emphasized child undernutrition as one of the key areas for nutrition intervention during emergencies and post-emergency refugee situations [10]. Yet, a review of public health interventions in humanitarian contexts [11] indicated a limited focus on reducing growth failure among infants aged 0–12 months.

Among the refugees in Uganda, the overall prevalence of stunting in 2020 was high at 27.4 percent [12]. Child stunting levels in the West Nile region were at 12.9 percent, acceptable within humanitarian contexts [2]; however, the lack of a stable decline in child stunting trends over the past decade emphasized the need for sustainable interventions to consistently reduce child undernutrition [13]. Poor nutritional status during early childhood limits child growth and development, as well as their potential to thrive later in life due to irreversible impairments [14–17]. However, nutrition-sensitive interventions addressing the underlying causes of infant undernutrition provided through peer support of child caretakers can potentially reduce infant growth failure [18, 19]. When delivered in the first 1000 days window of opportunity, such interventions targeting behavioral change can have lasting impacts on child morbidity and mortality [13, 20–22].

Studies reviewing nutrition-sensitive interventions integrated with education and complementary feeding showed significant associations in reducing child stunting and underweight [23–26]. Furthermore, involvement of fathers and other adults within households in integrated social behavior communication change (SBCC) health interventions improved child care and nutrition [27–31]. However, there is limited information about the mechanisms of behavioral change in nutrition education interventions that impact child growth [32]. Likewise, health and nutrition-related interventions among refugee settlements have increased in the past three decades [33–35], but evidence on the effectiveness of these interventions for improving infant growth in refugee contexts remains limited [11, 36]. Therefore, this study aimed to determine the effects of a peer-led integrated nutrition education intervention delivered through the Care Group model on infant growth in the post-emergency refugee settlements in the West Nile region in Uganda. The findings from this study can be used to scale up peer-led integrated nutrition-sensitive interventions and inform policies on strategies to prevent undernutrition in refugee communities.

## Materials and methods

### Study design and setting

A community-based cluster-randomized trial was conducted between January and December 2020, among South Sudanese refugees in post-emergency settlements in the West Nile region of Uganda. A multi-stage cluster randomized study design [37] that adhered to the CONSORT guidelines [38] for cluster-randomized trials was used to select the Adjumani district among the ten districts hosting refugees in the West Nile region in Uganda. Further, four settlements (Ayilo-I, Ayilo-II, Pagirinya, and Nyumanzi) of the 19 in Adjumani District were randomly selected and assigned to the three study arms, including two intervention study arms (Mothers-only or Parents-combined), and the Control arm. Ayilo-I settlement was assigned as Mothers-only, Pagirinya, and Ayilo II settlements were the Parents-combined (both Mothers and Fathers) intervention study arms, while Nyumanzi settlement was the Control study arm. Each study arm comprised a total of ten groups. Each of the ten groups had 10–20 participants. The settlements were more than six kilometers apart which mitigated any spillover effects of the intervention.

**Fig 1:**
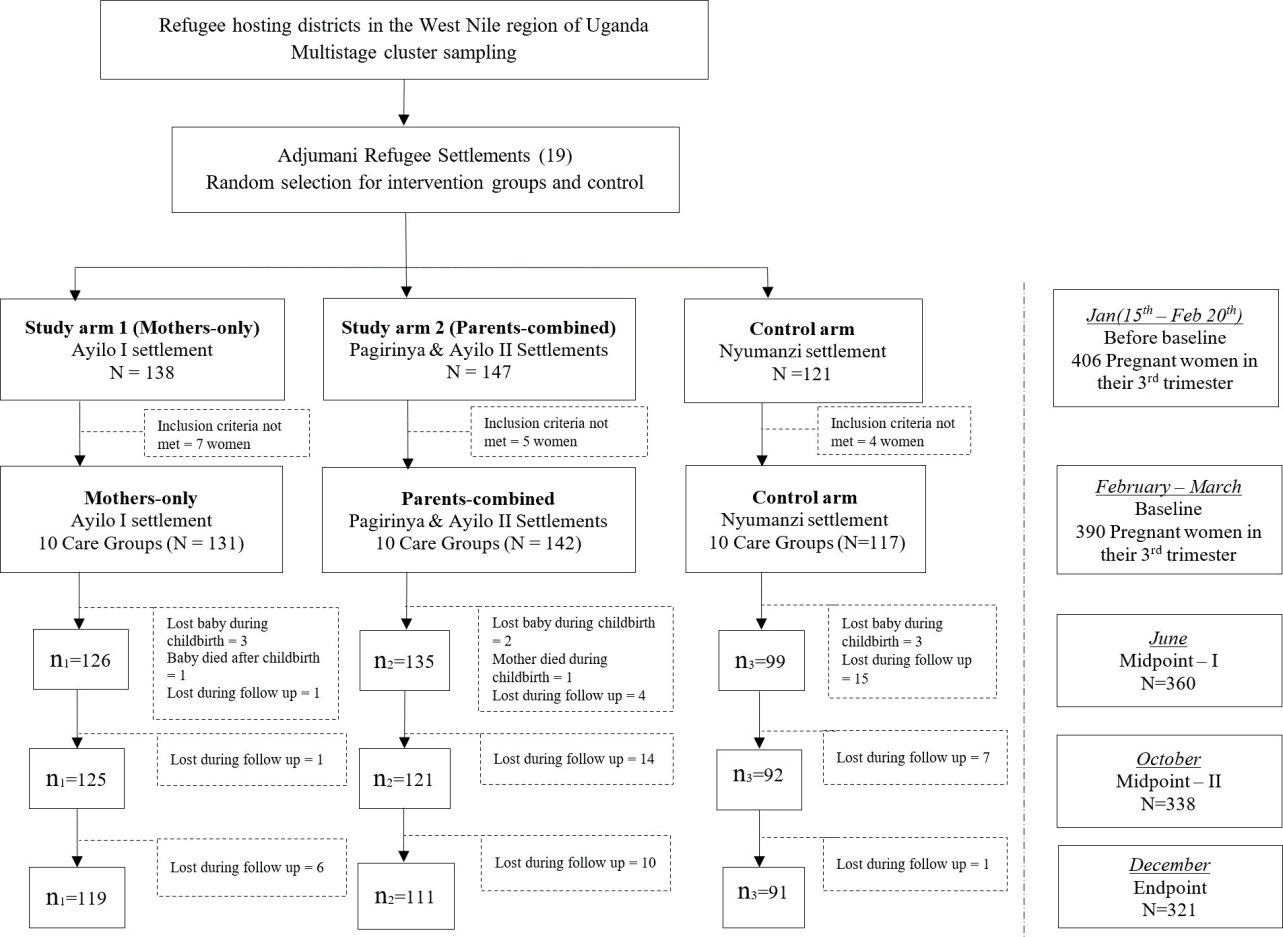
Flow diagram of study design and participants in the study.

A graphical presentation of the study process is shown in the study flow diagram (Fig 1) and the study Gantt chart (S1 Fig). In summary, the identification, screening, and seeking consent for participation began mid-January to February. Initial participant and household data were collected at baseline at the end of February and in early March. The next sets of data including infant characteristics were collected in early June (Midpoint-I), then late September to early October (Midpoint-II), and the final data (Endpoint) were collected in December before the festive season.

### Study participant recruitment

Before the pregnant women were recruited, their settlements were randomly assigned to one of the study arms. Then VHTs and midwife assistants identified the pregnant women using purposive sampling, The pregnant women were enrolled in the study arms with the support of village health team members (VHTs) and assistant midwives, but the midwives and VHTs were kept unaware of the study arm assignment. After the women consented to participate in the study, their pregnancy was confirmed.

In the Parents-combined study arm, husbands together with their wives were required to consent and confirm willingness to participate in the trial. For participation consensus and harmony in the other two study arms, the study and the required length of engagement for the women were explained to spouses. The pregnancy term was verified by crosschecking the pregnant women’s antenatal passports against the record within the Integrated Maternity Register (IMR).

In Uganda, VHTs are volunteers who form the grassroots administrative health system unit in promoting community primary health care, sanitation and hygiene, nutrition best practices, and health-seeking behavior for health center services [39]. VHTs are trained by personnel from the Ministry of Health in Uganda (MoH) or government health partners in health and nutrition education [40]. Midwives and midwife assistants complete a nationally certified standard educational license under MoH, resulting in certification or a diploma in maternal health care practice [41, 42].

### Sample size

The participants included in this study were part of the trial registered at Clinicaltrials.gov as NCT05584969 described and published elsewhere [30]. Briefly, the participants were pregnant women in their 3^rd^ trimester identified before the baseline data collection. The final sample comprised 390 at the start of the trial. A total of 321 mother-infant dyads (82 percent of the original participants) completed the study, with 111 in the Parents-combined study arm, 119 in the Mothers-only study arm, and 91 in the Control arm as shown in the study flow diagram (Fig 1).

### Inclusion and exclusion criteria

Only women with pregnancies categorized as low-risk and with up-to-date maternal records highlighting their third trimester of pregnancy were eligible for inclusion in the study. Specifically, women in their third trimester who had singleton pregnancies in their third trimester and did not engage in smoking or substance abuse, including alcohol were selected.

Mothers whose antenatal records showed pregnancy complications were excluded from the study. Mothers who gave birth to premature infants, infants with congenital abnormalities, or whose infants died could remain in the study; however, their data were excluded from the final analyses.

### The intervention

A peer-led integrated nutrition education intervention was delivered through Care Groups [43] for the two study arms, the Mothers-only arm and the Parents-combined arm. The intervention included peer-led training modules on group dynamics to emphasize group cohesion, infant feeding practices, best childcare practices, child growth indicators, cooking demonstrations, and backyard farming conducted over the ten months. Details of the training modules are indicated in the S5 Table in S2 File.

In the Care Groups, peer leaders were identified and trained based on prepared intervention modules for the Care Group meetings. The district nutrition and health educator, supported by the selected VHTs and the researcher, conducted the training for the Care Group leaders. Refresher training of the Care Group leaders was conducted in the monthly Care Group leaders’ meetings. These Care Group leaders served as volunteer nutrition educators for the regular Care Group meetings and encouraged peer home visits and support of one another in the Care Groups [43, 44]. Over the study period, the Care Group activities were supervised by the VHTs and the researcher. The Care Group meetings lasted 60–90 minutes every two weeks.

### Standard of care

Participants in the Control and the two intervention study arms received regular care and services through the government health system, which comprised the standard of care. The Government of Uganda implements a decentralized referral health system with VHTs as the primary health structure [45]. In this study, each village had access to at least two VHTs serving 50–70 households. Furthermore, participants could go to Health Center III facilities near (within five kilometers) their homes in the refugee settlements [46]. All study participants were expected to have access to health services with training and follow-up through the standard government health system facilities. Further access to referred specialized care to district or regional and higher-level hospitals was also available, yet more expensive due to their location in urban centers. Furthermore, access also depended on the patient’s ability to pay the costs involved, such as transport, and general welfare, even though treatment was free of charge according to government regulations.

### Study measures

Mother-infant dyads were used as the unit of measure to assess the effects of Care Group intervention with maternal social support on infant growth during the study period. A structured questionnaire was used to collect data on the sociodemographic characteristics of mothers, infants, and households during the study. Data collectors/ enumerators were blinded from the study arm assignment. The Medical Outcomes Social (MOS) support scale indicated in Table S4 in S2 File was used as a proxy to measure maternal social support [47]. Higher MOS scores indicated better support. Infant growth was measured using the World Health Organization (WHO) growth standards (WHO, 2008). Stunting, wasting, and underweight were defined as less than -2 z-scores for length-for-age (LAZ), weight-for-length (WLZ), and weight-for-age (WAZ), respectively. Maternal and household characteristics were collected at baseline. The infant’s birth date and weight were recorded from the mother’s responses and crosschecked with the health monitoring card provided at the health center to track the infant’s growth and immunizations. Infant anthropometrics and characteristics were measured initially at Midpoint-I, then at Midpoint-II, and finally at the Endpoint of the study (S1 Fig). All anthropometric measurements were performed in duplicate, and the average was calculated for analyses. Recumbent length was measured to the nearest 0.1 cm using a portable SECA plastic infantometer (model 417) on a flat surface. The mother’s height was measured to the nearest 0.1 cm using a standard wooden UNICEF height board.

Mother and infant had their weights measured simultaneously with light clothing and no shoes using a dual SECA 874 digital scale that automatically calculated individual weights for both mother and child. Weights were recorded to the nearest 0.1 kg.

### Statistical analysis

For this study, a split-plot ANOVA was used with the study effects of one categorical variable (two study arms with the intervention and a Control arm) across three-time points (Midpoint-I, II, and Endpoint) on continuous dependent variables measuring infant growth (LAZ, WAZ, and WLZ). The final dataset for analyses was deidentified for respondent confidentiality. Before completing the split-plot ANOVA, assumptions of normality, equal variances, and sphericity were checked. The Greenhouse-Geisser adjustment was applied to those measures not meeting the assumption of sphericity (p < .05). The split-plot factorial analysis tested the interaction effects of the study arm, social support, and time on infant growth. The split-plot design provided a parsimonious model [48–50] to examine the interaction effects of the intervention over the study period on the growth of infants. The interaction effects models were adjusted for confounders including child age, birthweight, and sex; maternal variables were mother’s height, ethnicity, who supports the mother most, number of living children, and religion; household covariates included number of living children, household wealth index, and years spent in the refugee settlement. Additionally, pairwise comparisons with a Tukey-Kramer correction were conducted to determine the specific mean differences in infant growth following significant interaction effects of the study arm, social support, and time in the omnibus split-plot ANOVA. The Statistical Package for the Social Sciences (SPSS), v. 26 (IBM^©^ SPSS^©^ Statistics, Armonk, NY) was used for analyses with statistical significance set at *p* < 0.05.

### Ethical approval

The Institutional Review Boards of the Uganda National Council of Science and Technology (SS 5038), Makerere University School of Health Science Research and Ethics Committee (SHSREC REF:2019–020), and Oklahoma State University (HS-19-2) approved this study. Additionally, approval from the Office of the Prime Minister (OPM) Uganda (OPM/R/107) was obtained. Participants were given a compensation kit at each data collection period comprising 1 kg bar of washing soap, 200 mL vitamin A fortified cooking oil, and half a kilo each of iodized salt and sugar, worth approximately 7,600 Uganda shillings (1.5 US dollars). All respondents gave verbal informed consent to participate in the study before baseline data collection and during other data collection points over the study period.

## Results

### Maternal sociodemographic characteristics

The characteristics of mothers, households, and infants within each arm are described in Table 1. More than half of mothers in the Parents-combined arm (59.8%) had formal education beyond lower primary compared to less than one-third in the Mothers-only (31.3%) and Control (33.3%) arms. Overall, the Dinka comprised the majority (64.9%) of the ethnic groups. Most mothers in the Control arm (71.8%) and the Mothers-only arm (71.0%) were of the Anglican religion, compared to 24.6 percent in the Parents-combined arm. The mean maternal age was approximately the same across all study arms. The mothers in the Parents-combined arm had the lowest mean height ± SD (164.0 ± 7.3). The average number of living children per household in all study arms was nearly 4.

**Table 1:** Maternal sociodemographic characteristics.

Maternal Variable	Study arms			*p*-value^a^
	Control	Mothers-only	Parents-combined	
	Mean ± SD or % (n)	Mean ± SD or % (n)	Mean ± SD or % (n)	
Education level				<0.001
No formal education	49.6 (58)	45.8 (60)	18.3 (26)	
Lower primary	17.1 (20)	22.9 (30)	21.8 (31)	
Upper primary	26.5 (31)	26.7 (35)	38 (54)	
Secondary and higher	6.8 (8)	4.6 (6)	21.8 (31)	
Marital status^1^				0.371
Married	98.0 (97)	96.8 (122)	99.2 (132)	
Ethnicity				<0.001
Dinka	96.0 (95)	88.8 (111)	16.1 (20)	
Madi	3.0 (3)	11.2 (14)	66.9 (83)	
Other	1.0 (1)	0.0 (0)	16.9 (21)	
Religion				<0.001
Catholic	2.6 (3)	16.8 (22)	50.6 (72)	
Anglican	71.8 (84)	71.0 (93)	24.7 (35)	
Other	25.6 (30)	12.2 (16)	24.7 (35)	
Maternal age, yrs.	27.5 ± 4.9	28.4 ± 5.0	27.3 ± 5.2	0.186
Mother’s Height^2^, cm	169.8 ± 6.2	169.1 ± 7.0	164 ± 7.3	<0.001
Number of living children^┼^	3.9 ± 2.2	3.8 ± 2.1	3.6 ± 2.6	0.447
Place of childbirth^1^				0.002
Home or other	8.1 (8)	0.8 (1)	0.8 (1)	
Local Health Center	70.7 (70)	79.4 (100)	71.2 (94)	
Hospital	21.2 (21)	19.8 (25)	28 (37)	
Type of childbirth^1^				0.662
C-Section	4.0 (4)	5.6 (7)	6.8 (9)	
Vaginal	96.0 (95)	94.4 (119)	93.2 (123)	
Supports the mother most				
Partner/husband	52.3 (46)	40.3 (48)	59.1 (68)	0.051
Peers or Neighbors	11.4 (10)	19.3 (23)	7.8 (9)	
Other relatives	21.6 (19)	30.3 (36)	14.8 (17)	
Other non-relatives	6.8 (6)	4.2 (5)	13.9 (16)	
No one	8.0 (7)	5.9 (7)	4.3 (5)	
Overall social support score^§^^┼^	43.9 ± 7.7	52.7 ± 8.0	61.0 ± 8.8	<0.001
**Household Variables**				
Household head sex				0.004
Female	67.7 (67)	72.2 (91)	53.3 (72)	
Family size	8.59 ± 3.6	8.38 ± 3.1	7.97 ± 3.4	0.316
Socioeconomic status				0.060
Low	41.0 (48)	30.5 (40)	45.8 (65)	
Medium	23.1 (27)	19.8 (26)	18.3 (26)	
High	35.9 (42)	49.6 (65)	35.9 (51)	
Years living in a refugee area^┼^	5.1 ± 1.8	4.8 ± 1.8	4.1 ±.3	<0.001
HFIAS^2^	10.2 ± 5.3	9.7 ± 6.0	8.0 ± 5.2	0.011
**Child Variables**				
Infant/Child Sex^1^				0.007
Male	43.4 (43)	48.4 (61)	62.9 (83)	
Female	56.6 (56)	51.6 (65)	37.1 (49)	
Infant age^1^	3.2 ± 1.2	3.0 ± 1.5	3.2 ± 1.4	0.330
Infant birthweight, kg, SD	3.1 ± 0.4	3.0 ± 0.5	3.2 ± 0.5	0.021

^a^*p*-values were calculated for differences in proportions using chi-square

^1^Variables collected at Midpoint-I after the infant was born

^2^ variables collected at Endpoint; HFIAS- Household Food Insecurity Access Scale

^┼^ Mean scores and standard deviations respectively and study arm means differences performed with ANOVA

^╪^Proportion differences among groups tested using chi-square

^§^Average maternal social support score over the study period calculated using the medical outcomes social support scale; kg–kilogram; mo.–months; SD–Standard Deviation

More than half of the mothers in the Parents-combined and Control study arms reported their spouses as their best source of support compared to the proportion (40.3%) in the Mothers-only study arm (S2a and S2b Tables in S2 File). Mothers in the Parents-combined arm had the highest mean social support score ± SD (61.0 ± 8.8), followed by the Mothers-only arm (52.7 ± 8.0) and the Control arm (43.9 ± 7.7). Maternal social support scores were significantly higher in both arms that received the intervention than in the Control. Further, support in the Parents-combined arm was significantly higher than support in the Mothers-only arm.

Over half of the households were female-headed, and the average family size in all study arms was between eight and nine (Table 1). Almost half in the Mothers-only study arm had a high socioeconomic status compared to about a third in Parents-combined and Control arms. The mean length of stay in the West Nile Region for households in the Parents-combined arm was about four years, less than the other two arms. The highest (worst) mean household food insecurity access scale (HFIAS) score was reported for the Control arm, followed by Mothers-only and Parents-combined arms.

In this study, the highest proportion of male infants (62.9%) was observed in the Parents-combined arm compared to the Mothers-only and the Control arms; the highest proportion of female infants (56.6%) was observed in the Control. The initial overall mean infant age ± SD was 3.2 months ± 1.4 while the mean infant birth weight ± SD was 3.1 kg ± 0.5 among all study arms.

### Infant growth

The infant growth indicators over time are reported in Table 2. The infants in the Control arm had the highest proportion of stunting in all study periods (14.1–20.9%) compared to the Mothers-only and Parents-combined arms (3.4–9.5%). Similarly, infants in the Control arm had the highest proportion (8.1–27.5%) of underweight compared to the intervention study arms (3.7–8.0%) over the study periods. Further, by the end of the study, the infants in the Control arm had the highest proportion of wasting (14.3%) followed by those in the Mothers and Parents combined arms (5.0% and 1.8% respectively).

**Table 2:** Infant growth over the study period.

Infant growth^┼^	Midpoint-I				Midpoint-II				Endpoint			
	Control	Mothers-only	Parents-combined	p-value^a^	Control	Mothers-only	Parents-combined	p-value^a^	Control	Mothers-only	Parents-combined	p-value^a^
	% (n = 99)	% (n = 126)	% (n = 133)		% (n = 92)	% (n = 125)	% (n = 121)		% (n = 91)	% (n = 119)	% (n = 110)	
Stunted												
No	85.9	90.5	95.6	0.035	82.6	95.2	92.6	0.005	79.1	96.6	96.4	<0.001
Yes	14.1	9.5	4.4		17.4	4.8	7.4		20.9	3.4	3.6	
Underweight												
No	91.9	96	96.3	0.254	79.4	92	93.4	0.002	72.5	95	97.3	<0.001
Yes	8.1	4	3.7		20.7	8	6.6		27.5	5	2.7	
Wasted												
No	95	95.2	94.8	0.987	88	96.8	95.9	0.015	85.7	95	98.2	0.001
Yes	5	4.8	5.2		12	3.2	4.1		14.3	5	1.8	

^a^*p*-values were calculated for differences in proportions using chi-square

^┼^WHO child growth indicators

### The Care Group intervention, maternal social support, and infant growth

The interaction and independent effects of the Care Group intervention and maternal social support were evaluated regarding infant stunting, underweight, and wasting (S1a–S1c Table in S2 File).

### Effects of the Care Group intervention and maternal social support interaction on infant LAZ

There was a significant interaction between the Care Group intervention and maternal social support over time on infant mean LAZ (F _(6, 560)_ = 28.91, *p* < 0.001) (S1a Table in S2 File). The interaction had a large effect size (*f* = 2.4) [51], and explained 85 percent (${\hat{\omega }}_{Y|A.B}^{2}$ = 0.85) of the variability in infant mean LAZ among the study arms after accounting for individual effects (S3 Table in S2 File).

**Fig 2:**
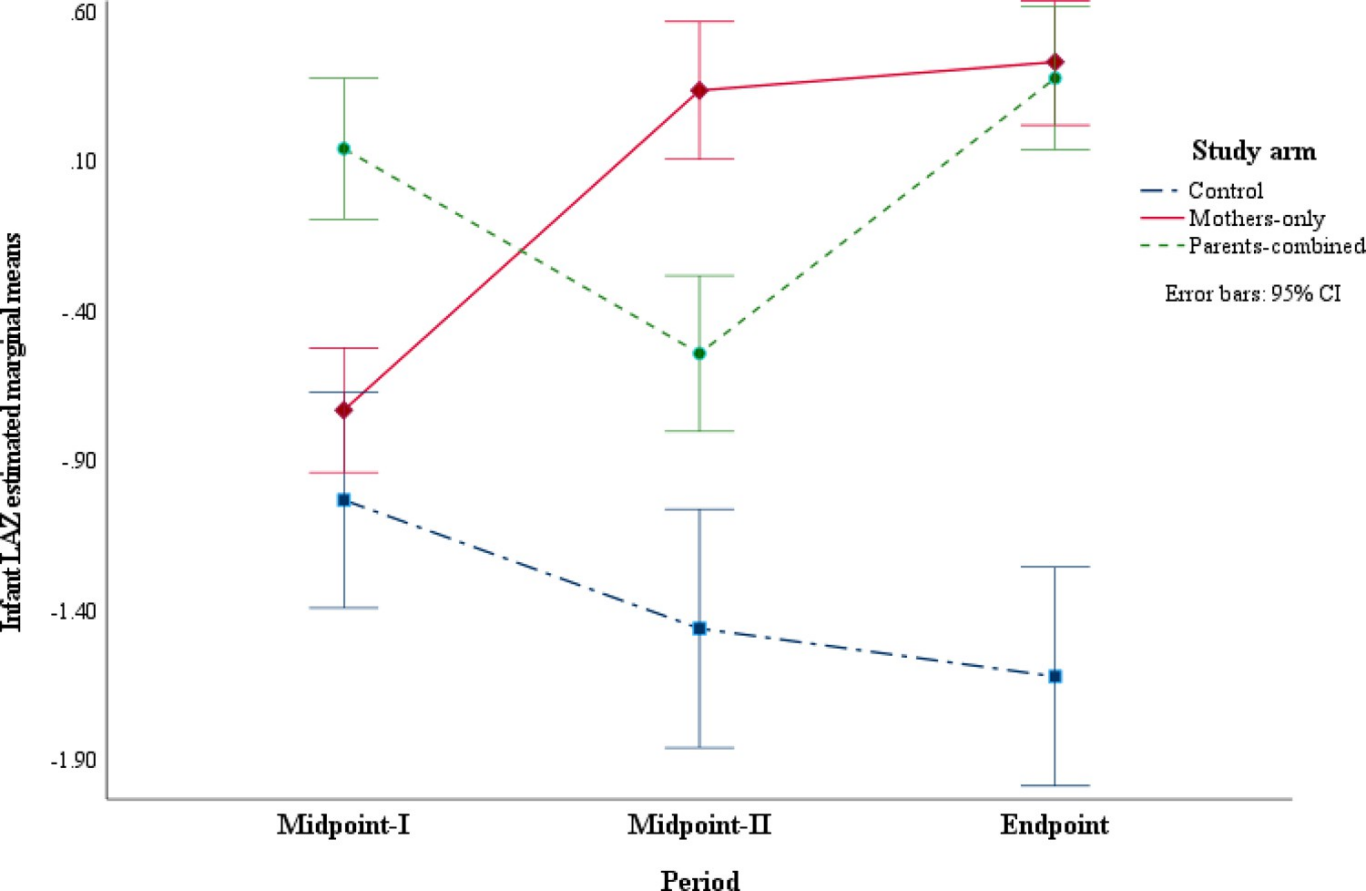
Infant length-for-age z-scores (LAZ) for the study arm with social support by time interaction.

Simple main effects were further investigated with pairwise comparisons for the study arm with maternal social support by time interaction (Table 3) and for time by study arm with maternal social support (Table 4). As illustrated in Fig 2, infants’ mean LAZ in the Mothers-only study arm at Midpoint-II were improved (mean difference (*MD*) = 1.07, *p* < 0.001) compared to Midpoint-I. The infant mean LAZ at the Endpoint was also higher than that at Midpoint-I in the Mothers-only arm (*MD* = 1.16, *p* < 0.001). Infants in the Parents-combined arm experienced a decrease in mean LAZ between the Midpoint-I and Midpoint-II periods (*MD* = -0.68, *p* < 0.001). However, these LAZ scores improved between Midpoint-II and Endpoint (*MD* = 0.92, *p* < 0.001). The infants’ mean LAZ scores in the Control arm decreased across successive times, Midpoint-I to Endpoint (*MD* = -0.59, *p* = 0.011).

**Table 3:** Simple main effects of the study arm and social support-time interaction on infant mean LAZ.

Study arm	(I) Time	(J) Time	(I-J)^b^	S.E	95% CI^a^	
					Lower Bound	Upper Bound
Mothers-only	Midpoint-II	Midpoint-I	1.07***	0.13	0.76	1.37
		Endpoint	-0.10	0.11	-0.36	0.17
	Endpoint	Midpoint-I	1.16***	0.12	0.88	1.44
		Midpoint-II	0.10	0.11	-0.17	0.36
Parents-combined	Midpoint-II	Midpoint-I	-0.68***	0.14	-1.03	-0.34
		Endpoint	-0.92***	0.13	-1.22	-0.62
	Endpoint	Midpoint-I	0.24	0.13	-0.08	0.55
		Midpoint-II	0.92***	0.13	0.62	1.22
Control	Midpoint-II	Midpoint-I	-0.43	0.22	-0.96	0.10
		Endpoint	0.16	0.19	-0.31	0.63
	Endpoint	Midpoint-I	-0.59*	0.20	-1.08	-0.10
		Midpoint-II	-0.16	0.19	-0.63	0.31

^a^95 percent confidence intervals; S.E standard error; Tukey Kramer correction applied

^b^ Mean LAZ differences included interactive effects of maternal social support in Study arm

* *p* < .05

** *p* < .01

*** *p* < .001

**Table 4:** Simple main effects of the time-study arm and social support interaction on infant mean LAZ.

Time	(I) Study arm	(J) Study arm	(I-J)^b^	S.E	95% CI^a^	
					Lower Bound	Upper Bound
Midpoint-I	Mothers-only	Control	0.30	0.21	-0.21	0.81
		Parents-combined	-0.87***	0.16	-1.26	-0.49
	Parents-combined	Control	1.17***	0.24	0.59	1.76
		Mothers-only	0.87***	0.16	0.49	1.26
Midpoint-II	Mothers-only	Control	1.80***	0.24	1.23	2.36
		Parents-combined	0.88***	0.18	0.45	1.31
	Parents-combined	Control	0.92**	0.27	0.27	1.57
		Mothers-only	-0.88***	0.18	-1.31	-0.45
Endpoint	Mothers-only	Control	2.05***	0.22	1.53	2.57
		Parents-combined	0.05	0.16	-0.34	0.45
	Parents-combined	Control	2.00***	0.25	1.40	2.59
		Mothers-only	-0.05	0.16	-0.45	0.34

^a^95 percent confidence intervals; S.E standard error; Tukey Kramer correction applied

^b^ Mean LAZ differences included interactive effects of maternal social support in Study arm

* *p* < .05

** *p* < .01

*** *p* < .001

The simple main effects of time by study arms with maternal social support (Table 4) showed that at Midpoint-I, the infants’ mean LAZ was higher in the Parents-combined arm than the Control (*MD* = 1.17, *p* < 0.001) and higher than in the Mothers-only arm (*MD* = 0.87, *p* < 0.001). At Midpoint-II, infants in both the Mothers-only (*MD* = 1.80, *p* < 0.001) and the Parents-combined (*MD* = 0.92, *p* < 0.001) arms had higher mean LAZ scores compared to the Control. However, mean LAZ scores in the Mothers-only study arm were higher than in the Parents-combined arm (*MD* = 0.88, *p* < 0.001). At the end of the study, infants’ mean LAZ was higher in both the Mothers-only (*MD* = 2.05, *p* < 0.001) and Parents-combined (*MD* = 2.00, *p* < 0.001) arms than the Control arm. Overall, by the end of the study, infants from both intervention study arms had significantly higher mean LAZ scores than those from the Control arm. However, there were no significant differences in the infant mean LAZ between the intervention study arms.

### Effect of the intervention and social support on infant weight-for-age z-scores (WAZ)

**Fig 3:**
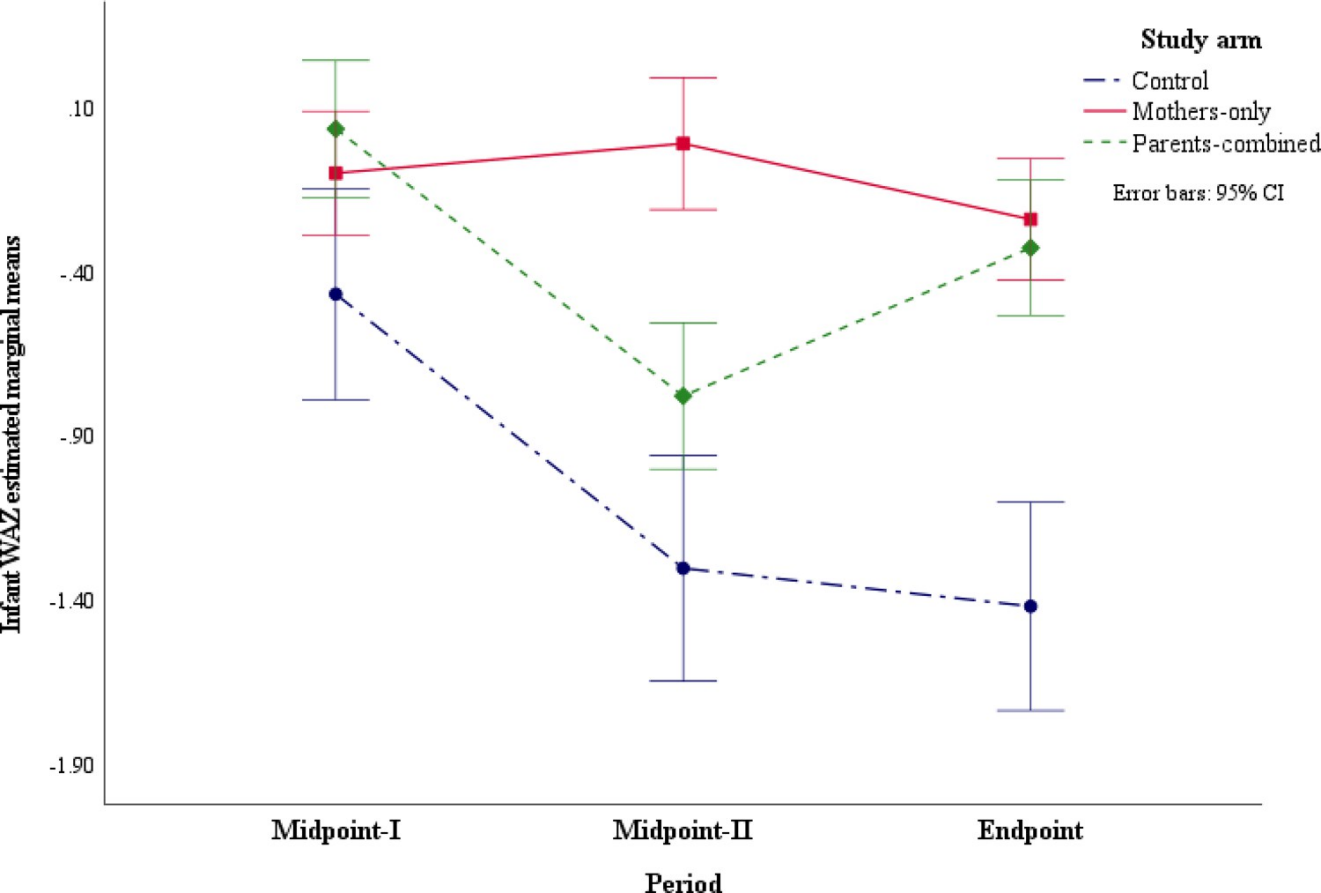
Infant weight-for-age z-scores (WAZ) for the study arm with social support by time interaction.

There was a significant interaction effect of maternal social support in the study arm by study period (F _(5.8, 539.4)_ = 12.70, *p* < 0.001) on infant mean WAZ (S1b Table in S2 File). The interaction had a large effect size (*f* = 1.6) [51] and explained 72 percent (${\hat{\omega }}_{Y|A.B}^{2}$ = 0.72) of the variability in infant mean WAZ (S3 Table in S2 File). Further, the simple main effects results in Table 5 showed that the infant mean WAZ in the Mothers-only study arm were significantly lower at Endpoint when compared to Midpoint-II (*MD* = -0.23, *p* = 0.022). In the Parents-combined arm, there was a decrease in the infant mean WAZ between Midpoint-I and Midpoint-II periods (*MD* = -0.82, *p* < 0.001). In contrast, the infant mean WAZ improved in the Parents-combined arm between Midpoint-II and the Endpoint period (*MD* = 0.48, *p* < 0.001). However, the infant mean WAZ at the Endpoint remained lower when compared to Midpoint-I (*MD* = -0.34, *p* < 0.001) in the Parents-combined study arm (Fig 3).

**Table 5:** Simple main effects of the study arm and social support-time interaction on infant mean WAZ.

Study arm	(I) Time	(J) Time	(I-J)^b^	S.E	95% CI^a^	
					Lower Bound	Upper Bound
Mothers-only	Midpoint-II	Midpoint-I	0.08	0.10	-0.16	0.32
		Endpoint	0.23*	0.09	0.03	0.44
	Endpoint	Midpoint-I	-0.15	0.09	-0.37	0.07
		Midpoint-II	-0.23*	0.09	-0.44	-0.03
Parents-combined	Midpoint-II	Midpoint-I	-0.82***	0.11	-1.10	-0.55
		Endpoint	-0.48***	0.10	-0.71	-0.25
	Endpoint	Midpoint-I	-0.34**	0.10	-0.59	-0.10
		Midpoint-II	0.48***	0.10	0.25	0.71
Control	Midpoint-II	Midpoint-I	-0.80***	0.18	-1.22	-0.38
		Endpoint	0.17	0.15	-0.19	0.52
	Endpoint	Midpoint-I	-0.96***	0.16	-1.34	-0.58
		Midpoint-II	-0.17	0.15	-0.52	0.19

^a^95 percent confidence intervals; S.E standard error; Tukey Kramer correction applied

^b^ Mean WAZ differences included interactive effects of maternal social support in Study arm

* *p* < .05

** *p* < .01

*** *p* < .001

The results in Table 6 showed that at Midpoint-I, there was a higher infant mean WAZ in both the Mothers-only (*MD* = 0.46, *p* = 0.042) and Parents-combined (*MD* = 0.67, *p* = 0.006) study arms than the Control. Also, at Midpoint-II, infants in both the Mothers-only (*MD* = 1.33, *p* < 0.001) and Parents-combined (*MD* = 0.64, *p* = 0.024) arms had higher mean WAZ than the Control. However, the Mothers-only study arm had a greater infant mean WAZ than infants in the Parents-combined arm (*MD* = 0.69, *p* < 0.001). At the Endpoint period, infant mean WAZ was greater in both the Mothers-only (*MD* = 1.27, *p* < 0.001) and the Parents-combined (*MD* = 1.28, *p* < 0.001) study arms compared to the Control. Overall, infant mean WAZ scores in both intervention study arms were significantly greater than the Control at all study periods. However, by the end of the study, there were no significant differences in the infant mean WAZ between the intervention study arms.

**Table 6:** Simple main effects of the time-study arm and social support interaction on infant mean WAZ.

Time	(I) Study arm	(J) Study arm	(I-J)^b^	S.E	95% CI^a^	
					Lower Bound	Upper Bound
Midpoint-I	Mothers-only	Control	0.46*	0.19	0.01	0.90
		Parents-combined	-0.21	0.14	-0.55	0.13
	Parents-combined	Control	0.67**	0.21	0.15	1.18
		Mothers-only	0.21	0.14	-0.13	0.55
Midpoint-II	Mothers-only	Control	1.33***	0.21	0.83	1.83
		Parents-combined	0.69***	0.16	0.31	1.07
	Parents-combined	Control	0.64*	0.24	0.06	1.21
		Mothers-only	-0.69***	0.16	-1.07	-0.31
Endpoint	Mothers-only	Control	1.27***	0.19	0.82	1.71
		Parents-combined	-0.02	0.14	-0.36	0.32
	Parents-combined	Control	1.28***	0.21	0.77	1.80
		Mothers-only	0.02	0.14	-0.32	0.36

^a^95 percent confidence intervals; S.E standard error; Tukey Kramer correction applied

^b^ Mean WAZ differences included interactive effects of maternal social support in Study arm

* *p* < .05

** *p* < .01

*** *p* < .001

### Effect of the peer-led intervention and maternal social support on infant weight-for-length z-scores (WLZ)

**Fig 4:**
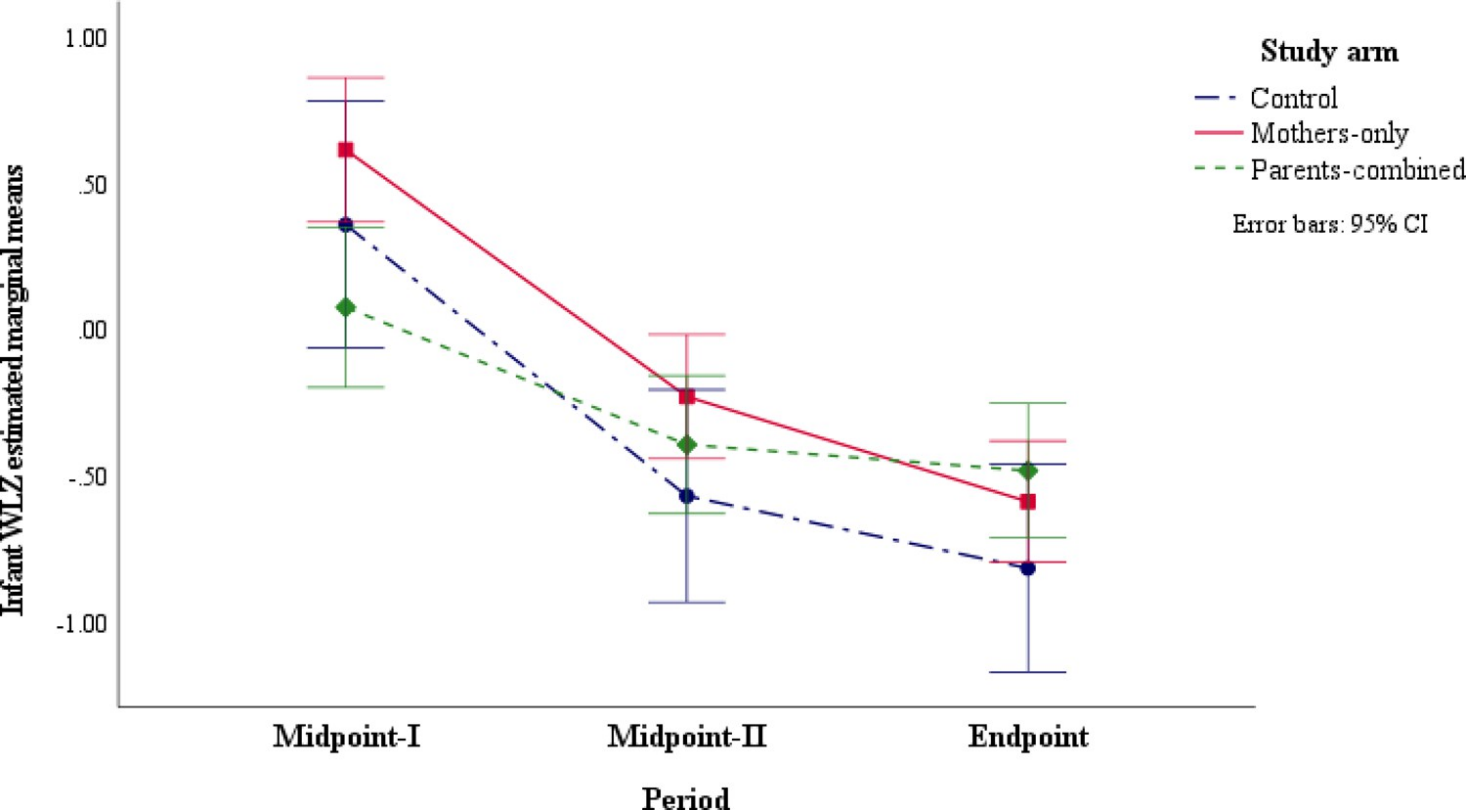
Infant weight-for-length z-scores (WLZ) for the study arm with social support by time interaction.

The results in S1c Table in S2 File showed a significant interaction effect of maternal social support in study arms by period (F _(5.3, 492.5)_ = 3.38, *p* = 0.004). With a medium effects size (*f* = 0.72) [51], the interaction effect explained 34 percent (${\hat{\omega }}_{Y|A.B}^{2}$ = 0.34) of the variability in infant mean WLZ (Table S3 in S2 File). As shown in Fig 4 and Table 7, infants’ mean WLZ in the Mothers-only study arm were reduced between both the Midpoint-I and Midpoint-II periods (*MD* = -0.85, *p* = <0.001) and from Midpoint-II and Endpoint (*MD* = -0.37, *p* = 0.001). Also, the infant mean WLZ at the Endpoint was lower than the mean WLZ at Midpoint-I (*MD* = -1.22 *p* < 0.001). Similarly, the infant mean WLZ in the Parents-combined arm was lessened between Midpoint-I and Midpoint-II periods (*MD* = -0.45, *p* = 0.017) and at the Endpoint compared to the Midpoint-I period (*MD* = -0.52, *p* = 0.002). The results in Table 7 also showed that infant mean WLZ in the Control was significantly reduced from Midpoint-I to Midpoint-II (*MD* = -0.96, *p* < 0.001) and between the Endpoint and Midpoint-I period (*MD* = -1.21, *p* < 0.001). However, there was no significant change in the infant mean WLZ scores between the Midpoint-II and Endpoint periods in the Control arm.

**Table 7:** Simple main effects of the study arm and social support -time interaction on infant mean WLZ.

Study arm	(I) Time	(J) Time	(I-J)^b^	S.E	95% CI^a^	
					Lower Bound	Upper Bound
Mothers-only	Midpoint-II	Midpoint-I	-0.85***	0.14	-1.19	-0.50
		Endpoint	0.37**	0.10	0.13	0.62
	Endpoint	Midpoint-I	-1.22***	0.13	-1.54	-0.90
		Midpoint-II	-0.37**	0.10	-0.62	-0.13
Parents-combined	Midpoint-II	Midpoint-I	-0.45*	0.16	-0.83	-0.06
		Endpoint	0.08	0.11	-0.20	0.35
	Endpoint	Midpoint-I	-0.52**	0.15	-0.89	-0.16
		Midpoint-II	-0.08	0.11	-0.35	0.20
Control	Midpoint-II	Midpoint-I	-0.96***	0.25	-1.55	-0.37
		Endpoint	0.25	0.18	-0.18	0.67
	Endpoint	Midpoint-I	-1.21***	0.23	-1.77	-0.65
		Midpoint-II	-0.25	0.18	-0.67	0.18

^a^95 percent confidence intervals; S.E standard error; Tukey Kramer correction applied

^b^ Mean WLZ differences included interactive effects of maternal social support in Study arm

* *p* < .05

** *p* < .01

*** *p* < .001

Further, this study showed in Table 8 that at Midpoint-I, there were no significant differences in the infant mean WLZ between the intervention study arms and the Control arm. Though infants in the Mothers-only study arm had higher mean WLZ than the Parents-combined arm at Midpoint-I (*MD* = 0.61, *p* = 0.006), by the end of the study, infants in all study arms had no significant differences in the mean WLZ scores.

**Table 8:** Simple main effects of the time-study arm and social support interaction on infant mean WLZ.

Time	(I) Study arm	(J) Study arm	(I-J)^b^	S.E	95% CI^a^	
					Lower Bound	Upper Bound
Midpoint-I	Mothers-only	Control	0.23	0.26	-0.39	0.85
		Parents-combined	0.61*	0.20	0.14	1.08
	Parents-combined	Control	-0.38	0.30	-1.09	0.33
		Mothers-only	-0.61*	0.20	-1.08	-0.14
Midpoint-II	Mothers-only	Control	0.35	0.22	-0.19	0.88
		Parents-combined	0.21	0.17	-0.19	0.62
	Parents-combined	Control	0.13	0.26	-0.48	0.75
		Mothers-only	-0.21	0.17	-0.62	0.19
Endpoint	Mothers-only	Control	0.22	0.21	-0.29	0.73
		Parents-combined	-0.09	0.16	-0.47	0.30
	Parents-combined	Control	0.30	0.24	-0.28	0.89
		Mothers-only	0.09	0.16	-0.30	0.47

^a^95 percent confidence intervals; S.E standard error; Tukey Kramer correction applied

^b^ Mean WLZ differences included interactive effects of maternal social support in Study arm

* *p* < .05, ** *p* < .01, *** *p* < .001

In summary, significant interaction effects of maternal social support and Care Group by study period were observed with infant mean LAZ, WAZ, and WLZ. By the end of the study, the Mothers-only and Parents-combined study arms had infants with higher mean LAZ and WAZ but not WLZ compared to the Control arm. By the end of the study, no significant differences were observed between the Mothers-only and Parents-combined arms for infant LAZ and WAZ.

## Discussion

This year-long community RCT examined the effectiveness of a peer-led integrated nutrition education intervention on infant growth using Care Groups among refugees in the West Nile region in Uganda. We showed that the Care Group intervention had significant interaction effects with maternal social support on infant mean LAZ, WAZ, and WLZ scores over the study period. By the end of our study, in the intervention study arms (Mothers-only and Parents-combined), infant stunting (<5%) and underweight (<10%) were low, while wasting ranged from medium to very low (≤ 5%). However, in the Control arm, stunting (20.9%), underweight (27.5%), and wasting (14.3%) were high according to WHO guidelines [52].

By the end of the study the interaction effects of the Care Group intervention and maternal social support significantly improved infant mean LAZ in both intervention study arms compared to the infants in the Control arm. Further, the findings demonstrated a higher maternal social support score in study arms participating in the Care Group intervention arms than in the Control arm. The Care Group intervention provided a peer-assisted nutrition education learning platform through biweekly meetings and cooking demonstrations on preparing complementary foods. Further, engagement in backyard vegetable gardening advanced infant feeding knowledge and practices, and maternal social support improved infant linear growth. The findings in our study were comparable to the results of two replications of the Care Group model in Mozambique [53] that reported a significant decrease in stunting of children under five years of age among communities that participated in a participatory nutrition intervention using the Care Group model.

A cluster-randomized trial (CRT) in Peru [54] and one in India [26] likewise demonstrated that children of caretakers who participated in group sessions of nutritional counseling and demonstrations of complementary foods preparation had better LAZ than children in control areas. One difference between our study and the Peruvian and Indian CRTs included the local context unique to the refugee situation. Additionally, the caretakers’ training in Peru was done at health facilities by health workers rather than by peer-lead volunteers using the Care Group model as done in our study. Our intervention empowered mothers to impact their peers by becoming health educators, thus leveraging the health workers’ responsibility mostly towards skilled care to patients in the health facilities. Further, a mixed-methods cross-sectional study in Thailand [55] conducted among refugee mothers of infants (2–12 months) in Mae La camp attributed improved infant linear growth to the increased social support for mothers provided through programs by non-governmental organizations. Our study based on peer-led integrated nutrition education with the Care Group model emphasized group cohesion and peer visits as part of group dynamics [56], evidenced by the increased social support scores in the treatment arms. Therefore, based on our findings, we expect that engaging mothers or child caretakers in a Care Group intervention over a longer period would sustainably improve infant linear growth among refugee children in post-emergency settlements.

The mean infant WAZ in our study was improved by the interaction between the Care Group and maternal social support over the study period. By the end of the study, both intervention study arms had higher infant mean WAZ compared to the Control. Our findings were consistent with a systematic review of randomized and non-randomized studies in developing countries [24], which reported significantly higher infant mean WAZ if caretakers had participated in education interventions on complementary feeding practices for infants. However, a recent meta-analysis of three peer-group nutrition intervention studies [57] in LMICs showed that the peer-group interventions among mothers had only 24 percent reduction in the likelihood for underweight children. In that metanalysis, the participation in backyard gardening for Care Group members and the training modules on the value of foods improved both the skills and knowledge on nutrition to improve infant mean weight. A study in Kenya [58] explained that emphasis on Care Groups and setting up kitchen gardens was key in reducing undernutrition including micronutrient deficiencies among children.

By the end of our study, a general decline in the infant mean WAZ was observed in all study arms. The impacts of the Care Group over time in our intervention on infant growth may have been affected by the COVID-19 pandemic. The study period coincided with the peak period of the global COVID-19 pandemic; thus, standard operating procedures (SOPs) were adopted for safety to mitigate the potential spread, and extra social interactions beyond the Care Group meetings such as the peer-to-peer home visits may have been limited. Also, face masks and distancing during Care Group meetings may have had physical or psychological effects on the perceived social support. A cross-sectional study in Taiwan [59] reported that restrictions on interactions among individuals were significantly associated with reduced perceptions of social support due to voluntary interaction reduction even when the risk of contracting the virus was lower based on demographics. In our study, even though better maternal social support scores were observed in the study arms that participated in the integrated intervention, the mean social support scores were moderate with a high standard deviation indicating variability in the perceived maternal social support in all study arms. Amidst these findings, the positive effects of our findings on infant mean WAZ established that the integrated intervention through Care Groups was a viable strategy to improve infant underweight; longer interventions may be beneficial.

Interaction effects of maternal social support and the Care Group intervention had significant effects on infant mean WLZ. However, by the end of the study, no significant differences were observed between the mean WLZ among the study arms. A longer study in Peru [54] of 187 infants enrolled from birth to 18 months of age observed significantly improved mean changes in WLZ over the study period within the intervention area compared to the control area. Behavioral communication change interventions may require a longer time [60] to positively transform participant barriers toward better infant feeding knowledge, attitudes, and practices (KAPs) for improved WLZ among infants.

Our findings were similar to the outcomes of a community-based household survey in Uganda [61], which reported that maternal social support was not associated with child wasting. Similarly, a meta-analysis [57] determined a non-significant positive effect of the group interventions on child wasting, explained by limited time for interventions to cause change in child growth. A systematic review of child growth in developing countries [24] likewise reported non-significant associations on WLZ based on nutritional education interventions. The reasons posited aligned with the recommendation for an integrated nutrition education intervention with the provision of affordable complementary foods, especially in food-insecure areas. Increasing availability, access, and utilization of food through integrating livelihoods and food security programs in the Care Group model may provide better results for overall child growth. Integrated multisectoral approaches of both direct and indirect programs that are nutrition-focused, for example, education, livelihoods, market subsidies, the Care Group approach, and women’s empowerment, to mention but a few, provide a holistic strategy to address underlying determinants of child malnutrition [14].

Our study provided cooking demonstrations using locally available foods and affordable recipe options from the markets within refugee settlements as part of complementary food preparation training. Further, Care Groups, as part of the intervention, participated in backyard gardening of vegetables and nutrition education training expected to improve infant growth; however, variability in maternal social support within the treatment arms may have contributed to the non-significance in the intervention interaction effects on infant WLZ. Care Groups enabled peer mentoring and targeted the behavior of individuals, although social support is unique to individual behavior and perception. Therefore, mothers within a Care Group may have had different perceptions of social support based on individual expectations, creating variability in the reported social support scores [62].

Also, refugee household dietary practices may have been affected by food insecurity. By the end of the study, the households in our study reported a high level of food insecurity which may have affected infant complementary feeding and growth. Although refugees engaged in farming on the plots around homesteads and on communal agricultural land provided through the Government of Uganda [63], and some food rations were provided by UNHCR, COVID-19 prevention measures such as lockdown may have affected household livelihoods that usually increased access to food sources. Also, the large household sizes reported in our study suggest a need for more food sources to meet the nutritional needs of all family members in addition to the complementary feeding of infants [64]. Further, both short-term and long-term child growth may be affected by enteropathies that have a high incidence in protracted refugee settings due to congestion in the settlements and households and limited sources of safe water [65]. Based on our findings, we agree with previous literature [14, 57, 60] that our peer-led integrated nutrition intervention via Care Groups may have required more time for the effects of the intervention to fully impact the growth of children. Also, the COVID-19 pandemic may have exacerbated the need for more time, especially with its interruptions to the implementation of the study.

To our knowledge, this study was the first to examine the effectiveness of a peer-led integrated nutrition intervention using the Care Group model on growth among infants in refugee post-emergency settlements. The strengths of this study included the use of a RCT design which allowed us to establish causation. Also, this study used valid and reliable scales to assess infant growth and maternal social support. Even though the MOS support scale had not previously been used for studies in a refugee setting, our findings were consistent with studies conducted in rural communities in LMICs [58, 61, 66]. Further, the study had a large sample size that provided a good representation of South Sudanese refugees in the West Nile region in Uganda. However, our study may have been impacted by the COVID-19 pandemic rules that reduced the Care Groups of 10–20 to only five to ten for a limited part of the intervention time. Also, the measure of maternal social support was based on the mother’s perception and may not be exempt from social desirability bias [67].

Despite these limitations, the unique findings of this study provided evidence of the efficacy of the behavioral change Care Group intervention with maternal social support on the reduction of infant undernutrition among refugees in post-emergency settlements. The Care Group model has been suggested to fit within the standard government health structure under the Ministry of Health [68]. The Refugee and Host Population Empowerment (ReHope) strategy [63] integrates all community services with the host population to refugees within the settlements. Although, a longer duration may be beneficial, integrating the Care Group model in nutrition and health programs in post-emergency refugee communities could provide a sustainable community-centered approach to reducing child undernutrition.

## Conclusion

This study demonstrated that a peer-led integrated nutrition education intervention using the Care Group model with maternal social support improved infant stunting and underweight. The Care Group model as a behavioral communication change strategy may provide parents support by peers to recognize health challenges and work towards improvement. This study advances our knowledge of the interactions of the Care Group model and the proximal interpersonal relations through maternal social support that promote infant growth before their first birthday. Our findings provide additional evidence for a potentially sustainable approach to reducing infant undernutrition among refugees in post-emergency settlements, that may easily be integrated into ecologically similar communities.

## Supporting information

S1 Checklist Reporting checklist for randomised trial. (DOCX)

S2 Checklist *PLOS ONE* clinical studies checklist. (DOCX)

S3 Checklist STROBE statement—checklist of items that should be included in reports of observational studies. (DOCX)

S1 Fig Study Gantt chart. (TIF)

S1 File Research plan. (PDF)

S2 File (DOCX)
