# Supplementary material for: Care groups in an integrated nutrition education intervention improved infant growth among South Sudanese refugees in Uganda’s West Nile post-emergency settlements: A cluster randomized trial
Source: PLoS One. 2024 Mar 15;19(3):e0300334. doi: 10.1371/journal.pone.0300334 (PMC10942045; doi:10.1371/journal.pone.0300334)
Supplement: S3 Checklist — (DOCX) [file pone.0300334.s003.docx]

STROBE Statement—checklist of items that should be included in reports of observational studies

|  | Item No. | Recommendation | Page  No. | Relevant text from manuscript |
| --- | --- | --- | --- | --- |
| **Title and abstract** | 1 | (*a*) Indicate the study’s design with a commonly used term in the title or the abstract | 2, 4 – 5 | Abstract, methods, study design |
|  |  | (*b*) Provide in the abstract an informative and balanced summary of what was done and what was found | 2 | Abstract |
| Introduction | | | |  |
| Background/rationale | 2 | Explain the scientific background and rationale for the investigation being reported | 3 – 4 | Introduction, Lines 58 – 67 |
| Objectives | 3 | State specific objectives, including any prespecified hypotheses | 4 | Introduction, Lines 62 – 67 |
| Methods | | | |  |
| Study design | 4 | Present key elements of study design early in the paper | 2, 4 | Study design |
| Setting | 5 | Describe the setting, locations, and relevant dates, including periods of recruitment, exposure, follow-up, and data collection | 4 – 5 | Study design and setting |
| Participants | 6 | 1. *Cohort study*—Give the eligibility criteria, and the sources and methods of selection of participants. Describe methods of follow-up   *Case-control study*—Give the eligibility criteria, and the sources and methods of case ascertainment and control selection. Give the rationale for the choice of cases and controls  *Cross-sectional study*—Give the eligibility criteria, and the sources and methods of selection of participants | 5 – 6 | Study design, inclusion & exclusion |
|  |  | (*b*) *Cohort study*—For matched studies, give matching criteria and number of exposed and unexposed *(This study used community-based matching criteria by the settlements in which the refugees resided)*  *Case-control study*—For matched studies, give matching criteria and the number of controls per case | 5, 6 – 7, Figure 1 | Methods; Lines 77 – 84; Lines 91 – 101, Lines 112 – 134 |
| Variables | 7 | Clearly define all outcomes, exposures, predictors, potential confounders, and effect modifiers. Give diagnostic criteria, if applicable | 8 | Study measures |
| Data sources/ measurement | 8* | For each variable of interest, give sources of data and details of methods of assessment (measurement). Describe comparability of assessment methods if there is more than one group | 8 | Study measures |
| Bias | 9 | Describe any efforts to address potential sources of bias | 4 - 5  10  26 - 27 | Randomization; Lines 70 – 82  Adjusting for confounders; Lines 195 – 201  Limitations; Lines 442 – 445 |
| Study size | 10 | Explain how the study size was arrived at | 5 – 6 | Sample size |

| Quantitative variables | 11 | Explain how quantitative variables were handled in the analyses. If applicable, describe which groupings were chosen and why | 8 – 9 | Study Measures, Statistical analyses |
| --- | --- | --- | --- | --- |
| Statistical methods | 12 | (*a*) Describe all statistical methods, including those used to control for confounding | 9 | Statistical analyses |
|  |  | (*b*) Describe any methods used to examine subgroups and interactions | 9 | Statistical analyses |
|  |  | (*c*) Explain how missing data were addressed *(Allocation for a 23 percent loss during follow-up was made to maintain the power (0.90) for analyses. Also, the exclusion/ inclusion criteria throughout the study was described with final criteria for data utilization explained)* | 5 – 6, Figure 1 | Lines 112 – 118 |
|  |  | (*d*) *Cohort study*—If applicable, explain how loss to follow-up was addressed  *Case-control study*—If applicable, explain how matching of cases and controls was addressed | 5, 6 | Loss due to follow-up, inclusion & Exclusion |
|  |  | (*e*) Describe any sensitivity analyses *(Interaction effects among the variables were determined using the split plot factor analyses; further, effects sizes calculated showed the magnitude of difference in the dependent variable attributed to the interaction effects of the intervention)* | 9 - 10, 16,18, 20 | Statistical analyses, interaction effects |
| Results | | | | |
| Participants | 13* | (a) Report numbers of individuals at each stage of study—eg numbers potentially eligible, examined for eligibility, confirmed eligible, included in the study, completing follow-up, and analysed *(See line 116 for mention of the flow diagram labelled Figure 1)* | 5, 6 | Shown/ Reported in Figure 1  Lines 92 – 103 |
|  |  | (b) Give reasons for non-participation at each stage | 6 | Shown/ Reported in Figure 1 |
|  |  | © Consider use of a flow diagram *(A flow diagram labelled Figure 1, submitted separately as requested)* | 6 | Shown/ Reported in Figure 1 |
| Descriptive data | 14* | (a) Give characteristics of study participants (eg demographic, clinical, social) and information on exposures and potential confounders | 10 – 15 | Results; Lines 191 – 229; Table 1, and 2  Adjusting for confounders; Lines 195 – 201 |
|  |  | (b) Indicate number of participants with missing data for each variable of interest *(Due to intensive follow-up with participants, with participant consent, all data for variables of interest for analyses in this manuscript were complete)* | N/A |  |
|  |  | © *Cohort study*—Summarise follow-up time (eg, average and total amount) | 6 | Shown/ Reported in Figure 1 |
| Outcome data | 15* | *Cohort study*—Report numbers of outcome events or summary measures over time | 6 | Shown/ Reported in Figure 1 |
|  |  | *Case-control study—*Report numbers in each exposure category, or summary measures of exposure | *6* | Shown/ Reported in Figure 1 |
|  |  | *Cross-sectional study—*Report numbers of outcome events or summary measures | *N/A* |  |
| Main results | 16 | (*a*) Give unadjusted estimates and, if applicable, confounder-adjusted estimates and their precision (eg, 95% confidence interval). Make clear which confounders were adjusted for and why they were included | 11 – 22 | Results  Adjusting for confounders; Lines 195 – 201 |
|  |  | (*b*) Report category boundaries when continuous variables were categorized | 12 – 15 | Results; Line 225 – 253 |
|  |  | © If relevant, consider translating estimates of relative risk into absolute risk for a meaningful time period | N/A |  |
| Other analyses | 17 | Report other analyses done—e.g. analyses of subgroups and interactions, and sensitivity analyses | 16 – 22 | Results; Lines 259 – 358 |
| Discussion |  |  |  |  |
| Key results | 18 | Summarise key results with reference to study objectives | 2 | Abstract; Lines 14 – 22 |
| Limitations | 19 | Discuss limitations of the study, taking into account sources of potential bias or imprecision. Discuss both direction and magnitude of any potential bias | 26 | Limitations; Lines 442 – 445 |
| Interpretation | 20 | Give a cautious overall interpretation of results considering objectives, limitations, multiplicity of analyses, results from similar studies, and other relevant evidence | 21 – 26 | Discussion |
| Generalisability | 21 | Discuss the generalisability (external validity) of the study results | 26 – 27 | Lines 449 – 473 |
| Other information |  |  |  |  |
| Funding | 22 | Give the source of funding and the role of the funders for the present study and, if applicable, for the original study on which the present article is based *(According to the submission guidelines, the funders and their role was submitted separately in the financial disclosure section as requested. However, just to point it out here, Name of funder: This research was funded by a grant from the* ***Nestlé Foundation for the Study of Problems of Nutrition in the World, Switzerland****. URL: https://www.nestlefoundation.org/* ***Grant Number****:* ***2336****. Funding statement: The funders had no role in study design, data collection and analysis, decision to publish, or preparation of the manuscript)* | Shown elsewhere in the manuscript submission packet | Indicated elsewhere according to author submission guidelines |

*Give information separately for cases and controls in case-control studies and, if applicable, for exposed and unexposed groups in cohort and cross-sectional studies.

**Note:** An Explanation and Elaboration article discusses each checklist item and gives methodological background and published examples of transparent reporting. The STROBE checklist is best used in conjunction with this article (freely available on the Web sites of PLoS Medicine at http://www.plosmedicine.org/, Annals of Internal Medicine at http://www.annals.org/, and Epidemiology at http://www.epidem.com/). Information on the STROBE Initiative is available at www.strobe-statement.org.
