# Supplementary figures and images for: Care groups in an integrated nutrition education intervention improved infant growth among South Sudanese refugees in Uganda’s West Nile post-emergency settlements: A cluster randomized trial
Source: PLoS One. 2024 Mar 15;19(3):e0300334. doi: 10.1371/journal.pone.0300334 (PMC10942045; doi:10.1371/journal.pone.0300334)

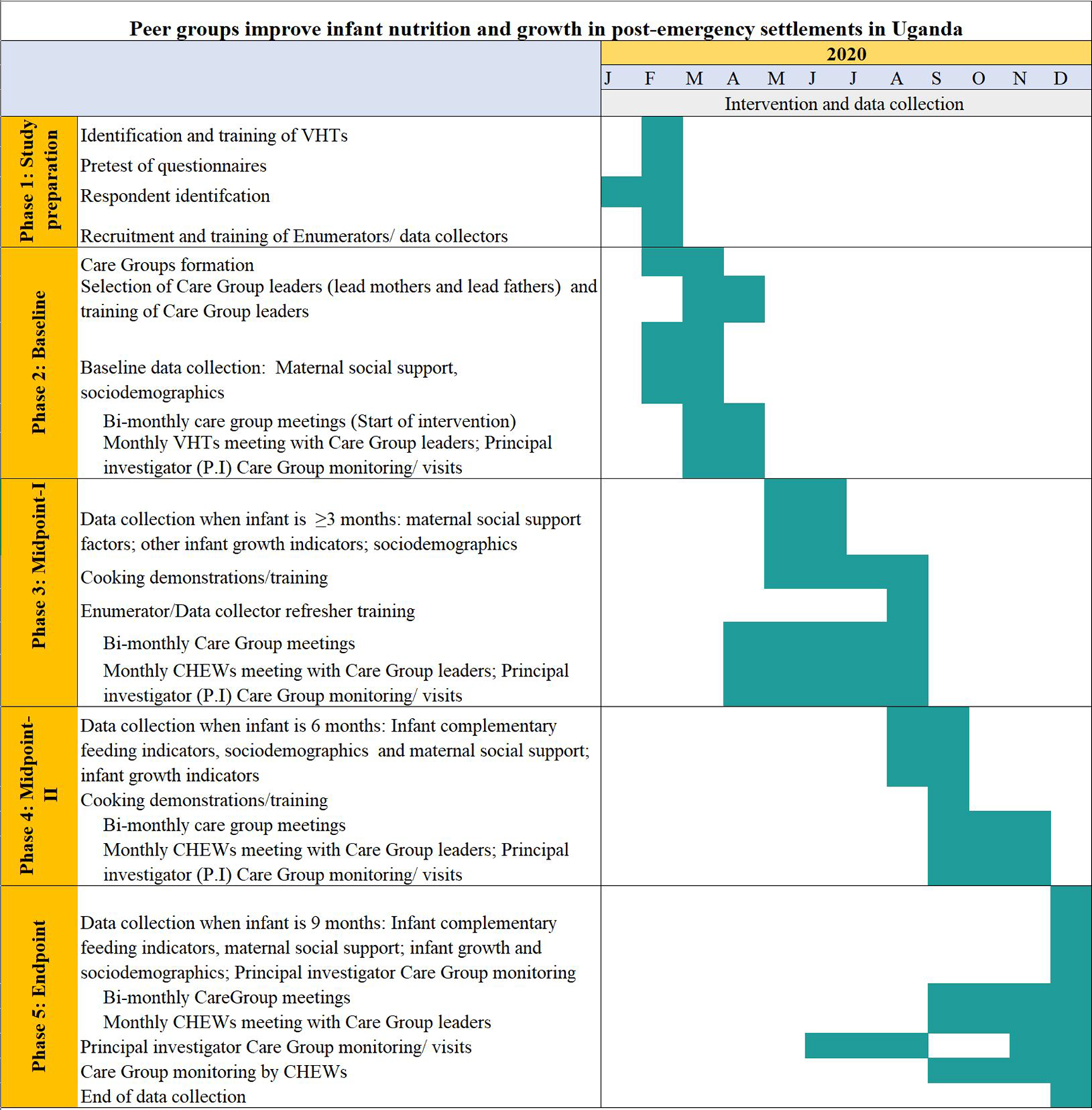

Supplement: S1 Fig — (TIF) [file pone.0300334.s004.tif]
