# Supplementary material for: Care groups in an integrated nutrition education intervention improved infant growth among South Sudanese refugees in Uganda’s West Nile post-emergency settlements: A cluster randomized trial
Source: PLoS One. 2024 Mar 15;19(3):e0300334. doi: 10.1371/journal.pone.0300334 (PMC10942045; doi:10.1371/journal.pone.0300334)
Supplement: S1 File — (PDF) [file pone.0300334.s005.pdf]

School of Health Sciences Research and Ethics Committee (MakSHS-REC)

RESEARCH PLAN

Name of Principal Investigator: Joel J. Komakech

Study Title: Peer groups to improve feeding practices and child nutrition in post-emergency settlements in Uganda

Study Version Number/Date:

1. Aims/objectives/research question/hypothesis:

General goal.

The goal of this study is to test the effectiveness of an integrated nutrition intervention delivered through care groups in improving infant and young child feeding practices, child growth and development and maternal mental health.

Specific objectives

- Design a context-specific and culturally appropriate integrated intervention that will address poor infant feeding practices, suboptimal basic hygiene, adequate child stimulation, and mental health
- Investigate the effects of the integrated intervention delivered through care groups on child length and weight
- Investigate the effects of the integrated intervention delivered through care groups on breastfeeding and complementary feeding practices of post-emergency settlement mothers
- Determine whether fathers' participation in care groups has additive benefits on infant and young child feeding practices of post-emergency settlement mothers
- Evaluate the effects of the integrated intervention delivered through care groups on child development
- Assess the association between the mental health of post-emergency settlement mothers and their child's nutritional status and development

Research questions

1. Will an integrated intervention combining nutrition, basic hygiene, child stimulation, and social support improve young refugee children's nutritional status?
2. Will infant and young child feeding (IYCF) practices of refugee mothers improve if they participate in care groups?
3. Will fathers' participation in care groups have additive benefits on infant and young child feeding practices of refugee mothers?
4. Will an integrated intervention combining nutrition, basic hygiene, child stimulation, and social support improve young refugee children's developmental outcomes?
5. Will parental participation in social support combined interventions with nutrition and basic hygiene improve maternal mental health status?

Hypotheses

An integrated intervention combining nutrition, basic hygiene, child stimulation, and social support will improve young refugee children's nutritional status.

Infant and young child feeding (IYCF) practices of refugee mothers will improve if they participate in care groups.

Fathers' participation in care groups will have additive benefits on infant and young child feeding practices of refugee mothers.

MAKERERE UNIVERSITY
SCHOOL OF HEALTH SCIENCES
APPROVED
VALID [illegible]
01 MAY 2020
RESEARCH & ETHICS COMMITTEE
P. O. BOX 7072, KAMPALA

An integrated intervention combining nutrition, basic hygiene, child stimulation and social support will improve young refugee children's developmental outcomes.

Parental participation in social support interventions combined with nutrition and basic hygiene will improve maternal mental health status.

2. Background and rationale:

Child malnutrition is prevalent in low- and middle-income countries (Black, *et al.*, 2008) with refugee children being even more vulnerable. Evidence from Côte d'Ivoire showed the negative impact of conflict on child health outcomes (Minoiu&Shemyakina, 2012; Dabalen&Saumik, 2014). In 2017, Uganda hosted over 1 million refugees (GoU & UNHCR, 2017). On average, a refugee family will stay in the post-emergency settlements for more than two decades while experiencing food insecurity (FAO, 2010). However, emergency aid is lacking when the refugees move into post-emergency settlements making children more likely to be undernourished. In the West Nile post-emergency settlements, high rates of global acute malnutrition (up to 11.8%) and stunting (up to 25%) are observed in children under 5. Additionally, the social support systems of refugees in post-emergency settlements have been disrupted because of their displacement (Buscher, 2013). Greater social support has been associated with better child breastfeeding and complementary feeding practices in low- and middle-income countries (LMICs)(Ziaei*et al.*, 2015; Mukuria*et al.*, 2016; Ickes *et al.*, 2018). Moreover, high levels of stress have been reported among Sudanese refugees, who are mostly located in the West Nile region (Karunakara*et al.*, 2004; Neuner *et al.*, 2004), and poor maternal mental health is associated with child undernutrition (Rahman *et al.*, 2008). Poor basic hygiene is also associated with poorer child growth (Cumming &Cairncross, 2016; Rakotomanana *et al.*, 2017).

Thus, there is a need for effective, sustainable nutrition interventions addressing these underlying determinants of child malnutrition. An integrated social support intervention addressing poor basic hygiene, inadequate child feeding, and child development practices, and mental health is expected to be effective in reducing child malnutrition among refugee children in the West Nile. Cost-effective and sustainable interventions are needed in the post-emergency settlements to prevent the long-term negative consequences of child undernutrition and impaired development. The care group model, a behavioral change communication strategy delivered by peers such as mothers to mothers or fathers to fathers, has a great potential for sustainability (Laughlin, 2004; Perry *et al.*, 2015). Briefly, a care group is composed of 10-15 neighboring mothers or fathers who meet bi-monthly to discuss topics in child health including nutrition. Each care group assisted by their community health extension worker (CHEW) will collectively nominate and select a care group leader (lead mother or a lead father), who will be trained by the CHEWs and will lead care group discussions/meetings that are expected to last 30 minutes to 2 hours on a bi-monthly basis. Trials involving the care group approach in LMICs improved breastfeeding and complementary feeding practices (Kushwaha *et al.*, 2014; Shakya *et al.*, 2017), and reduced underweight rates in children (Davis *et al.*, 2013). Delivering an integrated intervention using the care group model can provide the urgently needed cost-effective, scalable and sustainable intervention to prevent and reduce malnutrition among refugee children in the West Nile region of Uganda. Uganda is one of the countries in which care groups are being implemented on a small scaleby independent non-governmental organizations targeting community development (Perry et al., 2015), though the effects and sustainability of care groups remain untested in post-emergency settlements. Further, the care group approach is a strategy that the government of Uganda could adapt if enough evidence is provided of its success in improving child health and nutritional outcomes that can be replicated.

3. Participants:

a. Describe the study participants and the population from which they will be/were drawn.

Participant population: The mother, father, and child (<23 months) participants will be drawn from South Sudanese refugee populations living in post-emergency settlements in the West Nile region of Uganda. They are

MAKERERE UNIVERSITY
SCHOOL OF HEALTH SCIENCES
APPROVED
VALID [illegible]
01 MAY 2020
RESEARCH & ETHICS COMMITTEE
P. O. BOX 7072, KAMPALA

protected by the Government of Uganda (GoU) through the Office of the Prime Minister and the United Nations High Commission for Refugees (UNHCR). They are settled in designated regions which are composed of different villages with local administrative units and leaders with whom we will be in contact prior to and during the study.
Age range: Pregnant women of reproductive age (15 - 49 years) in their third trimester and their husbands are eligible to participate in the study. Participants will be enrolled after signing written consent.
Number of participants: 200 households

b. Describe any screening procedures and any inclusion or exclusion criteria.
Only mothers in their third trimester of pregnancy and their husbands will be eligible to participate in the study. Though, for one of the treatment arms, single mothers can be recruited because we test the effect of the participation of the mother in a care group. During the intervention, if the husband leaves or dies, the mother will remain in the care group until the end of the study, but we will not include the data collected from her in the data analysis portion. Similarly, fathers whose wives left or died would be treated the same way. Note: In the calculation of the sample size we allocated for 20% respondent loss in order to cover adverse effects of such incidences on the study outcomes.

c. Provide sample size and a clear justification as to how you arrived at your projected sample size.
With a small effect size of 0.15, a type I error of 0.05, a power of 0.90, 160 households are needed to detect differences in LAZ between the 4 arms. With an anticipated loss to follow up of 20%, we will enroll 200 households at baseline.

*Note: You do not need to provide an exact sample size and you should estimate a somewhat larger number than you think you will actually collect. You may collect data on fewer subjects than stated without further IRB review as long as you still meet the scientific aims of your study, but you may not collect data on **more** subjects without submitting an amendment.*

d. Describe whether identifiers will be collected.
Identifiers will be collected from all participants. These identifiers will include the name of the participant (which will be later be coded) and date of birth (participants and the children), signatures or thumbprints for consent.
Name: The names of the mother and child will be required as the first identifier on each questionnaire which will be matched against the household ID (Identification Number). The name of the mothers and fathers will be used in creating the care groups through which the intervention will be done. These lists will only be used by the principal investigators and co-principal investigators until data collection has ended and all questionnaires have been checked and coded. After such a time,the master list of names, will be disposed of.
Date of birth: Infant's date of birth will be used to determine their respective ages which will be used for anthropometric data analysis for example for calculating the length-for-age z-scores, weight-for-age z-scores and height for the mother. Maternal height will be used as covariates during data analysis.
Other identifiers: In obtaining consent for participation in the research, all participants will be asked to sign their names on the consent form but for illiterate participants, the researcher will provide an inkpad which will be used to obtain a thumbprint as consent for participation in the research.

4. Study procedures: (ANSWER EITHER a. OR b. UNLESS BOTH APPLY)

a. If your study involves analysis of existing data or specimens only:

1) Describe the source(s) of the data and whether it is publicly available or not.

Not applicable

2) If the PI or other investigators were involved in the original data collection, provide the IRB number.

Not applicable

3) Describe whether the original dataset included identifying information and whether the form of the dataset to be used in the new study includes identifiers or not.

Not applicable

4) Provide a brief data analysis plan.
Not applicable

MAKERERE UNIVERSITY
SCHOOL OF HEALTH SCIENCES
APPROVED
VALID [illegible]
01 MAY 2020
RESEARCH & ETHICS COMMITTEE
P. O. BOX 7072, KAMPALA

Note: Do not describe details of how the data were collected in a manner that would lead a reviewer to think that your study is currently having contact with human subjects.

b. If your study involves contact, direct or indirect, with subjects, provide the following:

1) General study design and methods.

This will be a cluster-randomized controlled trial in the West Nile Region post-emergency settlements in Uganda. The primary outcome will be child length-for-age z-score (LAZ) because of its stability and reliability. There is an increase of 35% in length between birth and 6 months and an increase of 13% from 6 to 12 months. Specifically, according to the WHO Growth Standards, a child should gain 17.8 cm during the first 6 months and 8.2 cm from 6 to 12 months. These rapid changes in length from birth to 9 months make any deviation from the optimum growth relatively easy to detect with the appropriate statistical power. The secondary outcomes include weight-for-age z-scores (WAZ) as well as breastfeeding and complementary feeding practices as defined by WHO (2010). With a small effect size of 0.15, a type I error of 0.05, a power of 0.90, 160 households are needed to detect differences in LAZ between the 4 arms. With an anticipated loss to follow up of 20%, we will enroll 200 households at baseline. Participants will be identified using a two-stage stratified random sampling method. A settlement within the West Nile region will be randomly chosen from which a zone will be selected. A total of 8 villages within that zone will also be selected and randomly assigned to one of the 4 arms. The villages will be chosen to be geographically distant enough to minimize the potential spillover effects between arms (i.e. avoiding shared markets). **The 8 villages will be randomized in the 4 arms: 1) mother-to-mother care groups only, 2) father-to-father care groups only, 3) mother and father both participate in respective care groups and 4) control group with no parental participation in a care group.**

The intervention will have five phases: 1) formative assessment, 2) baseline data collection, 3) midline data collection, 4) end line data collection, and 5) data analysis and dissemination. The social cognitive theory proposed for this study will test several contexts and variations among participants to explain cognitive, behavioral and environmental factors towards improving child nutrition, growth, and development.

2) Study procedures, including sequence and timing.

Phase 1: Formative assessment

In-depth qualitative assessments using focus group discussion and interviews will be conducted among mothers, fathers, and key informants such as CHEWs and village leaders. Barriers and enablers of appropriate child feeding practices, social support, adequate hygiene, and child stimulation will be identified during this phase. Information regarding attitudes and perceptions around maternal mental health and fathers' involvement in child care will also be gathered. Then, the specific messages of behavior change for each topic (child feeding, basic hygiene, social support, group dynamics, child stimulation, mental health, and fathers' involvement) to be discussed during the care group meetings will be chosen.

We will also engage the appropriate stakeholders including the Office of the Prime Minister, the District Health Office (DHO), the CHEWs, and village leaders during this phase. Their perspective and input will provide valuable information on how to increase the success rate and the sustainability of the intervention. They will also help identify participants for the focus groups and interviews.

Training of the CHEWs will follow immediately after the specific messages are identified and sample training materials are prepared. After specific messages have been identified from the focus groups, CHEWs will be trained, assessment tools will be developed, and pre-tested and data collectors trained. CHEWs will be consulted during the design of the training materials because of their experience working with mothers.

Phase 2: Baseline data collection

Pregnant women of reproductive age (15 - 49 years) in their third trimester and their husbands are eligible to participate in the study. Participants will be enrolled after signing a written consent. CHEWs will support in identification of mothers for recruitment in the study. The CHEWs, the lowermost administrative unit of Uganda's health system (Ministry of Health Uganda, 2013) link community members to the nearest health facility where they can get antenatal services. Each CHEW will be able to identify participants based on their visits for antenatal services at the nearest health facility structure and their knowledge of the households that they routinely

MAKERERE UNIVERSITY
SCHOOL OF HEALTH SCIENCES
APPROVED
VALID UNTIL
01 MAY 2020
RESEARCH & ETHICS COMMITTEE
P. O. BOX 7072, KAMPALA

monitor as they perform their roles. A respondent list will be generated from which participants within a similar neighborhood will be sought for consent to participate in the project.

A total of 10-15 neighboring women and/or men, depending on the treatment arm, will constitute a care group and will select a peer leader (lead mother or lead father). Once selected, all the peer leaders will be trained by their respective CHEWs regarding the specific messages for behavior change. Each peer leader(s) group will be assigned to a specific CHEW. Topics will include appropriate infant feeding practices, adequate basic hygiene practices, optimal child home stimulation, and mental health. They will also receive training regarding group dynamics and interpersonal skills. The peer group leaders will lead the care group meetings independently every two weeks. Topics will be similar but delivered separately among the groups. There will be more emphasis on fathers' involvement in child care in the fathers' care group. Strategies and approaches on how to address each topic to increase adherence will be identified during the formative phase. All the care groups leaders within each arm will meet with their CHEW to discuss issues and concerns with their groups but also share experiences and successful strategies. During this phase, socio-demographic information for each household will be collected. Household food insecurity will be assessed using the Household Food Insecurity Access Scale (Coates et al., 2007). The WHO Self Reporting Questionnaire (SRQ) (van der Westhuizen et al., 2016) will be used to assess mental health status. Social support will be assessed using the Duke Social Support Index (DSSI) (Broadhead, Gehlbach, De Gruy, & Kaplan, 1988; Powers, Goodger, & Byles, 2004). All these standardized questionnaires will be pre-tested during the formative phase with families who are not participating in the longitudinal study. Participants in the trial will be followed up for at least 9 months after birth.

Phase 3: Midline data collection

Birth outcomes data including birth weight and birth length will be confirmed by the research team within one to two weeks of birth. Mothers giving birth to premature infants or infants with congenital abnormalitieswill be retained in the study intervention but data from those households will not be included in our final analysis. Infant anthropometric data (length, weight) will also be measured at 3 months. Breastfeeding indicators including early initiation of breastfeeding (at 3 mo) and exclusive breastfeeding (at 3 and 6 mo) will be collected as well.

When the infant is 6 months, anthropometrics will be measured along with infant development using the Ages and Stages Questionnaire (Paul H. Brookes Publishing Co, 2018). Subscales of language, gross motor, and personal-social development will be assessed. Complementary feeding practices including the introduction of complementary foods, minimum dietary diversity, minimum meal frequency, minimum acceptable diet, and consumption of iron-rich foods will be collected. Maternal mental health status and social support will also be assessed at 3 and 6 months.

Phase 4: Endline data collection

At endline (9 mo), infant length, weight, and developmental measures will be assessed. Complementary feeding practices including the introduction of complementary foods, minimum dietary diversity, minimum meal frequency, minimum acceptable diet, and consumption of iron-rich foods also will be collected. Mental health status and social support will be assessed as well.

Tools for assessment of IYCF practices, household food security and WASH will be cross-referenced with the UNHCR standardized expanded nutrition survey (SENS) tools (UNHCR, 2019)

Phase 5: Data analysis and dissemination

After primary data analysis, preliminary results will be shared with the stakeholders, the Office of the Prime Minister, the local DHO, the CHEWs and the village leaders through presentations and reports. Manuscripts will also be developed and published in scientific journals after in-depth data analysis. In-depth results will also be presented at local conferences in Uganda and at international conferences for global nutrition.

Provisions for attrition in the study

Four of the researchers on this study (JK, MK, CW and HR) can work with existing leadership structures and individuals supporting the research, such as the CHEWs, in order to implement the interventions and conduct the data collection exercises. Furthermore, JK and MK are Ugandans who have previous experience working with grassroots support structuring in nutritional programming which gives an added advantage to the project.

MAKERERE UNIVERSITY
SCHOOL OF HEALTH SCIENCES
APPROVED
VALID UNTIL
01 MAY 2020
RESEARCH & ETHICS COMMITTEE
P. O. BOX 7072, KAMPALA

The treatment arms will each be assigned three community health extension workers (CHEWs). Each CHEW will supervise a maximum of two care groups. In the event that one of the CHEWs left, the two other CHEWs assigned to each treatment arm can cover the gap in terms of supervising the care groups while the PI recruits and trains another local CHEW to take on the role of supervising the care groups in the study.

Participants attrition, including mothers, infants, and fathers, was considered in the original experimental design of the study. The researchers allocated an additional 20% recruitment of individuals for the study in order to avoid major effects of participant dropout on outcomes of the study.

Regarding enumerators/ data collectors, a total of four enumerators will be trained and engaged in this study. The student researchers, JK, HR, and CW will provide overall supervision and lead in data collection to ensure that the quality of data is always kept high. The engagement of the student researchers in the data collection process increases the productivity of the data collection team and affords the team some cbackupin case a member left giving ample time to recruit and retrain.

Translators and transcribers will be engaged in pairs in order to provide a backup in case one of the members is unable to continue due to an emergency or unavoidable circumstance.

All six investigators undertaking this study in their varying roles have the proficiency to analyze, interpret and report findings from the study. Attrition at such a stage would be well covered through shifting of priorities within the entire research team.

Overall, training and refresher trainings will be sure information delivered is high quality. Participantwill be given a phone number to directly reach the PI to ensure that participants concerns are always heard. This feedback will allow researchers to resolve potential problems that might otherwise cause participants to drop out.

3) Number of study contacts or visits required of participants.

Care groups will be held bi-monthly for 12 months which add up to 24 care group meetings for each participant. Data will be collected four times throughout the study: baseline, 3 months, midline, and endline.

Activity Name:	Activity Description:	Participant Time Commitment:	Person Conducting Activity:	Safeguards:
**Recruitment (focus group discussions)**	All recruitment will be handled with the aid of the local leaders in order to have preserve civil engagement with the respondents and general knowledge about our activities within the area. We will engage the appropriate stakeholders including the Office of the Prime Minister, the DHO, the CHEWs, and village leaders	Less than 10 minutes for each respondent	Recruitment of the local leaders, community health extension workers (CHEWs) and key informants will be handled by the researcher Recruitment of the individuals from the community will be done by the CHEWs supervised by the researcher	All recruitment will be done by consent of the respondent and the respondents will be informed of their right to exit the interviews at any time during the project. Different activity recruitment forms have been drawn specific to the project activity, for example focus group discussion recruitment form. Confidentiality of the information provided especially personal information such as names, age will not be attributed to specific statements from the focus groups.

MAKERERE UNIVERSITY
SCHOOL OF HEALTH SCIENCES
APPROVED
VALID UNTIL
01 MAY 2020
RESEARCH & ETHICS COMMITTEE
P. O. BOX 7072, KAMPALA

**Consent request**	All respondents reserve the right to consent to participate in the project activities. The respondents will sit individually for interviewer questionnaires and in groups for focus group discussions. The consent will be read and each person will be required to affirm their participation by signature of their name or use of their thumb print (ink pad provided by the researcher).	Less than 10 minutes for each respondent	Researchers, data collectors and translators for the focus group discussions.	All signed consents will be securely stored and taken off as the first page before each interview.
**Recruitment of respondents for care groups intervention and control group.**	Participants will be identified using a two-stage stratified random sampling method. A settlement within the West Nile region will be randomly chosen from which a zone will be selected. A total of 8villages within that zone will also be selected and randomly assigned to one of the 4 arms. The villages will be chosen to be geographically distant to minimize the potential spillover effects between arms. The 8villages will be randomized in the 4 arms: 1) mother-to-mother care groups only, 2) father-to-father care groups only, 3) mother and father both participate in respective care groups, and 4) control group with no parental participation in a care group(current standard of care).<br>The formative part of	Less than 10 minutes for each respondent	Researcher, Community Health Extension Worker	All recruitment will be done atthe consent of the respondent and the respondents will be informed of their right to exit the interviews at any time of the project.

MAKERERE UNIVERSITY
SCHOOL OF HEALTH SCIENCES
APPROVED
VALID UNTIL
01 MAY 2020
RESEARCH & ETHICS COMMITTEE
P. O. BOX 7072, KAMPALA

	the study and intervention will be done infive phases: 1) formative assessment, 2) baseline data collection, 3) midline data collection, 4) end-line data collection, and 5) data analysis and dissemination.			
**Qualitative assessment: Focus Group Discussions**	Conduct 8 focus group discussions and interviews for mothers, fathers and other key informants such as Community Health Extension Workers and village leaders. Each group will consist of 8 individuals. These sessions will include discussion of barriers and enablers of appropriate child feeding practices, social support, adequate hygiene, and child stimulation. Information regarding attitudes and perceptions around maternal mental health and fathers' involvement in child care will also be gathered. Then, the specific messages of behavior change for each topic (infantfeeding, basic hygiene, social support, group dynamics, child stimulation, mental health management, and fathers' involvement) to be discussed during the care group meetings will be chosen	Each of the 8 focus group discussions will last 1 hour and 30 minutes.	Researcher and one translator will conduct the focus group discussions. Two transcribers will review and transcribe the audio recordings for the researcher to start the analysis.	All individuals will willingly participate in the interviews after signing a letter of informed consent. All information provided by the participants will be stored. The translators and transcribers will also sign letters of confidentiality before engaging in any part of focus group discussions

MAKERERE UNIVERSITY
SCHOOL OF HEALTH SCIENCES
APPROVED
VALID UNTIL
01 MAY 2020
RESEARCH & ETHICS COMMITTEE
P. O. BOX 7072, KAMPALA

**Caregroup meeting**	Bi-monthly peer-led meetings supervised by community health extension workers (CHEWs) and occasionally the principal investigator to effect the treatment of the study (integrated nutrition intervention)	30 minutes to 2 hours	Caregroup leaders (lead mother or lead fathers)	CHEWs and peer group leaders will have responsibility for selecting the bi-monthly dates most convenient forthe majority of the members based on . when they have reduced household tasks. Meeting will be held within the locality of their household. No transportation ramifications
**Quantitative data collection (Baseline, 3-month, midterm and end-line data collection)**	Quantitative data will be collected using a pre-tested structured questionnaire that will be administered by the researcher and data collectors (enumerators). The respondents will be required to provide consent for participation. Two questionnaires (mothers, fathers) and a list of questions (focus group discussion) will be used and all will be translated to Arabic which is the most commonly used local language among the refugee population. Socio demographic information will be collected. Household food insecurity will be measured using the Household Food Insecurity Access Scale (Coates et al., 2007). The WHO Self Reporting Questionnaire (SRQ) (van der Westhuizen et al., 2016) will be used to assess mental health	Each participant will be engaged for 1 hour and 30 minutes	Researcher and data collectors (enumerator)	All respondents will have consent got from them for participation and will be informed of their option to opt out of the interview process at any time. Respondents personal information such as names will be coded with numbers in order to mask the identity. All respondents' information will be stored in securely in order to preserve their confidentiality.

MAKERERE UNIVERSITY
SCHOOL OF HEALTH SCIENCES
APPROVED
VALID [illegible]
01 MAY 2020
RESEARCH & ETHICS COMMITTEE
P. O. BOX 7072, KAMPALA

	status. And social support will be assessed using the Duke Social Support Index (DSSI) (Broadhead, Gehlbach, De Gruy, & Kaplan, 1988; Powers, Goodger, & Byles, 2004). All these standardized questionnaires will be pre-tested during the formative phase.			
**Anthropometric data collection**	Birth outcomes data including birth weight and birth length as well as breastfeeding indicators will be collected within one – two weeks of birth. These will be collected from the index child's health cards and health centers. Infantanthropometric data (length, weight) will also be measured at 3 months. Breastfeeding indicators including early initiation of breastfeeding and exclusive breastfeeding will be collected as well.	Less than 30 minutes per child	Researcher	Consent for getting child anthropometrics and basic information will be got from the mothers or fathers of the children.

MAKERERE UNIVERSITY
SCHOOL OF HEALTH SCIENCES
APPROVED
VALID [illegible]
01 MAY 2020
RESEARCH & ETHICS COMMITTEE
P. O. BOX 7072, KAMPALA

	When the child is 6 months, child anthropometrics will be measured along with child development using the Ages and Stages questionnaire (Paul H. Brookes Publishing Co, 2018). Maternal mental health status and social support will also be assessed at 3 and 6 months. Complementary feeding practices including introduction of complementary foods, minimum dietary diversity, minimum meal frequency, minimum acceptable diet, and consumption of iron-rich foods will be collected at 9 mo.			All respondents' information will be stored in securely in order to preserve their confidentiality. Respondents personal information such as names will be coded with numbers in order to mask the identity.
**Data analysis and dissemination**	After primary data analysis, results will be shared with the stakeholders, the Office of the Prime Minister, the local DHO, the CHEWs and the village leaders through presentations and reports. Manuscripts will also be developed and will be published in scientific journals after in-depth data analysis. The results will also be presented at local conferences in Uganda and at international conferences for global nutrition	Presentations will last an hour.	Researcher and Co Principal investigators	Manuscripts and presentations will be approved by the researcher advisers guided by the university human subjects research guidelines

MAKERERE UNIVERSITY
SCHOOL OF HEALTH SCIENCES
APPROVED
VALID UNTIL
01 MAY 2020
RESEARCH & ETHICS COMMITTEE
P. O. BOX 7072, KAMPALA

4) Expected duration of the study.

The proposed study is 2 years (3 months-formative/ qualitative research, 12 months-intervention and data collection, 9 months-data analysis, reports, manuscripts and article writing).

5) A brief data analysis plan and description of the nature of the variables to be derived.

Qualitative data analysis: focus group discussions with mothers, fathers, CHEWs and village leaders will be audio-recorded, transcribed verbatim and back-translated into English. Thematic analysiswill be used by two independent researchers to identify common themes and to identify the specific behavior change messages to be delivered during the intervention. Results from the qualitative assessment will also inform the approaches and strategies to ensure that messages are culturally appropriate and context-specific.

Quantitative data analysis: repeated-measures ANOVA will be used to determine the differences in LAZ and WAZ between the 4 arms of the study. Z tests will be calculated to assess the difference between the IYCF indicators between the groups. For comparisons between the arms, continuous variables will be tested using ANOVA and categorical variables will be tested using chi-square tests. Linear and logistic regression methods will also be used to model the underlying and immediate factorsassociated with optimal child growth and development among refugees in the West Nile.

Address the items in the remainder of section 4b if they are relevant to your study

6) If human biospecimens (blood, urine, saliva, etc.) will be collected, provide details about collection, volume (ml) or number, use, storage, identification, and disposal. Include, if relevant, information about genetic or genomic analyses planned for the biospecimens.

Not applicable

7) Describe how subjects will be screened for eligibility and assigned to study/intervention and comparison/control groups.

Households to participate in study will be identified by the community health extension workers (CHEWs) together with the principal investigator. The CHEWs will generate a list from which caregroups will be developed based on geographic proximity. Pregnant women of reproductive age (15 - 49 years) in their third trimester and their husbands are eligible to participate in the study. Participants will be enrolled after signing written consent. Eligibility will be based on the inclusion criteria in respondent identification before the start of the intervention. Only mothers in the 3rd trimester of pregnancy and their husbands will be considered.

Randomization will be done at the experimental design stage; that is, a settlement within the West Nile region will be randomly chosen from which a zone will be selected. A total of 8 villages within that zone will also be selected and assigned to one of the 4 arms. The villages will be chosen to be geographically distant enough to minimize the potential spillover effects between arms (i.e. avoiding shared markets).

8) Explain and justify whether there will be blinding.

Participants in the study will be followed up for at least 9 months after birth. Care groups within a treatment arm together with their CHEWs will be blinded from the treatment given to care groups in other treatment arms. The CHEWs for each arm will be trained separately depending on their assigned treatment arm. This will be done to minimize spillover of the intervention between treatment arms. Data collectors and enumerators will also be blinded togroup assignment in order to prevent any form of bias in probing or making observations during data collection.

9) Explain and justify whether participants will not receive routine care or will have current therapy stopped.

No therapy is being stopped; rather, the care group intervention is being added to routine care.

10) Explain and justify the use of a placebo or non-treatment group.

The use of a non-treatment group is required by the randomized controlled design of the study. Therefore, a non-treatment group is needed to evaluate the effectiveness of the intervention on the study outcomes. Even though households in the non-treatment group will not receive training from this study, they will still receive the current standard-of-care practices administered by the local DHO and the non-treatment group will also be provided at least with the intervention printed training materials through their CHEWs.

11) provide a definition of treatment failure or participant removal criteria.

MAKERERE UNIVERSITY
SCHOOL OF HEALTH SCIENCES
APPROVED
VALID UNTIL
01 MAY 2020
RESEARCH & ETHICS COMMITTEE
P. O. BOX 7072, KAMPALA

If a child died during the study, then that household will be allowed to remain in the care group for the study duration for ethical reasons such as stigma reduction. However, the responses from that household will be excluded in the final analyses.. If a mother or father left the area after beginning the study, then previous data of that respondent will be excluded from the final data analyses. That said, single mothers or single fathers in a mothers' only or fathers' only caregroup will be rightly engaged in the study.
If a household/participant caught an infectious disease or in some way presents a risk to the greater population and is quarantined or taken away for a prolonged period by the authorities, then, results of that respondent will be excluded from the final data analyses.
Note: The researchers have made provisions for attrition (described earlier) within all personnel involved in this research to avoid adverse effects of participant withdrawal from the study

12) Describe what happens to participants receiving therapy when the study ends or if a subject's participation ends prematurely.

Because the study is based on a social behavioral change communication model, the researchers plan to involve local leaders from the district level to all key stakeholders. These include local- based Office of the Prime Minister personnel such as the area commandant and UNHCR personnel which would provide a case for adoption into the framework of their activities as a best practice/intervention if successful results justified by scientific evidence in preliminary reports are provided. The CHEWs, who are monitored by health officers from theDHO, will continue to provide routine support to the formed groups based on the preliminary reports shared with the DHO with justifiable evidence of improvement in child nutrition and development and maternal mental health status The researchers intend to apply for post-intervention grants with the Nestlé Foundation and other funding agencies that are being identified in order to sustain and expand the study's treatment to the wider community. The PI and one of the advisors and co-PI's (MK) are keen on lobbying with NGOs in the country to reach out to more people through this care group model based on lessons learned with this initial studys. The researchers are confident of proposing an evidence-based strategy to government through the Ministry of Health and other sectors such as the Office of the Prime Minister to inform policy for sustainability of the care group model intervention as a project.

13) Describe the process for referring subjects to care outside the study, if needed.

Referral of participants will systematicallyfollow the guidelines for specific events within post emergency settlements as stipulated by the Office of the Prime Minister (OPM), UNHCR and the local government. For example, if a mother or child is sick, they would be referred to the nearest health facility supported by their CHEW. In case the sickness is suspected to be infectious, the local authorities, the overall settlement authorities (OPM, UNHCR), the District authorities (political and technical heads) will be immediately alerted. The PI and his research team will also be responsible for a clearance protocol. The PI would prepare a brief incident report (using provided templates by IRB offices) that will be shared with the IRB managing institutions (MAKand OSU) and all co-PIs. Research study supervisors would also receive a copy of the report. If the respondent has a condition that poses a greater risk of harm to the general community, direct authorities will be informed in order to provide proper guidelines for resolving the issue.

14) For studies that evaluate interventions, have a randomized study design, and/or are a clinical trial, provide power calculations for projected sample size.

The primary outcome is infant LAZ. With a small effect size of 0.15, a type I error of 0.05, a power of 0.90, 160 households are needed to detect differences in LAZ between the 4 arms of the study. With an anticipated loss to follow up of 20%; we will enroll 200 households at baseline.

15) Describe any plan for reporting test results to participants. For medical tests, describe how results will be validated (e.g., conducted in a CLIA certified laboratory).

Not Applicable

5. Data Security and Protection of Subject Confidentiality (NOTE: LOSS OR THEFT OF COMPUTER OR HARD COPIES OF DATA COLLECTION SHEETS DURING TRANSPORT IS GREATEST THREAT TO SUBJECT CONFIDENTIALITY – BE SURE TO TRAIN YOUR STAFF ABOUT THIS PROBLEM.)

MAKERERE UNIVERSITY
SCHOOL OF HEALTH SCIENCES
APPROVED
VALID [illegible]
01 MAY 2020
RESEARCH & ETHICS COMMITTEE
P. O. BOX 7072, KAMPALA

Identify the data security plan below that best describes how you will minimize the risk of a breach of confidentiality by checking one of the boxes on the left side of this chart. If your study includes sequential phases that require different procedures, or does not fit these categories, explain in "Other".

Note: Identifiers include name, address, hospital record number, etc., and other indirect identifiers (e.g., date of birth) that, when combined with other variables, may make a subject identifiable. These categories reflect minimal standards; you may impose more stringent protections.

**Hard Copy of data collection form: Indicate your choice by typing an X in the appropriate box on the left:**	
✓	Hard copies of data collection materials <u>have identifiers</u> and are locked in a secure cabinet or room with limited access by specified individuals. COPIES WILL BE KEPT IN INVESTIGATOR'S POSSESSION DURING TRANSPORT. When possible, redacted (de-identified) versions of the data collection sheets will be used for coding and analysis.
✓	Hard copies of data collection materials include an ID code and <u>do not have personal identifiers.</u> However, a code linking the data to the subject's personal information is stored separately from the data collection sheets and is locked in a secure cabinet or room with limited access by authorized individuals. CODE WILL BE KEPT IN INVESTIGATOR'S POSSESSION DURING TRANSPORT.
✓	Data are not collected on paper.
✓	Other (describe): Data that is stored on Oklahoma State University Qualtrics cloud will first be encrypted and the password will only be privy to the investigators on this project. Data on hard disks will also be encrypted before storage.
**Electronic Databases: Indicate your choice by typing an X in the appropriate box on the left:** *Note: A de-identified version of the database should be used for data analysis except in instances in which identifying information is prerequisite for coding or analysis. Databases that retain identifying information require a higher degree of electronic security.*	
	The study is minimal risk and data collected are not sensitive in nature. <u>No personal identifiers</u> are included in the electronic database.
✓	<u>Personal identifiers</u> are included in the database. The data are stored on a computer that is password protected with a secure server. Transfer or storage on portable devices (e.g., laptops, flashdrives) is encrypted. The devices on which this information is stored are accessible only to individuals who need access to these data.<br>Personal identifiers may exist for qualitative data which will be securely stored after encryption. These identifiers will not be included in final reports)
✓	<u>No personal</u> identifiers are included in the database, but linkable identifiers exist separately, and the data are sensitive in nature *(e.g., substance use, mental health, genetic propensities, sexual practices or activities)* such that disclosure could provide a risk to the individual. The codes are stored on a computer that is password protected with a secure server. Transfer or storage on portable devices (e.g., laptops, flashdrives) is encrypted. The devices on which this information is stored are accessible only to individuals who need access to these data.
	Other (describe):

c. If you are using participants' personal identifiers, describe any plans for disposing of identifiers including if, when and how that will be done.

The master key that has the names of the participants, household ID and the matching codes consistent with the questionnaires will be encrypted and stored on a drive in a lockable file cabinet in a secure office. Another encrypted copy of the master key will be stored in cloud storage just in case the other copy was destroyed physically or by a computer virus. At the end of the study/project, the master keys will be permanently deleted off the drive and cloud space. The links on the master code will be removed at the end of the project. This will happen by Fall 2020.

Note: Data will be collected using tablets, there will be limited use of physical data. Any physical materials containing respondent information will be disposed of through the OSU Department of Nutritional Sciences administrative office.

MAKERERE UNIVERSITY
SCHOOL OF HEALTH SCIENCES
APPROVED
VALID UNTIL
01 MAY 2020
RESEARCH & ETHICS COMMITTEE
P. O. BOX 7072, KAMPALA

d. Describe any plans for destroying data including if, when and how that will be done.

Qualitative data: The audio recordings will be permanently deleted/ erased off the voice recorders after being encrypted and backed up on the OSU Qualtrics cloud. Transcribed sheets will be destroyed following Oklahoma State University (OSU) and the Department of Nutritional Sciences guidelines of used academic information disposal after the end of the study.

Quantitative data: All data sources (cloud and hard disk storage) will be disposed of using OSU data disposal guidelines with the support of the IT office in the OSU Nutritional Sciences Ddepartment. Any documents containing identifiers will be disposed of through the OSU Nutritional Sciences department administrative office

6. **Recruitment process:**

a. Describe how participants will be recruited.

After identification of the villages to conduct research within the zones where refugees are residing in post-emergency settlements, the researcher with the aid of the Community Health Extension Workers (CHEWs) will identify households with pregnant women in their 3rd trimester and their husbands. A list will be developed from which peer support groups (care groups) for mothers and fathers respectively will be formed. Each group will contain approximately 10 individuals living in similar geographic proximity forming 20 groups (200 households). The CHEWs are local health and nutrition focal persons in the community where they reside. They link health facilities to the grassroots people; these CHEWs would be able to easily identify mothers and their spouses from their routine community work.

b. Explain how your recruitment materials will be used.

Personal Contacts: The CHEWs together with the PI will identify mothers who are suspected to be in their 3rd trimester. Antenatal records will be used to make confirmations of period of pregnancy. Two midwives from the nearest health facility will also be engaged in supporting to provide confirmation of the accuracy of the pregnancy duration for better selection of participants into the study.

Snowball Methods: The midwives, mothers and CHEWs will also utilize the snowball method in order to identify the next household from which participants could be added on the potential respondent list before creation of the care groups.

c. If relevant, address any privacy concerns associated with the recruitment process.

A standard recruitment consent form has been developed by the researchers which will provide potential respondents an option to decline or agree to participate when the PI and CHEWs explain what the study will be about and its duration.

Medical information such as antenatal records will only be used for verification of pregnancy trimester for inclusion which will be done by either the midwives or PI.

7. **Consent process and documentation:**

a. Describe who will obtain informed consent from participants, and how, when and where consent will be obtained.

The PI and the enumerators collecting data in the study will obtain consent from the participants at the beginning of the study and continuously remind them of their acceptance to participate or prerogative to opt out during further stages of more data collection.

All adults who wish to be involved in this study must give their consent for participation. Consent for infant's participation will also be sought from the parent.

Adult consent: The researcher or data collector will introduce the study by thetitle and stipulate the names of the investigators. The interviewer will describe the participant's role in the research process. The interviewer will then relay the purpose of collecting the information, for example, this study will collect information about the role of peer support groups, child development, and maternal mental health on child feeding practices and nutrition status in post-emergency settlements in the West Nile region in Uganda. The interviewer will then explain all activities that will be involved in the study, for example, care group meetings, measurements, observations and questionnaire interviews as well as the time commitment required and the way the information will be recorded.

Risks involved: The interviewer will then mention the risk of participation to the subject. For this proposed study there are no risks associated with the project which are greater than those ordinarily found in daily life or in usual measurements of an infant at the health center.For example, the researcher may expect that a child beingweighed, and length taken may be stressed probably because of the nature of

MAKERERE UNIVERSITY
SCHOOL OF HEALTH SCIENCES
APPROVED
VALID UNTIL
01 MAY 2020
RESEARCH & ETHICS COMMITTEE
P. O. BOX 7072, KAMPALA

equipment or perception that they may have pain inflicted on them. The researcher anticipatessolving this through working with parents of child guardians during through the data collection session. Similar risks are expected but will be resolved provisions of the MAKSHSREC IRB provisions of cooperative participation of respondents, compensation, immediate incident reporting to mention but a few.
Confidentiality: The interviewer will then explain the confidentiality of the information and identity of the participants.
Compensation: The interviewer will then explain the compensation that will be provided to the participants. First, individuals engaged in the focus group discussions will receive a soft beverage and a snack during the session.Additionally, caregroup leaders' monthly meetings with the community health extension worker will be facilitated with a soft drink and a snack.
Additionally, compensation in terms of at least onehousehold item (small cup and spoon for child feeding, cooking oil, packet of iodized salt, nail cutter, bar of washing soap)worth approximately $1will be given to each participantat each data collection session. Total compensation for all beneficiaries through the study will costapproximately $1,000.
Contacts: The participants will then be provided with the contact of the principal investigator in case they have any inquiry about the study.Additionally, the participants will be provided with the phone, e-mail and mail address of MAKSHSRECIRB as another contact in case they have queries or questions about the study or their rights regarding their participation in the study.
Participants rights: The participants will be informed of their rights to provide information voluntarily and that there will be no reprimand for refusal to participate. The participants will also be informed of their right to withdraw from the interview or the study at any time..
Confirmation of agreement: The participants will be asked to provide confirmation that they understood the information that had been relayed to them in the consent and that they are willing to participate. The participant will confirm with their signature or fingerprint followed by the interviewer's signature and date.
Infants will also be part of the participants in this study. The involvement of children in the study is to acquire anthropometric data to assess their nutritional status. The consent for infant participation is includedin the mother's consent form interview questionnaire of the mother If a mother does not want theinfant to participate, then her own information would not be required for the study. The mother will give her own consent and that of her child as a dyad.

b. If the study will involve vulnerable populations (e.g., children, prisoners, cognitively impaired adults, non-English-speakers, etc.) describe efforts to ensure their understanding of the research and the extra protections that will be in place to ensure their voluntary participation.

The researcher will use the Collaborative Institutional Training Initiative (CITI) guidelines on "research involving pregnant women, fetus, and neonates" and "research with children". Because the children proposed for this study will be physically unable (0-12months) to provide consent for inclusion in the study, consenting for participation will be given by their parent(s). At the first point of the study (baseline), the researcher will seek consent for participation in the study by reading out each aspect of the consent statement to each participant; for otherdata collectionperiods, that is 1-2 weeksafterbirth, at 3 months, 6 months andat 9 monthsthe parent(s) will be reminded that they already consented to the whole study but that participation is voluntary and they can always drop out for any reason.. In the general participant consent, the researchers included a description of the need to take anthropometric measurements of the infants and the requirement for consent by the parent(s) to conduct that activity during the data collection process. Mother and infant data is required as a pair, that means that if a mother fails to consent for a child's measurements to be taken, then that mother's data will automatically be excluded from the final data analysis sets. Due to the importance of retaining participants in the study over the entire intervention period, the researchers will emphasize the objectives of the study and the commitment expected over the project duration at the time the study is initially explained. This is a key factor in the participant recruitment process at the start of the study.

c. If a waiver of consent or a waiver or alteration of signed consent is requested, provide a justification for the waiver/alteration, and describe any alternate procedures for informing participants about the research.

MAKERERE UNIVERSITY
SCHOOL OF HEALTH SCIENCES
APPROVED
VALID UNTIL
01 MAY 2020
RESEARCH & ETHICS COMMITTEE
P. O. BOX 7072, KAMPALA

Not applicable

8. **Risks:**

a. Describe the risks associated with the study and its procedures, including physical, psychological, emotional, social, legal, or economic risks.

There are no risks associated with this study/project which are greater than those ordinarily found in daily life, for example, the researcher may expect that a child being weighed, and length taken may be stressed probably because of the nature of equipment or perception that they may have pain inflicted on them.

b. Describe steps to be taken to minimize those risks.

The researchers including the data enumerators will participate in training and refresher training on research ethics. The research team will take additional care on being gentle especially with anthropometrics measurements and the mother will be asked to assist so that the infant will be less frightened. The research team will also review and work towards security protocols provided by local authorities

c. Describe the research burden for participants, including time, inconvenience, out of pocket costs, etc.

The main potential research burden for the participants is time. Care groups will be held bi-monthly for 12 months for the participants to attend. Furthermore, they will be participating in data collection at four different time points throughout the study. They will receive a beverage and snack at each care group meeting and should incur no out-of-pocket costs because the care groups will be held within walking distance in their local community. Furthermore, each care group will decide on the best day and time for them to meet, thereby decreasing potential opportunity cost losses.

d. Describe how participant privacy will be protected during data collection if sensitive questions are included in interviews.

The researchers will be conducting individual interviews in an open area but away from other persons not engaged in the study, for example under a tree shade in the compound. All respondent's information will be coded after collection as part of providing privacy for the information.

9. **Benefits:**

a. Describe any potential direct benefits to participants from participating in the research (not including payment for participation).

The participants, both mothers, and fathers, will benefit from the bi-monthly group discussions with their peers regarding child feeding, basic sanitation, child home stimulation, and stress management. Since the intervention is based on social behavioral change communication, the participants will enhance their understanding of these topics and their relation to child care. The participants in the intervention groups are expected to have healthier children and those in the control group will benefit from the post-intervention grant involvement.

b. Describe potential societal benefits likely to derive from the research.

In the long-term, there is a potential to reach more parents beyond the project participants as enrolled mothers and fathers can increase the reach to future mothers and fathers. The participants will also be trained on basic skills for assessing their children for malnutrition during the weekly group meetings which will allow them to help teach other parents how to assess their children for malnutrition. The overall results of the project design are expected to bridge the gap between government nutrition officials and the local community. Overall, the social networks created during the intervention will continue to provide support for mothers and fathers who are enrolled in the study but also for future parents with updated and regular training of the Community Health Extension Workers (CHEWs) and the peer leaders from the DHO-nutrition. The findings will also be shared within different academic, socio-behavioral change developmental platforms and peer-reviewed journals to build on the existing knowledge bank on child health and nutrition among displaced persons especially in Low- and Middle-Income Countries (LMICs) and provide areas for further study or projects. The findings will be also be shared with the humanitarian administrators and stakeholders to support provide learning lessons for best practices for adoption and poor

MAKERERE UNIVERSITY
SCHOOL OF HEALTH SCIENCES
APPROVED
VALID UNTIL
01 MAY 2020
RESEARCH & ETHICS COMMITTEE
P. O. BOX 7072, KAMPALA

practices for improvement on nutrition intervention strategies. Furthermore, the project findings will be used to complete the requirements of students' dissertation at OSU and form manuscripts for publication in different journals for both the qualitative and quantitative findings.

10. Payment:

a. Describe the form, amount, and schedule of payment to participants.

No financial benefits will be given to any participants in this study. Participant in focus group discussions will be offered a snack and soft beverage during the interview sessions. Further, caregroup members also will be offered a snack and soft beverage at the last monthly meeting organized by their caregroup leaders. Additionally, to compensate participants for their time, at least one household item (small cup and spoon for child feeding, cooking oil, packet of iodized salt, nail cutter or bar of washing soap) worth $1 will be given to each participant at each data collection session. Compensation for all beneficiaries through the study will cost $1,000.A total of $1,648.6 will be used in the procurement of these compensatory items and items to be used during the study such as snacks and soft beverage for monthly group meetings for the participants over the course of the study.

b. Include the possible total remuneration and any consequences for not completing all phases of the research.

No financial remuneration will be offered for this study. The snack and soft beverage offered to the participants as per the scheduled meetings will be consistent with the study plan. Additionally, to compensate participants for their time, at least one household item (small cup and spoon for child feeding, cooking oil, packet of iodized salt, nail cutter, bar of washing soap) worth $1 will be given to each participant at each data collection session. Compensation for all beneficiaries' items through the study will cost $1,000.The distribution of these items will be to each beneficiary during each data collection session, therefore, there are no perceived consequenceregarding the items offered to the participants in case all phases of the research were not completedbecause items will be offered consistent with service (engagement in study) by the participants who will give of their time for data collection.

11. Safety monitoring:

a. Describe how participant safety will be monitored, by whom, and how often.

Participant safety will be under the overall control and guidelines of the local security authorities. That said, the researcher will ensure that enumerators and all individuals involved in research such as the CHEWs will be knowledgeable about ethics of research conduct including security protocols such as making immediate reports through the local security structures and the principal investigator. The PI will also instate a participant's feedback mechanism (PFM) as a 24-hourmobile phone held by the PI that can be called anytime by any participant to report an issue related to the study or a security issue that can be forwarded to the security authorities.

The research is based on working with different leadership structures and peer to peer accountability in terms of providing one another feedback on agreed goals towards improved nutritional practices. These same structures such as the CHEWs, the peer group leaders (lead mothers, lead fathers) will provide the routine monitoring of participants at each level.

b. If a Data Safety Monitoring Board (DSMB), or equivalent. will be established, describe the following:

i. The DSMB membership, affiliation and expertise.

A DSMB will not be utilized for this study because the intervention is based only on behavior change communication.

ii. The charge or charter to the DSMB.

Not applicable

iii. Plans for providing DSMB reports to the IRB.

Not applicable

c. Describe plans for interim analysis and stopping rules.

Not applicable

12. Plan for reporting unanticipated problems/adverse events:

MAKERERE UNIVERSITY
SCHOOL OF HEALTH SCIENCES
APPROVED
VALID [illegible]
01 MAY 2020
RESEARCH & ETHICS COMMITTEE
P. O. BOX 7072, KAMPALA

Describe plan for reporting to the IRB and (if applicable) to the sponsor unanticipated problems/ adverse events.

Unanticipated challenges or adverse events will be reported to School of Health Sciences Research and Ethics Committee (MakSHS-REC) and the OSU IRB office within 24 hours.

13. Other IRBs/Ethics Review Boards:

If the research was reviewed by other IRBs, provide the approval letter from the IRB which approved the research.

OSU IRB was approved.

14. Outside collaborations:

For studies that involve collaboration, describe the collaboration and the roles of each collaborator
This project is a collaborative effort between Oklahoma State University (OSU) and Makerere University Kampala (MAK).

JK (OSU) will manage ongoing relationships with stakeholders and local leaders. He will continue to contribute to the design of the study, lead the training of CHEWs, lead in initiating the data collection process, conduct data analyses and assist in report and manuscript writing. He will also ensure that IRB approval documents are up to date and are within the guidelines.

CW (OSU) will continue to contribute to study design and developing questionnaires and focus group questions. She will assist in conducting data analyses and lead the writing and dissemination of results.

HR (OSU) will continue to contribute to the design of the study, assist with questionnaire development, assist in the initiation of data collection, conduct data analyses and assist in report and manuscript writing.

BJS (OSU) will assist in the planning and the implementation stages of the project. She will also provide guidance on data analyses and report and manuscript writing.

DH (OSU) will assist in developing effective strategies to engage stakeholders prior to the intervention. She will also assist in the planning of the formative research and provide guidance on effective monitoring and evaluation.

MK (MAK) will provide local insight, guidance, and mentorship throughout the duration of the project. Dr. Kabahenda will also support by reviewing the results and reviewing and editing the manuscripts and reports prior to publication. There may be occasions where MK presents reports for the team to key Ugandan officials.

15. Oversight plan for student studies:

For student-initiated studies, explain how the PI will monitor the student's adherence to the IRB-approved research plan, such as communication frequency and form, training, reporting requirements, anticipated time frame for the research, and who will have direct oversight of the student if the study site is not local.

The PI, Joel J. Komakech, is a doctoral student who is under the guidance of Dr. Deana Hildebrand, Dr. Barbara J. Stoecker, and Dr. Margaret Kabahenda. They will all collaboratively ensure that JK adheres to the IRB-approved research plan by utilizing weekly communication through email and they also will visit the research sites when possible. They will also ensure that other students involved, CW and HR, have completed all necessary training, are following reporting requirements, and are making sure the research project is completed within a reasonable time frame. Because the study site is not local to DH and BJS, oversight of the PI will be MK, who resides in Uganda and who will be available for frequent telephone and email communications as well as making visits to the research location.

16. Oversight plan for study:

MAKERERE UNIVERSITY
SCHOOL OF HEALTH SCIENCES
APPROVED
VALID UNTIL
01 MAY 2020
RESEARCH & ETHICS COMMITTEE
P. O. BOX 7072, KAMPALA

Explain how the study will be managed, the qualifications of study personnel managing the project, and how personnel involved with the data collection and analysis will be trained in human subjects' research protections. If the PI will not personally be on-site during the data collection process, provide details about the supervision of the data collection and the communication plan between the PI and study team to assure adherence to the IRB-approved research plan.

JK, CW, and HR will be the primary persons responsible for the study. DH, BJS, and MK will all advise and provide technical assistance during the project. JK, CW, and HR are doctoral students and have experience conducting human research. DH, BJS, and MK are faculty members at OSU and MK and have extensive experience in nutrition research. (Brief CV's of all investigators have been attached)

Data collectors, enumerators, and translators will all receive training provided by the PI on ethical principles before the start of the study.

The PI and other co-investigators will be on-site during each of the data collection sessions. The PI and the co-investigators on-site will supervise data collection sessions to assure adherence to the IRB-approved research plan.

17. Data Coordinating Center: Note: Complete section 14 for each participating site.

a. How will the study procedures be developed? Who is responsible for considering the feasibility of the study intervention, and the risks the intervention poses to subjects?
   All researchers led by JK are responsible for study procedures development and the feasibility of the study intervention. Risk assessments will also be conducted by all investigators.

b. How will the study documents that require IRB approval at each local site be developed? Will there be some sort of steering or equivalent committee that will provide central review and approval of study documents, or will template consent forms, recruitment materials, data collection forms, etc. be developed by and provided to the local sites by the coordinating center without external review?

All study documents will be approved by the OSU IRB office and the School of Health Sciences Research and Ethics Committee (MakSHS-REC) at Makererebefore the start of the study. The PI will ensure that all study documents and approvals are up to date.

c. Will each local clinical site have its own IRB, and will the institution have an FWA with the federal government? State whether the coordinating center will collect IRB approvals and renewals from the clinical centers or not; if not, explain why not.

Not Applicable for this behavior change communication intervention.

d. How will the coordinating center provide each local site with the most recent version of the protocol and other study documents? What will be the process for requesting that these updates be approved by local clinical center IRBs?

All approved protocols will be distributed to the local sites. In the event of an approved protocol change, these documents will be distributed either via email or in-person.

e. What is the plan for collecting data, managing the data, and protecting the data at the coordinating center?
   Data will be collected using electronic tablets. All responses will be automatically saved on the OSU Qualtrics server for OSU. Only the investigators will have access to the data.

f. What is the process for reporting and evaluating protocol events and deviations from the local sites? Who has overall responsibility for overseeing subject safety: the investigators at the recruitment site, Coordinating Center, the Steering Committee, or a data and safety monitoring board (DSMB)? Is there a DSMB that will evaluate these reports and provide summaries of safety information to all the reviewing IRBs, including the coordinating center IRB? Please note that if there is a DSMB for the overall study, then the coordinating center PI does not have to report to the coordinating center IRB each individual adverse event/problem event that is submitted by the local site PIs.

There will be no DSMB at the local site.The PI will takeall responsibilityfor reporting and ensuring protocol events and deviations from the local site are promptly reported to the IRB offices and approved amendments effected at the local sites.

g. Who is responsible for compliance with the study protocol and procedures and how will the compliance of the local sites be monitored and reviewed? How will issues with compliance be remedied, if there are issues?

MAKERERE UNIVERSITY
SCHOOL OF HEALTH SCIENCES
APPROVED
VALID UNTIL
01 MAY 2020
RESEARCH & ETHICS COMMITTEE
P. O. BOX 7072, KAMPALA

The PI is responsible for the compliance with the study protocol and procedure. Dr. Margaret Kabahenda (MK) will provide in-country study supervision with routine communication and two scheduled field visits. Additional visits will be conducted based on increased funds for the study from other sources. The PI will also have bi-weekly skype meetings with the advisers at the OSU campus at the preliminary stages of the study which will be reduced to monthly meetings as the study goes on. These meetings will also be supported with completed phase briefs to all study advisers (MK, DH, BJS).
If there were any issues of compliance, these will be evaluated through the IRB offices (MAK& OSU) and the research advisers (MK, DH and BJS).

MAKERERE UNIVERSITY
SCHOOL OF HEALTH SCIENCES
APPROVED
VALID UNTIL
01 MAY 2020
RESEARCH & ETHICS COMMITTEE
P. O. BOX 7072, KAMPALA

18. Areas of Uganda in which research will be carried out:

District	County/ Municipality	Sub County/ Town Council	Parish	Duration
Adjumani	Dzaipi	Dzaipi	Arinyapi	12 months
Adjumani	Dzaipi	Dzaipi	Pagirinya	12 months

19. Name and address of organization recommending/sponsoring the candidate (P.O. Box Number, Telephone Numbers, street/Plot number, city/town)..

Nestlé Foundation - Place de la Gare 4 - PO Box 581 - CH-1001 Lausanne, Switzerland
Phone: +41 21 320 33 51 | Fax: +41 21 320 33 92
(Grant funding proposal resubmitted after initial reviewer comments and suggestions)

20. Project duration: 12 months

Signature of Researcher..

Date......7/9/2019......

MAKERERE UNIVERSITY
SCHOOL OF HEALTH SCIENCES
APPROVED
VALID UNTIL
01 MAY 2020
RESEARCH & ETHICS COMMITTEE
P. O. BOX 7072, KAMPALA

MAKERERE UNIVERSITY
SCHOOL OF HEALTH SCIENCES
AMENDMENT APPROVED
FOR OFFICIAL USE ONLY
10 JUL 2019
INSTITUTIONAL REVIEW BOARD
P.O. BOX 7072, KAMPALA

23. I undertake to submit:

i. Annual progress reports of my research project

ii. Final report on completion of the research project

iii. Copies of any publications arising from the research project

24. I hereby certify that to the best of my knowledge and belief, the particulars given in this form are true and complete in all respects.

Date....7/9/2019.................... **Signature of Applicant**...

MAKERERE UNIVERSITY
SCHOOL OF HEALTH SCIENCES
APPROVED
VALID [illegible]
01 MAY 2020
RESEARCH & ETHICS COMMITTEE
P. O. BOX 7072, KAMPALA

MAKERERE UNIVERSITY
SCHOOL OF HEALTH SCIENCES
AMENDMENT APPROVED
10 JUL 2019
INSTITUTIONAL REVIEW BOARD
P.O. BOX 7072, KAMPALA

23. I undertake to submit:

i. Annual progress reports of my research project

ii. Final report on completion of the research project

iii. Copies of any publications arising from the research project

24. I hereby certify that to the best of my knowledge and belief, the particulars given in this form are true and complete in all respects.

Date 7/9/2019 **Signature of Applicant** [illegible]

MAKERERE UNIVERSITY
SCHOOL OF HEALTH SCIENCES
APPROVED
VALID UNTIL
01 MAY 2020
RESEARCH & ETHICS COMMITTEE
P. O. BOX 7072, KAMPALA

MAKERERE UNIVERSITY
SCHOOL OF HEALTH SCIENCES
AMENDMENT APPROVED
10 JUL 2019
INSTITUTIONAL REVIEW BOARD
P.O. BOX 7072, KAMPALA

23. I undertake to submit:

i. Annual progress reports of my research project

ii. Final report on completion of the research project

iii. Copies of any publications arising from the research project

24. I hereby certify that to the best of my knowledge and belief, the particulars given in this form are true and complete in all respects.

Date....7/9/2019.................... **Signature of Applicant**..... ....

MAKERERE UNIVERSITY
SCHOOL OF HEALTH SCIENCES
APPROVED
VALID UNTIL
01 MAY 2020
RESEARCH & ETHICS COMMITTEE
P. O. BOX 7072, KAMPALA

MAKERERE UNIVERSITY
SCHOOL OF HEALTH SCIENCES
AMENDMENT APPROVED
10 JUL 2019
INSTITUTIONAL REVIEW BOARD
P.O. BOX 7072, KAMPALA

MAKERERE UNIVERSITY SCHOOL OF HEALTH SCIENCES RESEARCH & ETHICS COMMITTEE (Mak SHSREC)

Informed consent form for individual interviews

Peer groups to improve feeding practices and child nutrition in post-emergency settlements in Uganda

Investigators:

Joel J. Komakech, Oklahoma State University, joel.komakech@okstate.edu
Christine Walters, Oklahoma State University, christine.n.walters@okstate.edu
Hasina Rakotomanana, Oklahoma State University, hasina.rakotomanana@okstate.edu
Dr. Deana Hildebrand, Oklahoma State University, deana.hildebrand@okstate.edu
Dr. Barbara Stoecker, Oklahoma State University, Barbara.Stoecker@okstate.edu
Dr. Margaret Kabahenda, Makerere University Kampala, mkabahenda@caes.mak.ac.ug

Study sponsor:
The principal investigator (PI) has submitted a research funding proposal to The Nestle Foundation of $20,000 still under review. The PI will also seek internal funding opportunity within the university through small grants for students to conduct research in order to cover operational costs in conducting the study.

Background and rationale for the study:
Emergency aid is lacking when refugees move into post-emergency settlements making children more likely to be undernourished. In the West Nile post-emergency settlements, high rates of global acute malnutrition are seen. Effective and sustainable programs are needed to address the causes of child malnutrition. A social support intervention addressing basic hygiene, child feeding, and child development practices, and mental health is expected to be effective and sustainable in reducing child malnutrition among refugee children in the West Nile post-emergency settlements to prevent the long-term negative consequences of child undernutrition.

Purpose:
The purpose of this study is to test the effectiveness of an integrated nutrition intervention delivered through care groups in improving infant and young child feeding practices, child growth and development and maternal mental health. Your responses and measurements will help the researchers make recommendations on using the care group model in delivering integrated nutrition programs in the future.

Procedures:

- We will ask you questions about your household; and questions like how, when, and what you feed your infant. We will also ask you questions about hygiene, child play, maternal peer support, father involvement through social support and maternal mental health. The interview will take about 1 hour and 30 minutes.
- We will measure your infant's length and weight.

MAKERERE UNIVERSITY
SCHOOL OF HEALTH SCIENCES
APPROVED
VALID UNTIL
01 MAY 2020
RESEARCH & ETHICS COMMITTEE
P. O. BOX 7072, KAMPALA

NOTE: Do not sign this consent form if it does not have an IRB approval stamp, or if the date has lapsed

- We will also assess his or her development soon after birth, and at the age of 3 months, 6 months, 9 and 12 months.

Who will participate in the study?
We will enroll 200 households with pregnant women in their third trimester. In 50 of these households, a father or partner (for one treatment) will be in the study. You are expected to be active in the study for one year.

Risks/Discomforts:
There are no risks associated with this project which are greater than those ordinarily found in daily life. For example, the researcher expects that a child being weighed, and length taken may be stressed probably because of the nature of equipment or perception that they may have pain inflicted on them.

Benefits:
You will benefit from the twice-monthly group discussions with your friends regarding child feeding, basic sanitation, child home stimulation, and stress management. You will learn how these topics relate to child care.

You will also be trained on basic skills for assessing your child for malnutrition during the bi-weekly group meetings. You also will learn how to teach other parents to assess their children for malnutrition. The results of the project are expected to help communication between government nutrition officials and your community. The social networks created during the project can continue to provide support everyone enrolled in the study but also for future parents with updated and regular training of the Community Health Extension Workers and the peer leaders. from the district health officer-nutrition. The findings will also be shared with the Non-Government Organizations (NGOs) administrators and stakeholders to provide lessons for best practices for adoption and poor practices for improvement on nutrition interventions.

Alternatives:
Participation is completely voluntary and there is no penalty if you do not want to participate in the study. If you choose not to participate in the study, you would still be able to benefit from the results of the study based on lessons learned from the project. Furthermore, the researchers intend to apply for a follow-up grant which would reach out to more households in post-emergency settlements.

Cost:
There is no cost to you for participation in the study except your time. Costs of materials for the study will be covered by the grant funders whose interest is only in developing better ways to fight child malnutrition.

Compensation for participation in the study:
Financial compensation will be given to you for being in this study. We will provide you with approximately $2 (UGX 7,200) during each data collection session to appreciate you for your time.

MAKERERE UNIVERSITY
SCHOOL OF HEALTH SCIENCES
APPROVED
VALID [illegible]
01 MAY 2020
RESEARCH & ETHICS COMMITTEE
P. O. BOX 7072, KAMPALA

NOTE: Do not sign this consent form if it does not have an IRB approval stamp, or if the date has lapsed

There are no risks associated with this project which are greater than those ordinarily found in daily life, therefore, no injury is expected for any participant.

If you were somehow injured during your participation, first aid will be provided (as deemed necessary) and then you would be transported to the nearest health facility and someone in your family will be notified. The researchers will also prepare an incident report to be shared with the local authorities in the settlement and the district health office and with the offices for the protection of participants in research studies at Makerere University and Oklahoma State University.

Reimbursement:

All assessments and data collection will be performed at your residence or nearby in the community. The researchers will be sure that you can participate in a convenient area. Caregroup meetings will be held at a site chosen by your care group guided by the care group leader. The meeting area will be close to your household.

Questions:

If you have any questions you can reach the researchers in two ways: 1) There will be a 24-hour mobile phone held by the PI that can be called at any time that you have questions or concern, or 2) You may contact the Chairperson, School of Health Sciences Institutional Review Board (MakSHS-IRB) on (+256) 772-404970 / (+256) 0200903786 / or Uganda National Council of Sciences and Technology. Tel: (+256)-41-4705500.

Questions about participants rights:

If you have a question about your rights as a research participant, you may contact the Chairperson, School of Health Sciences Institutional Review Board (MakSHS-IRB) on (+256) 772-404970 / (+256) 0200903786 / or Uganda National Council of Sciences and Technology. Tel: (+256)-41-4705500

Research involving the collection of human materials/ samples:

Not applicable

Feedback on the study findings and progress of the study:

Regarding the progress of the study, the PI will participate in the routine stakeholders' meetings that are convened by the settlement leadership in order to give a brief account of the progress of the of the study and preliminary findings where necessary. The PI will also hold final stakeholders meeting in which a presentation of results will be provided regarding the main objectives of the study. The PI will also provide period reports through the district nutrition coordination committee meetings with the district health officer (DHO) on the general status of the study. The progress findings will be shared through the health system decentralized structure to the CHEWs who will provide direct feedback to the beneficiaries.

Also, the PI will also instate a participant's feedback mechanism (PFM) as a 24-hour mobile phone held by the PI that can be called anytime by any participant to report an issue related to the study or a security issue that can be forwarded to the security authorities. This system may also be used for inquiries on the progress of the study

Statement of voluntariness:

MAKERERE UNIVERSITY
SCHOOL OF HEALTH SCIENCES
APPROVED
VALID [illegible]
01 MAY 2020
RESEARCH & ETHICS COMMITTEE
P. O. BOX 7072, KAMPALA

NOTE: Do not sign this consent form if it does not have an IRB approval stamp, or if the date has lapsed

Your participation is completely voluntary and there is no penalty if you refuse to enroll in the study. You are also free to withdraw your consent and participation in this project at any time without any penalty

Approval of the research study:
This study has been approved by the Makerere University School of Health Sciences Research and Ethics Committee/IRB

Confidentiality:

The results of individual participants in this study will be kept strictly confidential and used only for research purposes. My identity will be concealed in as far as the law allows. My name and the name of my infant will be removed from all documents and replaced with a code number. Paper and computer records will be kept under lock and key and with password protection respectively.

The researcher/interviewer has discussed this information with me and offered to answer my questions. For any further questions, I may contact the Chairperson, School of Health Sciences Institutional Review Board (MakSHS-IRB) on (+256) 772-404970 / (+256) 0200903786 / or Uganda National Council of Sciences and Technology. Tel: (+256)-41-4705500

STATEMENT OF CONSENT

.... has described to me what is going to be done, the risks, the benefits involved and my rights regarding this study. I understand that my decision to participate in this study will not alter my usual health or medical care. In the use of this information, my identity will be concealed. I am aware that I may withdraw at any time. I understand that by signing this form, I do not waive any of my legal rights but merely indicate that I have been informed about the research study in which I am voluntarily agreeing to participate. A copy of this form will be provided to me.

Name.................. Signature/thumbprint of participant .... Age...............

Date (DD/MM/YY) ....

Name of Witness....................................... Signature of Witness.............................

Date (DD/MM/YY) ....

Name.............................Signature/thumbprint of parent or guardian for minors ....

Date (DD/MM/YY) ....

Name.. Signature of Interviewer ....

Date (DD/MM/YY) ....

MAKERERE UNIVERSITY
SCHOOL OF HEALTH SCIENCES
APPROVED
VALID UNTIL
01 MAY 2020
RESEARCH & ETHICS COMMITTEE
P. O. BOX 7072, KAMPALA

NOTE: Do not sign this consent form if it does not have an IRB approval stamp, or if the date has lapsed

Extra

(Mak SHSREC) ماكيريري كليه العلوم الصحية التابعة للجنة البحوث والأخلاقيات

مجموعات النظراء لتحسين ممارسات التغذية وتغذيه الأطفال في المستوطنات التي تلي حالات الطوارئ في أوغندا

:المحققين

j. Komakech، جامعه ولاية أوكلاهوما، joel.komakech@okstate.edu. جويل
christine.n.walters@okstate.eduكريستين والترز، جامعه ولاية أوكلاهوما،
hasina.rakotomanana@okstate.eduحسينهراكوتومانانا ، جامعه ولاية أوكلاهوما ،
deana.hildebrand@okstate.eduد. ديانا هيلدبيراند ، جامعه ولاية أوكلاهوما ،
Barbara.Stoecker@okstate.eduالدكتورة باربرا ستويكر ، جامعه ولاية أوكلاهوما ،
mkabahenda@caes.mak.ac.ugالدكتورة مارغريت كاباهندا ، جامعه ماكيريري كمبالا ،

:راعي الدراسة.

وقدم المحقق الرئيسي اقتراحا لتمويل البحوث إلى مؤسسه نستله ال$20,000 لا يزال قيد الاستعراض. ستسعى الوزارة أيضا إلى الحصول علي فرصه التمويل الداخلي داخل الجامعة من خلال منح صغيره للطلاب لاجراء البحوث من أجل تغطيه التكاليف التشغيلية في اجراء الدراسة.

:والأساس المنطقي للدراسة

وهناك نقص في المعونة الطارئة عندما ينتقل اللاجئون إلى مستوطنات ما بعد الطوارئ مما يجعل الأطفال أكثر عرضه لسوء التغذية. وفي المستوطنات التالية لحالات الطوارئ في غرب النيل، يلاحظ ارتفاع معدلات سوء التغذية الحاد في العالم. وهناك حاجه إلى برامج فعاله ومستدامه لمعالجه أسباب سوء تغذيه الأطفال. من المتوقع ان يكون تدخل الدعم الاجتماعي الذي يعالج النظافة الاساسيه وتغذيه الأطفال وممارسات نماء الطفل والصحة العقلية فعالا ومستداما في الحد من سوء تغذيه الأطفال بين اللاجئين في غرب النيل في مرحله ما بعد الطوارئ مستوطنات لمنع العواقب السلبية الطويلة الأجل لنقص تغذيه الأطفال.

:الغرض

والغرض من هذه الدراسة هو اختبار فعاليه التدخل التغذوي المتكامل الذي يقدم من خلال مجموعات الرعاية في تحسين ممارسات تغذيه الرضع والأطفال الصغار، ونمو الأطفال ونماءهم، والصحة العقلية للأمهات. ستساعد ردودك وقياساتك الباحثين على تقديم توصيات بشأن استخدام نموذج مجموعه الرعاية في تقديم برامج التغذية المتكاملة في المستقبل.

:الاجراءات

وصف إجراءات الدراسة التي تشرح كيفية مشاركه المشارك وما هو مطلوب من المشارك.
كما سنطرح عليك اسئله حول مختلف الموضوعات المتعلقة بتغذيه الرضع، ودعم الاقران، والصحة العقلية، وتحفيز الأطفال، ومشاركه الأب في رعاية الطفل والنظافة العامة في المنزل. ستستغرق مناقشه مجموعه التركيز حوالي ساعة واحده و30 دقيقه كحد أقصى. سيتم تسجيل المناقشات الجماعية بالصوت.

من سيشارك في الدراسة؟

وصف موجز للمشاركين المستهدفين، والعدد الإجمالي المتوقع والمدة التي سيطلب من كل منهم ان يكونوا نشطين في الدراسة.
سنقوم بتسجيل 8 مجموعات تركيز من الأمهات والاباء والمخبرين الرئيسيين الآخرين مثل العاملين في مجال الإرشاد الصحي المجتمعي وقاده القرى. ستتالف كل مجموعه من 8 افراد. ستستغرق مناقشه مجموعه التركيز حوالي ساعة واحده و30 دقيقه كحد أقصى.

:المخاطر/المضايقات

MAKERERE UNIVERSITY
SCHOOL OF HEALTH SCIENCES
APPROVED
VALID UNTIL
01 MAY 2020
RESEARCH & ETHICS COMMITTEE
P. O. BOX 7072, KAMPALA

.وصف المخاطر المحتملة والمضايقات التي قد يتعرض لها المشارك اثناء الدراسة
لا توجد مخاطر مرتبطة بهذا المشروع التي هي أكبر من تلك الموجودة عاده في الحياة اليومية

:فوائد
.الفوائد المتوقعة من اجراء الدراسة بما في ذلك الفوائد المحتملة للمشاركين والمجتمع والعالم العلمي بأكمله
سوف تستفيد من المناقشات الجماعية مع نظرائهم فيما يتعلق بتغذيه الأطفال، والصرف الصحي الأساسي، وتحفيز الأطفال في ومن المتوقع ان تسترشد النتائج .المنزل ، وأداره الإجهاد. كما ستعزز فهمك لهذه المواضيع وكيفية صلتها برعاية الطفل الاجماليه لأفرقه التركيز بتصميم تدخل المشروع الذي سيسترشد به في بروتوكول التدخل. لذلك، يعتمد نجاح الدراسة بقوة علي مخرجات المناقشة. سيجري أيضا اطلاع المنظمات غير الحكومية والجهات المعنية على الاستنتاجات لدعم توفير دروس التعلم .لأفضل الممارسات المتعلقة بالتبني والممارسات الرديءه لتحسين استراتيجيات التدخل التغذوي
:بدائل
المشاركة طوعية تماما ولا توجد عقوبة إذا كنت لا ترغب في المشاركة في الدراسة. إذا اخترت عدم المشاركة في الدراسة، وعلاوة على ذلك، يعتزم الباحثون .ستظل قادرا على الاستفادة من نتائج الدراسة استنادا إلى الدروس المستفادة من المشروع .تقديم طلب للحصول على منحه متابعه من شانها ان تصل إلى المزيد من الأسر المعيشية في المستوطنات التي تلي الطوارئ
:تكلفه
ستغطي تكاليف المواد الخاصة بالدراسة من قبل ممولي المنح الذين لا .لا توجد تكلفه بالنسبة لك للمشاركة في الدراسة الا وقتك .يهتمون الا بتطوير طرق أفضل لمكافحه سوء تغذيه الأطفال

:التعويض عن المشاركة في الدراسة
.شرح ما إذا كان سيتم تعويض المشارك عن المشاركة في الدراسة وكيف سيتم تعويضهم
لا توجد .لنقدرلكملوقتك (UGX 7,200) $2 وسوفنقدملكمعمايقربمن .سيتم منحك تعويضا ماليا لكونك في هذه الدراسة مخاطر مرتبطة بهذا المشروع والتي هي أكبر من تلك الموجودة عاده في الحياة اليومية، التالي، لا يتوقع اي أصابه لأي إذا كنت قد جرحت بطريقه أو بأخرى اثناء مشاركتك، سيتم توفير الإسعافات الاوليه (حسب الضرورة) ومن ثم سيتم .مشارك سيقوم الباحثون أيضا باعداد تقرير عن الحادث يتم تقاسمه مع .نقلك إلى أقرب مرفق صحي سيتم اخطار شخص ما في عائلتك السلطات المحلية في المستوطنة والمكتب الصحي للمقاطعات ومع مكاتب حماية المشاركين في الدراسات البحثية في جامعه .ماكيريري وولاية أوكلاهوما جامعه

:السداد
ستجري جميع مجموعات التركيز في مكان الاجتماع المشترك (مثل قاعه المجتمع المحلي أو مكان الاجتماع) داخل المجتمع المحلي المجاور الذي يمكن لجميع المشاركين الوصول اليه. سيكون الباحثون علي يقين من انه يمكنك المشاركة في منطقه .مريحه

:الاسئله
يمكن للمشاركين الوصول إلى المحققين من خلال منصتين، 1) ستقوم الشركة بالإبلاغ عن اليه الملاحظات الخاصة والذي يمكن للمشاركين استدعاؤه في اي وقت PI كهاتف محمول يعمل علي مدار الساعة بواسطة (PFM) بالمشاركين للإبلاغ عن اي مشكله، 2) يجوز للمشارك الاتصال برئيس مجلس المراجعة المؤسسية لكليه العلوم الصحية (256 +) 772-404970. Tel: (+ 256)-41-250431./(+ 256) 0200903786/أو المجلس الوطني الأوغندي للعلوم والتكنولوجيا

:اسئلهحولحقوقالمشاركين
إذا كان لديك اي اسئله يمكنك الوصول إلى الباحثين بطريقتين: 1) سيكون هناك هاتف محمول علي مدار 24 ساعة التي تحتفظ التي يمكن استدعاؤها في اي وقت ان لديك اسئله أو القلق، أو 2) يمكنك الاتصال بالرئيس، كليه العلوم الصحية، مجلس PI بها .المراجعة المؤسسية (256 +) 772-404970/(+ 256) 0200903786/أو المجلس الوطني الأوغندي للعلوم والتكنولوجيا
Tel: Tel: (+256)-41-4705500.

لاينطبق :البحوث التي تنطوي علي جمع المواد البشرية/عينات

MAKERERE UNIVERSITY
SCHOOL OF HEALTH SCIENCES
APPROVED
VALID UNTIL
01 MAY 2020
RESEARCH & ETHICS COMMITTEE
P. O. BOX 7072, KAMPALA

وفيما يتعلق بالتقدم المحرز في الدراسة ، سيشارك المعهد في :تعليقات علي نتائج الدراسة والتقدم المحرز في الدراسة الاجتماعات الروتينية لأصحاب المصلحة التي تعقدها قياده المستوطنات من أجل تقديم بيان موجز عن التقدم المحرز في الدراسة والنتائج الاوليه عند الاقتضاء. ستعقد اللجنة أيضا اجتماعا نهائيا للجات المعنية سيقدم فيه عرض للنتائج فيما يتعلق بالأهداف الرئيسية للدراسة. ستقدم الوزارة أيضا تقارير عن الفترة الزمنيه من خلال اجتماعات لجنه تنسيق التغذية في المقاطعات مع موظف الصحة في المقاطعة بشان الوضع العام للدراسة. سيتم تقاسم النتائج المرحلية من خلال الهيكل لامركزي كما ستقوم الشركة أيضا بالإبلاغ عن اليه الملاحظات .للنظام الصحي إلى المضغ الذي سيقدم تغذيه مرتده مباشره للمستفيدين والذي يمكن استدعاؤه في اي وقت من قبل PI كهاتف محمول يعمل علي مدار الساعة بواسطة (PFM) الخاصة بالمشاركين اي مشارك للإبلاغ عن مشكله تتعلق بالدراسة أو مشكله أمنيه يمكن إرسالها إلى السلطات الامنيه. ويمكن أيضا استخدام هذا النظام للاستفسار عن التقدم المحرز في الدراسة

:الموافقة علي الدراسة البحثية
وقد تمت الموافقة علي هذه الدراسة من قبل جامعه ماكيريري كليه العلوم الصحية لجنه البحوث والأخلاق/الهجرة

:بيانالتطوع
مشاركتك اختياريه تماما ولا توجد عقوبة إذا رفضت التسجيل في الدراسة. أنت حر أيضا في سحب موافقتك والمشاركة في هذا المشروع في اي وقت دون اي عقوبة

:الموافقة علي الدراسة البحثية
وقد تمت الموافقة علي هذه الدراسة من قبل جامعه ماكيريري كليه العلوم الصحية لجنه البحوث والأخلاق/الهجرة

:السريه
هويتيستخفيبقدر مايسمحبهالقانونلن يظهر اسمي في اي .ستبقي نتائج هذه الدراسة سريه للغاية ولا تستخدم الا لأغراض البحث مكان على النماذج المشفرة مع المعلومات. سيتم الاحتفاظ بسجلات الورق والكمبيوتر تحت القفل والمفتاح ومع حماية كلمه .المرور علي التوالي

وقد ناقش الباحث/المقابلة هذه المعلومات معي وعرض على الاجابه على أسئلتي. وللمزيد من الاسئله ، يمكنني الاتصال برئيس مجلس المراجعة المؤسسية لكليه العلوم الصحية علي (+ 256) 772-404970/(+ 256) 0200903786/أو المجلس الوطني .الأوغندي للعلوم والتكنولوجيا Tel: (+ 256)-41-250431

بيان الموافقة/التصديق
وقد وصفت لي ما سيتم القيام به ، والمخاطر ، والفوائد التي ....
ينطوي عليها وحقوقي فيما يتعلق بهذه الدراسة. وانا افهم ان قراري بالمشاركة في هذه الدراسة لن يغير صحتي المعتادة أو وافهم انه بتوقيعي هذا النموذج، .واناعليعلمبانيقدانسحبفيايوقت .الرعاية الطبية. في استخدام هذه المعلومات، سيتم إخفاء هويتي فاني لا أتخلى عن اي من حقوقي القانونية، ولكني اكتفي بالاشاره إلى اني قد أبلغت بالدراسة البحثية التي أوافق طوعا علي .المشاركة فيها. ستقدم لي نسخه من هذا النموذج

التاريخ ....اسم................ توقيع/بصمه إبهام المشارك... العمر
(DD/MM/YY)..
اسمالشاهد..
التاريخ ....توقيعالشاهد..
(DD/MM/YY)..
التاريخ ....اسم........................ توقيع/بصمه الإبهام للوالد أو الوصي للقصر
(DD/MM/YY)..
التاريخ ....اسم... توقيع المقابلة
(DD/MM/YY)..

MAKERERE UNIVERSITY
SCHOOL OF HEALTH SCIENCES
APPROVED
VALID UNTIL
01 MAY 2020
RESEARCH & ETHICS COMMITTEE
P. O. BOX 7072, KAMPALA

MAKERERE UNIVERSITY SCHOOL OF HEALTH SCIENCES RESEARCH & ETHICS COMMITTEE (Mak SHSREC)

Peer groups to improve feeding practices and child nutrition in post-emergency settlements in Uganda

Investigators:

Joel J. Komakech, Oklahoma State University, joel.komakech@okstate.edu
Christine Walters, Oklahoma State University, christine.n.walters@okstate.edu
Hasina Rakotomanana, Oklahoma State University, hasina.rakotomanana@okstate.edu
Dr. Deana Hildebrand, Oklahoma State University, deana.hildebrand@okstate.edu
Dr. Barbara Stoecker, Oklahoma State University, Barbara.Stoecker@okstate.edu
Dr. Margaret Kabahenda, Makerere University Kampala, mkabahenda@caes.mak.ac.ug

Study sponsor:
The principal investigator (PI) has submitted a research funding proposal to The Nestle Foundation of $20,000 still under review. The PI will also seek internal funding opportunity within the university through small grants for students to conduct research in order to cover operational costs in conducting the study.

Background and rationale for the study:
Emergency aid is lacking when refugees move into post-emergency settlements making children more likely to be undernourished. In the West Nile post-emergency settlements, high rates of global acute malnutrition are seen. Effective and sustainable programs are needed to address the causes of child malnutrition. A social support intervention addressing basic hygiene, child feeding, and child development practices, and mental health is expected to be effective and sustainable in reducing child malnutrition among refugee children in the West Nile post-emergency settlements to prevent the long-term negative consequences of child undernutrition.

Purpose:
The purpose of this study is to test the effectiveness of an integrated nutrition intervention delivered through care groups in improving infant and young child feeding practices, child growth and development and maternal mental health. Your responses and measurements will help the researchers make recommendations on using the care group model in delivering integrated nutrition programs in the future.

Procedures:
Description of the procedures of the study explaining how a participant will be involved and what is required of the participant.
We will also ask you questions about on various topics about infant feeding, peer support, mental health, and child stimulation, father's participation in childcare and general hygiene at home. The focus group discussion will last about one hour and 30 minutes maximum. The group discussions will be audio recorded.

MAKERERE UNIVERSITY
SCHOOL OF HEALTH SCIENCES
APPROVED
VALID [illegible]
01 MAY 2020
RESEARCH & ETHICS COMMITTEE
P. O. BOX 7072, KAMPALA

NOTE: Do not sign this consent form if it does not have an IRB approval stamp, or if the date has lapsed

Who will participate in the study?
Brief description of the intended participants, the expected total number and how long each will be required to be active in the study.
We will enroll 8 focus groups of mothers, fathers and other key informants such as Community Health Extension Workers and village leaders. Each group will consist of 8 individuals. The focus group discussion will last about one hour and 30 minutes maximum.

Risks/Discomforts:
Description of the possible risks and discomforts a participant might experience while in the study.
There are no risks associated with this project which are greater than those ordinarily found in daily life.

Benefits:
Anticipated benefits of conducting the study including possible benefits to the participant, community and the entire scientific world.
You will benefit from group discussions with their peers regarding child feeding, basic sanitation, child home stimulation, and stress management. You will also enhance your understanding of these topics and how it relates to child care.
The overall results of the focus groups are expected to inform the project intervention design which will inform the intervention protocol. Therefore, the success of the study strongly depends on the discussion outputs. The findings will also be shared with the non-government organizations (NGOs) and stakeholders to support provide learning lessons for best practices for adoption and poor practices for improvement on nutrition intervention strategies.

Alternatives:
Participation is completely voluntary and there is no penalty if you do not want to participate in the study. If you choose not to participate in the study, you would still be able to benefit from the results of the study based on lessons learned from the project. Furthermore, the researchers intend to apply for a follow-up grant which would reach out to more households in post-emergency settlements.

Cost:
There is no cost to you for participation in the study except your time. Costs of materials for the study will be covered by the grant funders whose interest is only in developing better ways to fight child malnutrition.

Compensation for participation in the study:
Explain if participant will be compensated for participating in the study and how they will be compensated.
Financial compensation will be given to you for being in this study. We will provide you with approximately$2 (UGX 7,200) to appreciate you for your time.
There are no risks associated with this project which are greater than those ordinarily found in daily life, therefore, no injury is expected for any participant.
If you were somehow injured during your participation, first aid will be provided (as deemed necessary) and then you would be transported to the nearest health facility and someone in your

MAKERERE UNIVERSITY
SCHOOL OF HEALTH SCIENCES
APPROVED
VALID UNTIL
01 MAY 2020
RESEARCH & ETHICS COMMITTEE
P. O. BOX 7072, KAMPALA

NOTE: Do not sign this consent form if it does not have an IRB approval stamp, or if the date has lapsed

family will be notified. The researchers will also prepare an incident report to be shared with the local authorities in the settlement and the district health office and with the offices for the protection of participants in research studies at Makerere University and Oklahoma State University.

Reimbursement:
All focus groups will be conducted at the common meeting place (for example a community hall, or meeting place) within the nearby community accessible to all participants. The researchers will be sure that you can participate in a convenient area.

Questions:
If you have any questions you can reach the researchers in two ways: 1) There will be a 24-hour mobile phone held by the PI that can be called at any time that you have questions or concern, or 2) You may contact the Chairperson, School of Health Sciences Institutional Review Board (MakSHS-IRB) on (+256) 772-404970 / (+256) 0200903786 / or Uganda National Council of Sciences and Technology. Tel: (+256)-41-4705500

Questions about participants rights:
If you have any questions you can reach the researchers in two ways: 1) There will be a 24-hour mobile phone held by the PI that can be called at any time that you have questions or concern, or 2) You may contact the Chairperson, School of Health Sciences Institutional Review Board (MakSHS-IRB) on (+256) 772-404970 / (+256) 0200903786 / or Uganda National Council of Sciences and Technology. Tel: (+256)-41-4705500

Research involving the collection of human materials/ samples:
Not applicable

Feedback on the study findings and progress of the study:
Regarding the progress of the study, the PI will participate in the routine stakeholders' meetings that are convened by the settlement leadership in order to give a brief account of the progress of the of the study and preliminary findings where necessary. The PI will also hold final stakeholders meeting in which a presentation of results will be provided regarding the main objectives of the study. The PI will also provide period reports through the district nutrition coordination committee meetings with the district health officer (DHO) on the general status of the study. The progress findings will be shared through the health system decentralized structure to the CHEWs who will provide direct feedback to the beneficiaries.
Also, the PI will also instate a participant's feedback mechanism (PFM) as a 24-hour mobile phone held by the PI that can be called anytime by any participant to report an issue related to the study or a security issue that can be forwarded to the security authorities. This system may also be used for inquiries on the progress of the study

Statement of voluntariness:
Your participation is completely voluntary and there is no penalty if you refuse to enrol in the study. You are also free to withdraw your consent and participation in this project at any time without any penalty

MAKERERE UNIVERSITY
SCHOOL OF HEALTH SCIENCES
APPROVED
VALID UNTIL
01 MAY 2020
RESEARCH & ETHICS COMMITTEE
P. O. BOX 7072, KAMPALA

NOTE: Do not sign this consent form if it does not have an IRB approval stamp, or if the date has lapsed

Approval of the research study:
This study has been approved by the Makerere university School of Health Sciences Research and Ethics Committee/IRB

Confidentiality:

The results of individual participants in this study will be kept strictly confidential and used only for research purposes. My identity will be concealed in as far as the law allows. My name and the name of my infant will be removed from all documents and replaced with a code number. Paper and computer records will be kept under lock and key and with password protection respectively.

The researcher/interviewer has discussed this information with me and offered to answer my questions. For any further questions, I may contact the Chairperson, School of Health Sciences Institutional Review Board (MakSHS-IRB) on (+256) 772-404970 / (+256) 0200903786 / or Uganda National Council of Sciences and Technology. Tel: (+256)-41-4705500

STATEMENT OF CONSENT

.... has described to me what is going to be done, the risks, the benefits involved and my rights regarding this study. I understand that my decision to participate in this study will not alter my usual health or medical care. In the use of this information, my identity will be concealed. I am aware that I may withdraw at any time. I understand that by signing this form, I do not waive any of my legal rights but merely indicate that I have been informed about the research study in which I am voluntarily agreeing to participate. A copy of this form will be provided to me.

Name.................. Signature/thumbprint of participant .... Age...............

Date (DD/MM/YY) ....

Name of Witness...................................... Signature of Witness.............................

Date (DD/MM/YY) ....

Name..............................Signature/thumbprint of parent or guardian for minors ....

Date (DD/MM/YY) ....

Name.. Signature of Interviewer ....

Date (DD/MM/YY) ....

MAKERERE UNIVERSITY
SCHOOL OF HEALTH SCIENCES
APPROVED
VALID UNTIL
01 MAY 2020
RESEARCH & ETHICS COMMITTEE
P. O. BOX 7072, KAMPALA

NOTE: Do not sign this consent form if it does not have an IRB approval stamp, or if the date has lapsed

(Mak SHSREC) ماكيريري كليه العلوم الصحية التابعة للجنة البحوث والأخلاقيات

مجموعات النظراء لتحسين ممارسات التغذية وتغذيه الأطفال في المستوطنات التي تلي حالات الطوارئ في أوغندا

:المحققين

joel.komakech@okstate.edu، جامعه ولاية أوكلاهوما،j. Komakech جويل
christine.n.walters@okstate.eduكريستين والترز، جامعه ولاية أوكلاهوما،
hasina.rakotomanana@okstate.eduحسينهراكوتومانانا ، جامعه ولاية أوكلاهوما ،
deana.hildebrand@okstate.eduد. ديانا هيلديبراند ، جامعه ولاية أوكلاهوما ،
Barbara.Stoecker@okstate.eduالدكتورة باربرا ستويكر ، جامعه ولاية أوكلاهوما ،
mkabahenda@caes.mak.ac.ugالدكتورة مارغريت كاباهندا ، جامعه ماكيريري كمبالا ،

:راعي الدراسة.

وقدم المحقق الرئيسي اقتراحا لتمويل البحوث إلى مؤسسه نستله ال$20,000 لا يزال قيد الاستعراض. ستسعى الوزارة أيضا إلى الحصول علي فرصه التمويل الداخلي داخل الجامعة من خلال منح صغيره للطلاب لاجراء البحوث من أجل تغطيه التكاليف التشغيلية في اجراء الدراسة.

:والأساس المنطقي للدراسة

وهناك نقص في المعونة الطارئة عندما ينتقل اللاجئون إلى مستوطنات ما بعد الطوارئ مما يجعل الأطفال أكثر عرضه لسوء التغذية. وفي المستوطنات التالية لحالات الطوارئ في غرب النيل، يلاحظ ارتفاع معدلات سوء التغذية الحاد في العالم. وهناك حاجه إلى برامج فعاله ومستدامه لمعالجه أسباب سوء تغذيه الأطفال. من المتوقع ان يكون تدخل الدعم الاجتماعي الذي يعالج النظافة الاساسيه وتغذيه الأطفال وممارسات نماء الطفل والصحة العقلية فعالا ومستداما في الحد من سوء تغذيه الأطفال بين اللاجئين في غرب النيل في مرحله ما بعد الطوارئ مستوطنات لمنع العواقب السلبية الطويلة الأجل لنقص تغذيه الأطفال.

:الغرض

والغرض من هذه الدراسة هو اختبار فعاليه التدخل التغذوي المتكامل الذي يقدم من خلال مجموعات الرعاية في تحسين ممارسات تغذيه الرضع والأطفال الصغار، ونمو الأطفال ونماءهم، والصحة العقلية للأمهات. ستساعد ردودك وقياساتك الباحثين على تقديم توصيات بشأن استخدام نموذج مجموعه الرعاية في تقديم برامج التغذية المتكاملة في المستقبل.

:الاجراءات

.وصف إجراءات الدراسة التي تشرح كيفية مشاركه المشارك وما هو مطلوب من المشارك
كما سنطرح عليك اسئله حول مختلف الموضوعات المتعلقة بتغذيه الرضع، ودعم الاقران، والصحة العقلية، وتحفيز الأطفال، ومشاركه الأب في رعاية الطفل والنظافة العامة في المنزل. ستستغرق مناقشه مجموعه التركيز حوالي ساعة واحده و30 دقيقه كحد أقصى. سيتم تسجيل المناقشات الجماعية بالصوت.

من سيشارك في الدراسة؟

.وصف موجز للمشاركين المستهدفين، والعدد الإجمالي المتوقع والمدة التي سيطلب من كل منهم ان يكونوا نشطين في الدراسة
سنقوم بتسجيل 8 مجموعات تركيز من الأمهات والاباء والمخبرين الرئيسيين الآخرين مثل العاملين في مجال الإرشاد الصحي المجتمعي وقاده القرى. ستتالف كل مجموعه من 8 افراد. ستستغرق مناقشه مجموعه التركيز حوالي ساعة واحده و30 دقيقه كحد أقصى.

MAKERERE UNIVERSITY
SCHOOL OF HEALTH SCIENCES
APPROVED
VALID UNTIL
01 MAY 2020
RESEARCH & ETHICS COMMITTEE
P. O. BOX 7072, KAMPALA

:المخاطر /المضايقات

.وصف المخاطر المحتملة والمضايقات التي قد يتعرض لها المشارك اثناء الدراسة
لا توجد مخاطر مرتبطة بهذا المشروع التي هي أكبر من تلك الموجودة عاده في الحياة اليومية

:فوائد
.الفوائد المتوقعة من اجراء الدراسة بما في ذلك الفوائد المحتملة للمشاركين والمجتمع والعالم العلمي بأكمله
سوف تستفيد من المناقشات الجماعية مع نظرائهم فيما يتعلق بتغذيه الأطفال، والصرف الصحي الأساسي، وتحفيز الأطفال في ومن المتوقع ان تسترشد النتائج .المنزل ، وأداره الإجهاد. كما ستعزز فهمك لهذه المواضيع وكيفية صلتها برعاية الطفل الاجماليه لأفرقه التركيز بتصميم تدخل المشروع الذي سيسترشد به في بروتوكول التدخل. لذلك، يعتمد نجاح الدراسة بقوة علي مخرجات المناقشة. سيجري أيضا اطلاع المنظمات غير الحكومية والجهات المعنية على الاستنتاجات لدعم توفير دروس التعلم .لأفضل الممارسات المتعلقة بالتبني والممارسات الرديءه لتحسين استراتيجيات التدخل التغذوي

:بدائل
المشاركة طوعية تماما ولا توجد عقوبة إذا كنت لا ترغب في المشاركة في الدراسة. إذا اخترت عدم المشاركة في الدراسة، وعلاوة على ذلك، يعتزم الباحثون .ستظل قادرا على الاستفادة من نتائج الدراسة استنادا إلى الدروس المستفادة من المشروع .تقديم طلب للحصول على منحه متابعه من شانها ان تصل إلى المزيد من الأسر المعيشية في المستوطنات التي تلي الطوارئ

:تكلفه
ستغطي تكاليف المواد الخاصة بالدراسة من قبل ممولي المنح الذين لا .لا توجد تكلفه بالنسبة لك للمشاركة في الدراسة الا وقتك .يهتمون الا بتطوير طرق أفضل لمكافحه سوء تغذيه الأطفال

:التعويض عن المشاركة في الدراسة
.شرح ما إذا كان سيتم تعويض المشارك عن المشاركة في الدراسة وكيف سيتم تعويضهم
لا توجد .لنقدرلكملوقتك (UGX 7,200) $2 وسوفنقدملكممعمايقربمن .سيتم منحك تعويضا ماليا لكونك في هذه الدراسة مخاطر مرتبطة بهذا المشروع والتي هي أكبر من تلك الموجودة عاده في الحياة اليومية، التالي، لا يتوقع اي أصابه لأي إذا كنت قد جرحت بطريقه أو بأخرى اثناء مشاركتك، سيتم توفير الإسعافات الاوليه (حسب الضرورة) ومن ثم سيتم .مشارك سيقوم الباحثون أيضا باعداد تقرير عن الحادث يتم تقاسمه مع .نقلك إلى أقرب مرفق صحي سيتم اخطار شخص ما في عائلتك السلطات المحلية في المستوطنة والمكتب الصحي للمقاطعات ومع مكاتب حماية المشاركين في الدراسات البحثية في جامعه .ماكيريري وولاية أوكلاهوما جامعه

:السداد
ستجري جميع مجموعات التركيز في مكان الاجتماع المشترك (مثل قاعه المجتمع المحلي أو مكان الاجتماع) داخل المجتمع المحلي المجاور الذي يمكن لجميع المشاركين الوصول اليه. سيكون الباحثون علي يقين من انه يمكنك المشاركة في منطقه .مريحه

:الاسئله
يمكن للمشاركين الوصول إلى المحققين من خلال منصتين، 1) ستقوم الشركة بالإبلاغ عن اليه الملاحظات الخاصة والذي يمكن للمشاركين استدعاؤه في اي وقت PI كهاتف محمول يعمل علي مدار الساعة بواسطة (PFM) بالمشاركين للابلاغ عن اي مشكله، 2) يجوز للمشارك الاتصال برئيس مجلس المراجعة المؤسسية لكليه العلوم الصحية (256 +) 772- .Tel: (+ 256)-41-250431 .404970/(+ 256) 0200903786/أو المجلس الوطني الأوغندي للعلوم والتكنولوجيا

:اسئلهحولحقوقالمشاركين
إذا كان لديك اي اسئله يمكنك الوصول إلى الباحثين بطريقتين: 1) سيكون هناك هاتف محمول علي مدار 24 ساعة التي تحتفظ بها PI التي يمكن استدعاؤها في اي وقت ان لديك اسئله أو القلق، أو 2) يمكنك الاتصال بالرئيس، كليه العلوم الصحية، مجلس .المراجعة المؤسسية (256 +) 772-404970/(+ 256) 0200903786/أو المجلس الوطني الأوغندي للعلوم والتكنولوجيا
Tel: Tel: (+256)-41-4705500.

لاينطبق :البحوث التي تنطوي علي جمع المواد البشرية/عينات

MAKERERE UNIVERSITY
SCHOOL OF HEALTH SCIENCES
APPROVED
VALID [illegible]
01 MAY 2020
RESEARCH & ETHICS COMMITTEE
P. O. BOX 7072, KAMPALA

وفيما يتعلق بالتقدم المحرز في الدراسة ، سيشارك المعهد في :تعليقات علي نتائج الدراسة والتقدم المحرز في الدراسة الاجتماعات الروتينية لأصحاب المصلحة التي تعقدها قياده المستوطنات من أجل تقديم بيان موجز عن التقدم المحرز في الدراسة والنتائج الاوليه عند الاقتضاء. ستعقد اللجنة أيضا اجتماعا نهائيا للجات المعنية سيقدم فيه عرض للنتائج فيما يتعلق بالأهداف الرئيسية للدراسة. ستقدم الوزارة أيضا تقارير عن الفترة الزمنيه من خلال اجتماعات لجنه تنسيق التغذية في المقاطعات مع موظف الصحة في المقاطعة بشان الوضع العام للدراسة. سيتم تقاسم النتائج المرحلية من خلال الهيكل لامركزي كما ستقوم الشركة أيضا بالإبلاغ عن اليه الملاحظات .للنظام الصحي إلى المضغ الذي سيقدم تغذيه مرتده مباشره للمستفيدين والذي يمكن استدعاؤه في اي وقت من قبل PI كهاتف محمول يعمل علي مدار الساعة بواسطة (PFM) الخاصة بالمشاركين اي مشارك للإبلاغ عن مشكله تتعلق بالدراسة أو مشكله أمنيه يمكن إرسالها إلى السلطات الامنيه. ويمكن أيضا استخدام هذا النظام للاستفسار عن التقدم المحرز في الدراسة

:الموافقة علي الدراسة البحثية
وقد تمت الموافقة علي هذه الدراسة من قبل جامعه ماكيريري كليه العلوم الصحية لجنه البحوث والأخلاق/الهجرة

:بيانالتطوع
مشاركتك اختياريه تماما ولا توجد عقوبة إذا رفضت التسجيل في الدراسة. أنت حر أيضا في سحب موافقتك والمشاركة في هذا المشروع في اي وقت دون اي عقوبة

:الموافقة علي الدراسة البحثية
وقد تمت الموافقة علي هذه الدراسة من قبل جامعه ماكيريري كليه العلوم الصحية لجنه البحوث والأخلاق/الهجرة

:السريه
هويتيستخفيبقدر مايسمحبهالقانونلن يظهر اسمي في اي .ستبقي نتائج هذه الدراسة سريه للغاية ولا تستخدم الا لأغراض البحث مكان على النماذج المشفرة مع المعلومات. سيتم الاحتفاظ بسجلات الورق والكمبيوتر تحت القفل والمفتاح ومع حماية كلمه المرور علي التوالي.

وقد ناقش الباحث/المقابلة هذه المعلومات معي وعرض على الاجابه على أسئلتي. وللمزيد من الاسئله ، يمكنني الاتصال برئيس مجلس المراجعة المؤسسية لكليه العلوم الصحية علي (+ 256) 772-404970/(+ 256) 0200903786/أو المجلس الوطني الأوغندي للعلوم والتكنولوجيا. Tel: (+ 256)-41-250431

بيان الموافقة/التصديق
وقد وصفت لي ما سيتم القيام به ، والمخاطر ، والفوائد التي ....
ينطوي عليها وحقوقي فيما يتعلق بهذه الدراسة. وانا افهم ان قراري بالمشاركة في هذه الدراسة لن يغير صحتي المعتادة أو وافهم انه بتوقيعي هذا النموذج، .واناعليعلمبانيقدانسحبفيايوقت .الرعاية الطبية. في استخدام هذه المعلومات، سيتم إخفاء هويتي فاني لا أتخلى عن اي من حقوقي القانونية، ولكني اكتفي بالاشاره إلى اني قد أبلغت بالدراسة البحثية التي أوافق طوعا علي المشاركة فيها. ستقدم لي نسخه من هذا النموذج.

التاريخ ....اسم................. توقيع/بصمه إبهام المشارك.. العمر
(DD/MM/YY)...

اسمالشاهد...

التاريخ ....توقيعالشاهد
(DD/MM/YY)...

التاريخ ....اسم.......................... توقيع/بصمه الإبهام للوالد أو الوصي للقصر
(DD/MM/YY)...

التاريخ ....اسم............................... توقيع المقابلة
(DD/MM/YY)...

MAKERERE UNIVERSITY
SCHOOL OF HEALTH SCIENCES
APPROVED
VALID UNTIL
01 MAY 2020
RESEARCH & ETHICS COMMITTEE
P. O. BOX 7072, KAMPALA

(Mak SHSREC) ماكيريري كليه العلوم الصحية التابعة للجنة البحوث والأخلاقيات

مجموعات النظراء لتحسين ممارسات التغذية وتغذيه الأطفال في المستوطنات التي تلي حالات الطوارئ في أوغندا

:المحققين

j. Komakech جويل، جامعه ولاية أوكلاهوما؛ joel.komakech@okstate.edu
كريستين والترز ، جامعه ولاية أوكلاهوما، christine.n.walters@okstate.edu
حسينهر اكوتومانانا ، جامعه ولاية أوكلاهوما ، hasina.rakotomanana@okstate.edu
د. ديانا هيلديبراند ، جامعه ولاية أوكلاهوما ، deana.hildebrand@okstate.edu
الدكتورة باربرا ستوبكر ، جامعه ولاية أوكلاهوما ، Barbara.Stoecker@okstate.edu
الدكتورة مارغريت كاباهندا ، جامعه ماكيريري كمبالا ، mkabahenda@caes.mak.ac.ug

:راعي الدراسة

وقدم المحقق الرئيسي اقتراحا لتمويل البحوث إلى مؤسسه نستله ال$20,000 لا يزال قيد الاستعراض. ستسعى الوزارة أيضا إلى الحصول علي فرصه التمويل الداخلي داخل الجامعة من خلال منح صغيره للطلاب لاجراء البحوث من أجل تغطيه التكاليف التشغيلية في اجراء الدراسة.

:والأساس المنطقي للدراسة

وهناك نقص في المعونة الطارئة عندما ينتقل اللاجئون إلى مستوطنات ما بعد الطوارئ مما يجعل الأطفال أكثر عرضه لسوء التغذية. وفي المستوطنات التالية لحالات الطوارئ في غرب النيل، يلاحظ ارتفاع معدلات سوء التغذية الحاد في العالم. وهناك حاجه إلى برامج فعاله ومستدامه لمعالجه أسباب سوء تغذيه الأطفال. من المتوقع ان يكون تدخل الدعم الاجتماعي الذي يعالج النظافة الاساسيه وتغذيه الأطفال وممارسات نماء الطفل والصحة العقلية فعالا ومستداما في الحد من سوء تغذيه الأطفال بين اللاجئين في غرب النيل في مرحله ما بعد الطوارئ مستوطنات لمنع العواقب السلبية الطويلة الأجل لنقص تغذيه الأطفال.

:الغرض

والغرض من هذه الدراسة هو اختبار فعاليه التدخل التغذوي المتكامل الذي يقدم من خلال مجموعات الرعاية في تحسين ممارسات تغذيه الرضع والأطفال الصغار ، ونمو الأطفال ونماءهم، والصحة العقلية للأمهات. ستساعد ردودك وقياساتك الباحثين على تقديم توصيات بشأن استخدام نموذج مجموعه الرعاية في تقديم برامج التغذية المتكاملة في المستقبل.

:الاجراءات

.وصف إجراءات الدراسة التي تشرح كيفية مشاركه المشارك وما هو مطلوب من المشارك
واسئله مثل كيف، ومتى، وماذا تطعم رضيعك. كما سنطرح عليك اسئله حول النظافة، ولعب .سنطرحعليكاسئلهحولمنزلك الأطفال، ودعم الاقران من الأمهات، ومشاركه الأب من خلال الدعم الاجتماعي والصحة العقلية للأمهات. ستستغرق المقابلة حوالي ساعة و30 دقيقه. سنقيسطولالرضيعووزنه. سنقيم أيضا تطوره بعد الولادة بفترة وجيزة، وفي سن 3 أشهر و6 أشهر و9 و12 شهرا.

من سيشارك في الدراسة؟
.وصف موجز للمشاركين المستهدفين، والعدد الإجمالي المتوقع والمدة التي سيطلب من كل منهم ان يكونوا نشطين في الدراسة
سوف نقوم بتسجيل 200 أسره مع النساء الحوامل في الثلث الثالث من الحمل. وفي 50 من هذه الأسر ، سيكون الأب أو الشريك (لعلاج واحد) في الدراسة. ومن المتوقع ان تكون نشطا في الدراسة لمده سنه واحده.

MAKERERE UNIVERSITY
SCHOOL OF HEALTH SCIENCES
APPROVED
VALID UNTIL
01 MAY 2020
RESEARCH & ETHICS COMMITTEE
P. O. BOX 7072, KAMPALA

:المخاطر/المضايقات

.وصف المخاطر المحتملة والمضايقات التي قد يتعرض لها المشارك اثناء الدراسة

لا توجد مخاطر مرتبطة بهذا المشروع التي هي أكبر من تلك الموجودة عاده في الحياة اليومية. فعلي سبيل المثال ، يتوقع الباحث ان الطفل الذي يجري وزنه ، والطول الذي يؤخذ به يمكن التشديد عليه علي الأرجح بسبب طبيعة المعدات أو التصور .بأنها قد تصيبها بالم

:فوائد

.الفوائد المتوقعة من اجراء الدراسة بما في ذلك الفوائد المحتملة للمشاركين والمجتمع والعالم العلمي بأكمله

سوف تستفيد من المناقشات الجماعية مرتين شهريا مع أصدقائك فيما يتعلق بتغذيه الأطفال، والصرف الصحي الأساسي، .وتحفيز الأطفال في المنزل، وأداره الإجهاد. سوف تتعلم كيف تتصل هذه المواضيع برعاية الطفل

.كما سيتم تدريبك على المهارات الاساسيه لتقييم طفلك لسوء التغذية خلال اجتماعات المجموعة التي تعقد مرتين في الأسبوع وسوف تتعلم أيضا كيفية تعليم الاباء الآخرين لتقييم أطفالهم لسوء التغذية. ومن المتوقع ان تساعد نتائج المشروع على التواصل بين مسؤولي التغذية الحكوميين ومجتمعك المحلي. ويمكن للشبكات الاجتماعية التي أنشئت خلال المشروع ان تواصل تقديم الدعم لكل من التحق بالدراسة ولكن أيضا للإباء المقبلين بتدريبات محدثه ومنتظمة للعاملين في مجال الإرشاد الصحي المجتمعي وزعماء النظراء. من مسؤول الصحة في المنطقة-التغذية. سيجري أيضا تبادل النتائج مع مديري المنظمات غير الحكومية وأصحاب المصلحة لتوفير دروس لأفضل الممارسات المتعلقة بالتبني والممارسات السيئة لتحسين التدخلات .التغذويه

:بدائل

المشاركة طوعية تماما ولا توجد عقوبة إذا كنت لا ترغب في المشاركة في الدراسة. إذا اخترت عدم المشاركة في الدراسة، وعلاوة على ذلك، يعتزم الباحثون .ستظل قادرا على الاستفادة من نتائج الدراسة استنادا إلى الدروس المستفادة من المشروع .تقديم طلب للحصول على منحه متابعه من شانها ان تصل إلى المزيد من الأسر المعيشية في المستوطنات التي تلي الطوارئ

:تكلفه

ستغطي تكاليف المواد الخاصة بالدراسة من قبل ممولي المنح الذين لا .لا توجد تكلفه بالنسبة لك للمشاركة في الدراسة الا وقتك .يهتمون الا بتطوير طرق أفضل لمكافحه سوء تغذيه الأطفال

:التعويض عن المشاركة في الدراسة

.شرح ما إذا كان سيتم تعويض المشارك عن المشاركة في الدراسة وكيف سيتم تعويضهم

لا توجد .لنقدرلكملوقتك (UGX 7,200) $2 وسوفنقدملكممعمايقربمن .سيتم منحك تعويضا ماليا لكونك في هذه الدراسة مخاطر مرتبطة بهذا المشروع والتي هي أكبر من تلك الموجودة عاده في الحياة اليومية، التالي، لا يتوقع اي أصابه لأي إذا كنت قد جرحت بطريقه أو بأخرى اثناء مشاركتك، سيتم توفير الإسعافات الاوليه (حسب الضرورة) ومن ثم سيتم .مشارك سيقوم الباحثون أيضا باعداد تقرير عن الحادث يتم تقاسمه مع .نقلك إلى أقرب مرفق صحي سيتم اخطار شخص ما في عائلتك السلطات المحلية في المستوطنة والمكتب الصحي للمقاطعات ومع مكاتب حماية المشاركين في الدراسات البحثية في جامعه .ماكيريري وولاية أوكلاهوما جامعه

:السداد

سيتم اجراء جميع التقييمات وجمع البيانات في السكن الخاص بك أو في مكان قريب في المجتمع. سيكون الباحثون علي يقين في موقع تختاره مجموعه الرعاية الخاصة caregroup ستعقد اجتماعات مجموعه .من انه يمكنك المشاركة في منطقه مريحه .ستكونمنطقهالاجتماعقريبهمنمنزلك .بك بتوجيه من قائد مجموعه الرعاية

:الاسئله

إذا كان لديك اي اسئله يمكنك الوصول إلى الباحثين بطريقتين: 1) سيكون هناك هاتف محمول علي مدار 24 ساعة التي تحتفظ التي يمكن استدعاؤها في اي وقت ان لديك اسئله أو القلق، أو 2) يمكنك الاتصال بالرئيس، كليه العلوم الصحية، مجلس PI بها .المراجعة المؤسسية (256 +) 772-404970/(+ 256) 0200903786/أو المجلس الوطني الأوغندي للعلوم والتكنولوجيا

Tel: (+ 256)-41-250431.

MAKERERE UNIVERSITY
SCHOOL OF HEALTH SCIENCES
APPROVED
VALID UNTIL
01 MAY 2020
RESEARCH & ETHICS COMMITTEE
P. O. BOX 7072, KAMPALA

:اسئلهحولحقوقالمشاركين
إذا كان لديك اي اسئله يمكنك الوصول إلى الباحثين بطريقتين: 1) سيكون هناك هاتف محمول علي مدار 24 ساعة التي تحتفظ PI بها التي يمكن استدعاؤها في اي وقت ان لديك اسئله أو القلق، أو 2) يمكنك الاتصال بالرئيس، كليه العلوم الصحية، مجلس المراجعة المؤسسية (256 +) 772-404970/(+ 256) 0200903786/أو المجلس الوطني الأوغندي للعلوم والتكنولوجيا.
Tel: (+256)-41-4705500

:البحوث التي تنطوي علي جمع المواد البشرية/عينات
لاينطبق

:تعليقات علي نتائج الدراسة والتقدم المحرز في الدراسة
وفيما يتعلق بالتقدم المحرز في الدراسة ، سيشارك المعهد في الاجتماعات الروتينية لأصحاب المصلحة التي تعقدها قياده المستوطنات من أجل تقديم بيان موجز عن التقدم المحرز في الدراسة والنتائج الاوليه عند الاقتضاء. ستعقد اللجنة أيضا اجتماعا نهائيا للجات المعنية سيقدم فيه عرض للنتائج فيما يتعلق بالأهداف الرئيسية للدراسة. ستقدم الوزارة أيضا تقارير عن الفترة الزمنيه من خلال اجتماعات لجنه تنسيق التغذية في المقاطعات مع موظف الصحة في المقاطعة بشان الوضع العام للدراسة. سيتم تقاسم النتائج المرحلية من خلال الهيكل لامركزي للنظام الصحي إلى المضغ الذي سيقدم تغذيه مرتده مباشره كهاتف محمول يعمل علي (PFM) كما ستقوم الشركة أيضا بالإبلاغ عن اليه الملاحظات الخاصة بالمشاركين .للمستفيدين والذي يمكن استدعاؤه في اي وقت من قبل اي مشارك للإبلاغ عن مشكله تتعلق بالدراسة أو مشكله PI مدار الساعة بواسطة أمنيه يمكن إرسالها إلى السلطات الامنيه. ويمكن أيضا استخدام هذا النظام للاستفسار عن التقدم المحرز في الدراسة

:الموافقة علي الدراسة البحثية
وقد تمت الموافقة علي هذه الدراسة من قبل جامعه ماكيريري كليه العلوم الصحية لجنه البحوث والأخلاق/الهجرة

:بيانالتطوع
مشاركتك اختياريه تماما ولا توجد عقوبة إذا رفضت التسجيل في الدراسة. أنت حر أيضا في سحب موافقتك والمشاركة في هذا المشروع في اي وقت دون اي عقوبة

:السريه
هويتيستخفييقدر .سيتم الاحتفاظ بسريه تامه لنتائج المشاركين الافراد في هذه الدراسة واستخدامها فقط لأغراض البحث سيتم الاحتفاظ بسجلات الورق .مايسمحبهالقانونستتم أزاله اسمي (إذا تم تمييزه) من كافة المستندات واستبداله برقم رمز والكمبيوتر تحت القفل والمفتاح ومع حماية كلمه المرور على التوالي.
وقد ناقش الباحث/المقابلة هذه المعلومات معي وعرض على الاجابه على أسئلتي. وللمزيد من الاسئله ، يمكنني الاتصال برئيس مجلس المراجعة المؤسسية لكليه العلوم الصحية علي (+ 256) 772-404970/(+ 256) 0200903786/أو المجلس الوطني الأوغندي للعلوم والتكنولوجيا. Tel: (+256)-41-4705500

بيان الموافقة/التصديق
وقد وصفت لي ما سيتم القيام به ، والمخاطر ، والفوائد التي ....
ينطوي عليها وحقوقي فيما يتعلق بهذه الدراسة. وانا افهم ان قراري بالمشاركة في هذه الدراسة لن يغير صحتي المعتادة أو الرعاية الطبية. وافهم انه بتوقيعي هذا النموذج، .واناعليعلمبانيقدانسحبفيايوقت .في استخدام هذه المعلومات، سيتم إخفاء هويتي فاني لا أتخلى عن اي من حقوقي القانونية، ولكني اكتفي بالاشاره إلى اني قد أبلغت بالدراسة البحثية التي أوافق طوعا علي المشاركة فيها. ستقدم لي نسخه من هذا النموذج.

التاريخ ....اسم................. توقيع/بصمه إبهام المشارك... العمر
(DD/MM/YY)..
اسمالشاهد..
التاريخ .... توقيعالشاهد..
(DD/MM/YY)..

MAKERERE UNIVERSITY
SCHOOL OF HEALTH SCIENCES
APPROVED
VALID [illegible]
01 MAY 2020
RESEARCH & ETHICS COMMITTEE
P. O. BOX 7072, KAMPALA

توقيع/بصمه الإبهام للوالد أو الوصي للقصر ....اسم... التاريخ
(DD/MM/YY)...
توقيع المقابلة ....اسم.. التاريخ
(DD/MM/YY)...

MAKERERE UNIVERSITY
SCHOOL OF HEALTH SCIENCES
APPROVED
VALID UNTIL
01 MAY 2020
RESEARCH & ETHICS COMMITTEE
P. O. BOX 7072, KAMPALA

Peér groups to improve feeding practices and reduce malnutrition among child refugees in post-emergency settlements in Uganda.

MOTHERS QUESTIONNAIRE

Information about survey questionnaire

Task	Date	Time start	Time end	Responsible	Remark
Survey					
Data entry					
Data entry confirmation					

Information about household

Item	Name	ID
Interviewer		
Adjumani		
Village		
Cohort		
Household ID		
Mother		
Baby		

When the survey is done, please remove this first page and keep it where it belongs to ensure the anonymity of the household.

MAKERERE UNIVERSITY
SCHOOL OF HEALTH SCIENCES
APPROVED
VALID [illegible]
01 MAY 2020
RESEARCH & ETHICS COMMITTEE
P. O. BOX 7072, KAMPALA

Section 1: SOCIODEMOGRAPHIC CHARACTERISTICS

Instruction: circle the responses from the given option and write if any other idea or answer is given.

No	Questions	Response	Remark
101	Age		
102	What is your main occupation?	1. Housewife<br>2. Farmer<br>3. Office employee (government or non-government)<br>4. Others_______	
103	What is your highest educational level?	1.Illiterate<br>2.Informal education<br>3.Formal education ______years<br>4.Higher education	
104	Who is the head of your household?	1.Father<br>2.Mother (yourself)<br>3.Other (specify)	
105	How many individuals live in your house permanently? (family size)		
106	What is your religion?		
107	Does your household own agricultural land?	1. Yes<br>2. No	
108	What is the size of your land? (add unit)		
109	Does your household have any animals?	1. Yes<br>2. No	
110	What types and how many animals? (Put numbers after each animal)	1. Ox<br>2. Cow<br>3. Goat<br>4. Sheep<br>5. Hen<br>6. Others	
111	What is the main source of your drinking water?	1. Rivers and lakes<br>2. Tube wells<br>3. Rainwater<br>4. Public tap water<br>5. I don't know	
112	How long is the walk to the water source?		
113	Who is in charge of fetching water?	1. Mother<br>2. Father	

MAKERERE UNIVERSITY
SCHOOL OF HEALTH SCIENCES
APPROVED
VALID [illegible]
01 MAY 2020
RESEARCH & ETHICS COMMITTEE
P. O. BOX 7072, KAMPALA

		3. Daughter/son<br>4. Other (specify)___________	
114	What sanitation facility do you have?	1. Ventilated Improved Pit latrine<br>2. Permanent Latrine<br>3. Temporary Latrine<br>4. Communal latrine<br>5. None<br>6. Other (specify)__	
114a	If latrines, do you share it with other households?	1. Yes<br>2. No	
114b	If yes, with how many other households do you share the latrine?		
115	How old were you when you first gave birth?		
116	How many living children do you have?		
117	For this last pregnancy, how many antenatal visits have you attended?		
118	For this last birth, where did you deliver?	1.Hospital<br>2.Local public health center<br>3.Private health center<br>4.Home<br>5.Other (specify)___________	
119	For this last birth, how many postnatal checks have you gone to?		
120	For this last birth, how big was your baby?	1.Very small<br>2.Smaller than average<br>3.Average<br>4.Larger than average<br>5.Very large<br>6.Don't know	
121	Has your last child had diarrhea recently?	1. No<br>2. Yes, in the last 24 hours<br>3. Yes, in the last 2 weeks<br>4. I don't know	
122	Has your last child had cough recently?	1. No<br>2. Yes, in the last 24 hours<br>3. Yes, in the last 2 weeks<br>4. I don't know	

MAKERERE UNIVERSITY
SCHOOL OF HEALTH SCIENCES
APPROVED
VALID
01 MAY 2020
RESEARCH & ETHICS COMMITTEE
P. O. BOX 7072, KAMPALA

123	Has your last child had fever recently?	1. No<br>2. Yes, in the last 24 hours<br>3. Yes, in the last 2 weeks<br>4. I don't know	
124	For the last week, how many times have you listened to the radio?	1.I have not listened to the radio<br>2.Once or twice<br>3.Almost every day	
125	For the last week, how many times have you read the newspaper?	1.I have not read the newspaper<br>2.Once or twice<br>3.Almost every day	
126	For the last week, how many times have you watched the TV?	1. I have not watched the TV<br>2.Once or twice<br>3.Almost every day	
127	Do your household own any of these items?	1. Cellphone<br>2. Bicycle<br>3. Lantern/flashlight<br>4. Radio<br>5. Motorcycle<br>6. Ox cart	
128	How many rooms do you have in your house?		
129	What is your floor made of?	1. Mud<br>2. Cement<br>3. Wood<br>4. Cow dung smear<br>5. Other	
130	What type of house do you live in?	1. Wooden house<br>2. Mud house<br>3. Brick house<br>4. Temporary shelter (plastic sheets)<br>5. Other	
131	How long have you been in the West Nile region?	1. $\leq$ 1 year<br>2. 2 years<br>3. 3 years<br>4. 4 years<br>5. $\geq$ 5years	
132	Which organizations have you interacted with regarding health? sanitation and nutrition	1. Never<br>2. Local community organization<br>3. Non-Governmental Organization<br>4. Government Agency<br>5. UNHCR or UN agency	

MAKERERE UNIVERSITY
SCHOOL OF HEALTH SCIENCES
APPROVED
VALID UNTIL
01 MAY 2020
RESEARCH & ETHICS COMMITTEE
P. O. BOX 7072, KAMPALA

		6. Other specify	
133	Which organizations have you interacted with regarding water, sanitation and hygiene?	1. Never<br>2. Local community organization<br>3. Non-Governmental Organization<br>4. Government Agency<br>5. UNHCR or UN agency<br>6. Other specify	
134	Which organizations have you interacted with regarding food and nutrition?	1. Never<br>2. Local community organization<br>3. Non-Governmental Organization<br>4. Government Agency<br>5. UNHCR or UN agency<br>6. Other specify	
135	From question 132-134, How long have you been exposed to these programs.<br>Health<br>Sanitation<br>Nutrition	Write years of engagement here<br>..<br>..<br>..	

SECTION 2: Household Food Insecurity Access Scale (HFIAS)

Instruction: circle the responses from the given option.

No	Questions	Response	Code
201	In the past four weeks, did you worry that your household would not have enough food?	0 = No (skip to 203)<br>1=Yes	
202	How often did this happen?	1 = Rarely (once or twice in the past four weeks)<br>2 = Sometimes (three to ten times in the past four weeks<br>3 = Often (more than ten times in the past four weeks)	
203	In the past four weeks, were you or any household member not able to eat the	0 = No (skip to 205)<br>1=Yes	

MAKERERE UNIVERSITY
SCHOOL OF HEALTH SCIENCES
APPROVED
VALID
01 MAY 2020
RESEARCH & ETHICS COMMITTEE
P. O. BOX 7072, KAMPALA

	kinds of foods you preferred because of a lack of resources?		
204	How often did this happen?	1 = Rarely (once or twice in the past four weeks)<br>2 = Sometimes (three to ten times in the past four weeks)<br>3 = Often (more than ten times in the past four weeks)	
205	In the past four weeks, did you or any household member have to eat a limited variety of foods due to a lack of resources?	0 = No (skip to 207)<br>1=Yes	
206	How often did this happen?	1 = Rarely (once or twice in the past four weeks)<br>2 = Sometimes (three to ten times in the past four weeks)<br>3 = Often (more than ten times in the past four weeks)	
207	In the past four weeks, did you or any household member have to eat some foods that you really did not want to eat because of a lack of resources to obtain other types of food?	0 = No (skip to 209)<br>1=Yes	
208	How often did this happen?	1 = Rarely (once or twice in the past four weeks)<br>2 = Sometimes (three to ten times in the past four weeks)<br>3 = Often (more than ten times in the past four weeks)	
209	In the past four weeks, did you or any household member have to eat a smaller meal than you felt you needed because there was not enough food?	0 = No (skip to 211)<br>0 = No (skip to 211)<br>1=Yes<br>1=Yes	
210	How often did this happen?	1 = Rarely (once or twice in the past four weeks)<br>2 = Sometimes (three to ten times in the past four weeks)<br>3 = Often (more than ten times in the past	

MAKERERE UNIVERSITY
SCHOOL OF HEALTH SCIENCES
APPROVED
VALID [illegible]
01 MAY 2020
RESEARCH & ETHICS COMMITTEE
P. O. BOX 7072, KAMPALA

		four weeks)	
211	In the past four weeks, did you or any other household member have to eat fewer meals in a day because there was not enough food?	0 = No (skip to 213)<br>1=Yes	
212	How often did this happen?	1 = Rarely (once or twice in the past four weeks)<br>2 = Sometimes (three to ten times in the past four weeks)<br>3 = Often (more than ten times in the past four weeks)	
213	In the past four weeks, was there ever no food to eat of any kind in your household because of lack of resources to get food?	0 = No (skip to 215)<br>1=Yes	
214	How often did this happen?	1 = Rarely (once or twice in the past four weeks)<br>2 = Sometimes (three to ten times in the past four weeks)<br>3 = Often (more than ten times in the past four weeks)	
215	In the past four weeks, did you or any household member go to sleep at night hungry because there was not enough food?	0 = No (skip to 217)<br>0 = No (skip to 217)<br>1=Yes<br>1=Yes	
216	How often did this happen?	1 = Rarely (once or twice in the past four weeks)<br>2 = Sometimes (three to ten times in the past four weeks)<br>3 = Often (more than ten times in the past four weeks	
217	In the past four weeks, did you or any household member go a whole day and night without eating anything because there was not enough food?	0 = No<br>1 = Yes	
218	How often did this happen?	1 = Rarely (once or twice in the past four weeks)<br>2 = Sometimes (three to ten times in the	

MAKERERE UNIVERSITY
SCHOOL OF HEALTH SCIENCES
APPROVED
VALID UNTIL
01 MAY 2020
RESEARCH & ETHICS COMMITTEE
P. O. BOX 7072, KAMPALA

		past four weeks)<br>3 = Often (more than ten times in the past four weeks)	
219	How long did last year's rice production last?		

SECTION 3: COMPLEMENTARY FEEDING KNOWLEDGE

Instruction: circle the responses from the given option.

No	Questions	Response	Code
301F	Where did you receive information about complementary feeding?<br>(Circle all that apply)	1. Health/community workers<br>2. Family members<br>3. Media<br>4. Other<br>5. Nowhere	
302F	Other than breastmilk, what infants should be given during the first 6 months?	1. Hot water<br>2. Sugary water<br>3. Nothing, breastmilk only<br>4. Soft foods<br>5. I don't know	
303F	When should breastfeeding stop?	1. 6 months<br>2. 12 months (1 year)<br>3. 18 months (1 year and half)<br>4. 2 years or more<br>5. I don't know	
304F	At what age should an infant be introduced to complementary feeding?	1. < 4 months<br>2. 4 - 6 months<br>3. 6-8 months<br>4. > 8 months<br>5. I don't know	
305F	Should a child be looked at in the eyes during feeding?	1. Yes<br>2. No	
306F	Should a child be forced to finish the plate when you feed him/her?	1. Yes<br>2. No	
307F	Should a child be talked to when you feed him/her?	1. Yes<br>2. No	
308F	Should a child be rushed to eat fast during feeding?	1. Yes<br>2. No	
309F	What should a 6 months old child be feed?	1. Soft foods (mashed or puree)<br>2. Semi-solid foods (watery rice)<br>3. Family foods<br>4. I don't know	

MAKERERE UNIVERSITY
SCHOOL OF HEALTH SCIENCES
APPROVED
VALID [illegible]
01 MAY 2020
RESEARCH & ETHICS COMMITTEE
P. O. BOX 7072, KAMPALA

310F	How many times a week does an infant need to consume meat, poultry, and/or fish?	1. Once a week<br>2. Three times a week<br>3. Every day<br>4. They can't eat these	
311F	How many times a week does an infant need to consume eggs?	1. Once a week<br>2. Three times a week<br>3. Every day<br>4. They can't eat these	
312F	How often should a 6 – 8 months child fed in a day?	1. Once<br>2. 2 – 3 times<br>3. 4 – 5 times<br>4. I don't know	
313F	How often should a 9 – 11 months child be fed in a day?	1. 2 times<br>2. 3 – 4 times<br>3. 5 – 6 times<br>4. I don't know	
314F	When can a child eat family foods without modification?	1. 6 months<br>2. 12 months<br>3. 18 months<br>4. I don't know	

SECTION 4: COMPLEMENTARY FEEDING PRACTICES

Instruction: Ask the following questions to the mother (or the caregiver) about their child aged 6-23 months. All of the questions relate to that child.

No	Questions	Response	Code
401	What is his/her birthday?<br><br>If the respondent does not know the exact birthdate, ask: Does he/shehave a health/vaccination card with the birthdate recorded? If the health/vaccination card/official document is shown and the respondent confirms the information is correct, record the date of birth as documented on the card. Also, record the birthweight if indicated.		
402	How many months old is your child?		
403	Check consistency (calendar of events, birth card)		
404	Was your childbreastfed yesterday during the day or at		

MAKERERE UNIVERSITY
SCHOOL OF HEALTH SCIENCES
APPROVED
VALID
01 MAY 2020
RESEARCH & ETHICS COMMITTEE
P. O. BOX 7072, KAMPALA

	night?		
405a	Next, I would like to ask you about some liquids that your child may have had yesterday during the day or at night.<br>Did he/she have any…:<br>Plain water?	1. Yes<br>2. No	
405b	Infant formula such as NAN1, NAN2, Nutricia, Gallia, etc.?	1. Yes<br>2. No	
405c	Milk such as tinned, powdered, or fresh animal milk?	1. Yes<br>2. No	
405d	Juice or juice drinks?	1. Yes<br>2. No	
405e	Clear broth?	1. Yes<br>2. No	
405f	Yogurt?	1. Yes<br>2. No	
405g	Thin porridge?	1. Yes<br>2. No	
405h	Any other liquids? (specify)	1. Yes<br>2. No	

MAKERERE UNIVERSITY
SCHOOL OF HEALTH SCIENCES
APPROVED
VALID UNTIL
01 MAY 2020
RESEARCH & ETHICS COMMITTEE
P. O. BOX 7072, KAMPALA

414	Please describe everything that your child ate yesterday during the day or night, whether at home or outside the home. For each meal, put bullet points.<br>Think about when (NAME) first woke up yesterday. Did (NAME) eat anything at that time? If yes: Please tell me everything (NAME) ate at that time. Probe: Anything else? Until respondent says nothing else. If no, continue to Question b).<br>__________<br>__________<br>__________<br>__________<br>__________<br>__________<br>b) What did he/she do after that? Did he/she eat anything at that time?<br>If yes: Please tell me everything he/she ate at that time. Probe: Anything else? Until respondent says nothing else.<br>__________<br>__________<br>__________<br>__________<br>Repeat question b) above until respondent says the child went to sleep until the next day.<br>__________<br>__________<br>__________<br>__________

MAKERERE UNIVERSITY
SCHOOL OF HEALTH SCIENCES
APPROVED
VALID [illegible]
01 MAY 2020
RESEARCH & ETHICS COMMITTEE
P. O. BOX 7072, KAMPALA

	If respondent mentions mixed dishes like a porridge, sauce or stew, probe:<br>c) What ingredients were in that (MIXED DISH)? Probe: Anything else? Until respondent says nothing else.

Section 5: WATER, SANITATION, AND HYGIENE KNOWLEDGE

Instruction: circle the responses from the given options.

No	Questions	Response	Code
501F	What is the safest source of drinking water for children?	*1.* Rivers and lakes<br>*2.* Tube wells<br>*3.* Rainwater<br>*4.* Public tap water<br>5. I don't know	
502F	What should be done before giving water for children?	1. Nothing<br>*2.* Warm it up<br>*3.* Boil<br>*4.* Add *water guard* or other safe water treatment agents<br>5. I don't know	

MAKERERE UNIVERSITY
SCHOOL OF HEALTH SCIENCES
APPROVED
VALID [illegible]
01 MAY 2020
RESEARCH & ETHICS COMMITTEE
P. O. BOX 7072, KAMPALA

503F	How should drinking water be stored?	1. Covered<br>2. Not covered<br>3. Not covered in a clean area<br>4. I don't know	
504F	What should be cleaned before feeding your baby?	1. The caregiver's hands only<br>2. The caregiver's hands and the baby's hands<br>3. The caregiver's hands, the baby's hands and the utensils<br>4. Nothing<br>5. I don't know	
505F	Should you wash your hands before preparing food?	1. Yes<br>2. No	
507F	Should you wash your hands after cleaning the child's bottom?	1. Yes<br>2. No	
508F	Should you wash your hands after defecation?	1. Yes<br>2. No	

Are these statements regarding children's hygiene important?

No	Questions	Response	Code
509F	Children need to have a clean space to crawl	1. Yes<br>2. No	
510F	Flies and mosquitoes should be avoided in the house	1. Yes<br>2. No	
511F	Chickens should not be kept in areas where children play	1. Yes<br>2. No	
512F	Children should sleep under bed nets	1. Yes<br>2. No	
513F	Children's feces should be removed safely	1. Yes<br>2. No	
514F	If yes, how?		

Section 6: WATER, SANITATION, AND HYGIENE PRACTICES

Instruction: observe the spot-checks and take note as per observed (circle what applies).

No	Questions	Response	Code
601	Mother clean	1.Yes<br>2. No	
602	Child clean	1. Yes	

MAKERERE UNIVERSITY
SCHOOL OF HEALTH SCIENCES
APPROVED
VALID [illegible]
01 MAY 2020
RESEARCH & ETHICS COMMITTEE
P. O. BOX 7072, KAMPALA

		2. No	
603	Diaper/bottom clean	1. Yes<br>2. No	
604	Compound clean	1. Yes<br>2. No	
605	Poultry feces in the house	1. Yes<br>2. No	
606	Baby feces in the house	1. Yes<br>2. No	
607	Water standing in the surroundings	1. Yes<br>2. No	
608	Unwashed utensils	1. Yes<br>2. No	
609	Drinking water covered	1. Yes<br>2. No	
610	House swept	1. Yes<br>2. No	
611	Garbage container in house<br>House swept	1. Yes<br>2. No	

SECTION 7: CHILD STIMULATION KNOWLEDGE

Instruction: circle the responses from the given option.
Are these statements regarding children important?

No	Questions	Response	Code
701F	Mothers need to talk to their infants	1. Yes<br>2. No	
702F	Mothers need to play with their children at least once a day	1. Yes<br>2. No	
703F	Mothers need to spend time in learning activities with their infants	1. Yes<br>2. No	
704F	Fathers need to be talk to their infants	1. Yes<br>2. No	

MAKERERE UNIVERSITY
SCHOOL OF HEALTH SCIENCES
APPROVED
VALID [illegible]
01 MAY 2020
RESEARCH & ETHICS COMMITTEE
P. O. BOX 7072, KAMPALA

705F	Fathers need to play with their children at least once a day	1. Yes<br>2. No	
706F	Fathers need to spend time in learning activities with their infants	1. Yes<br>2. No	

Section 8: CHILD HOME STIMULATION

Instruction: circle the responses from the given options.

No	Questions	Response	Code
801	Children seem to demand attention when their parents are busy, doing housework, for example. Do you usually respond to your child's demand for attention while you are working?	1. Yes<br>2. No	
802	Has your child done anything in the last week that pleased you very much?	1. Yes<br>2. No	
803	If yes, what?	1. Yes<br>2. No	
804a	What kinds of things do you have your child play with?<br>Toys made by an adult	1. Yes<br>2. No	
804b	Household objects	1. Yes<br>2. No	
804c	Materials from outside the house	1. Yes<br>2. No	
804d	Toys that make noise	1. Yes<br>2. No	
804e	Toys for building things	1. Yes<br>2. No	
804f	Toys for pretending like dolls	1. Yes<br>2. No	
804g	Other (specify)		

MAKERERE UNIVERSITY
SCHOOL OF HEALTH SCIENCES
APPROVED
VALID UNTIL
01 MAY 2020
RESEARCH & ETHICS COMMITTEE
P. O. BOX 7072, KAMPALA

805a	In the past week, on how many days did any adult in the family do the following with your child? (specify number)<br>Read books or look at picture books		
805b	Tell stories		
805c	Sing songs		
805d	Go to the market or store, or visiting outside the home		
805e	Play		
805f	Spend time in learning activities like naming objects		
805g	Sit with the child during the main meal of the day		
805h	Talk during meals		
806	How do you know when your child is hungry?<br>Circle all that apply	1.Cries<br>2.Asks for food, points, or uses gestures (but does not cry)<br>3.Other (specify)______	
807	When you serve your child food, how is it served?	1. Separate bowl<br>2. Common or shared family plate<br>3. Child has not started eating other foods	
808	What do you usually do to get your child to eat?<br>Circle all that apply	1. Nothing<br>2. Tell child to eat<br>3. Encourage, praise, play or hold<br>4. Give other types of food<br>5. Force, threaten, or hit<br>6. Other (specify)________	

SECTION 9: FATHER'S INVOLVEMENT IN CHILDCARE

Instruction: circle the responses from the given options.

How often does your husband/partner…?

MAKERERE UNIVERSITY
SCHOOL OF HEALTH SCIENCES
APPROVED
VALID UNTIL
01 MAY 2020
RESEARCH & ETHICS COMMITTEE
P. O. BOX 7072, KAMPALA

No	Questions	Response	Code
901	Contribute money to support the child regularly, paying for food	1.Never<br>2.Sometimes<br>3.Almost always	
903	Take the child to healthcare center since his/her birth, alone or with you	1.Never<br>2.Sometimes<br>3.Almost always	
903	Play and talk with the child daily	1.Never<br>2.Sometimes<br>3.Almost always	
904	Feed and take care of the child almost daily	1.Never<br>2.Sometimes<br>3.Almost always	
905	Hold and carry the child daily	1.Never<br>2.Sometimes<br>3.Almost always	
905	Teach things to the child	1.Never<br>2.Sometimes<br>3.Almost always	
906	Take care of the child when you are busy	1.Never<br>2.Sometimes<br>3.Almost always	
907	Advice you on matters regarding the child	1.Never<br>2.Sometimes<br>3.Almost always	

SECTION 10: MENTAL HEALTH

Instruction: circle the responses from the given options.

No	Questions	Response	Code
1001	Do you often have headaches?	*1.* Yes<br>2. No	
1002	Is your appetite poor?	*1.* Yes	

MAKERERE UNIVERSITY
SCHOOL OF HEALTH SCIENCES
APPROVED
VALID [illegible]
01 MAY 2020
RESEARCH & ETHICS COMMITTEE
P. O. BOX 7072, KAMPALA

		2. No	
1003	Do you sleep badly?	1. Yes<br>2. No	
1004	Are you easily frightened?	1. Yes<br>2. No	
1005	Do your hands shake?	1. Yes<br>2. No	
1006	Do you feel nervous, tense or worried?	1. Yes<br>2. No	
1007	Is your digestion poor?	1. Yes<br>2. No	
1008	Do you have trouble thinking clearly?	1. Yes<br>2. No	
1009	Do you feel unhappy?	1. Yes<br>2. No	
1010	Do you cry more than usual?	1. Yes<br>2. No	
1011	Do you find it difficult to enjoy your daily activities?	1. Yes<br>2. No	
1012	Do you find it difficult to make decisions?	1. Yes<br>2. No	
1013	Is your daily work suffering?	1. Yes<br>2. No	
1014	Are you unable to play a useful part in life?	1. Yes<br>2. No	
1015	Have you lost interest in things?	1. Yes<br>2. No	
1016	Do you feel that you are a worthless person?	1. Yes<br>2. No	
1017	Do you feel tired all the time?	1. Yes<br>2. No	
1018	Are you easily tired?	1. Yes<br>2. No	

MAKERERE UNIVERSITY
SCHOOL OF HEALTH SCIENCES
APPROVED
VALID UNTIL
01 MAY 2020
RESEARCH & ETHICS COMMITTEE
P. O. BOX 7072, KAMPALA

1019	Do you have uncomfortable feelings in your stomach?	1. Yes<br>2. No	

SECTION 11: SOCIAL SUPPORT

Instruction: circle the responses from the given options.

No	Questions	Response	Code
1101	Who generally supports you the most in your life?	1. Mother or mother-in-law<br>2. Husband<br>3. Siblings<br>4. Friend<br>5. Other (specify):	
1102	I visit with friends and relatives	1. Not at all<br>2. Rarely<br>3. Somehow<br>4. Mostly<br>5. As much as I like	
1103	I get help around the house	1. Not at all<br>2. Rarely<br>3. Somehow<br>4. Mostly<br>5. As much as I like	
1104	I get help with money in an emergency	1. Not at all<br>2. Rarely<br>3. Somehow<br>4. Mostly<br>5. As much as I like	
1105	I get praise for a good job done/completed	1. Not at all<br>2. Rarely<br>3. Somehow<br>4. Mostly<br>5. As much as I like	

MAKERERE UNIVERSITY
SCHOOL OF HEALTH SCIENCES
APPROVED
VALID UNTIL
01 MAY 2020
RESEARCH & ETHICS COMMITTEE
P. O. BOX 7072, KAMPALA

1106	I have people who care what happens to me	1.Not at all<br>2.Rarely<br>3.Somehow<br>4.Mostly<br>5.As much as I like	
1107	I get much needed love and affection	1.Not at all<br>2.Rarely<br>3.Somehow<br>4.Mostly<br>5.As much as I like	
1108	I get telephone calls from people I know	1.Not at all<br>2.Rarely<br>3.Somehow<br>4.Mostly<br>5.As much as I like	
1109	I get people visit to check on me and see if I am fine	1.Not at all<br>2.Rarely<br>3.Somehow<br>4.Mostly<br>5.As much as I like	
1110	I get chances to talk to someone about problems at work or with my housework	1.Not at all<br>2.Rarely<br>3.Somehow<br>4.Mostly<br>5.As much as I like	
1111	I get chances to talk to someone I trust about my personal problems	1.Not at all<br>2.Rarely<br>3.Somehow<br>4.Mostly<br>5.As much as I like	
1112	I get chances to talk about money matters	1.Not at all<br>2.Rarely<br>3.Somehow<br>4.Mostly<br>5.As much as I like	
1113	I get invitations to go out and do things with other people	1.Not at all<br>2.Rarely<br>3.Somehow	

MAKERERE UNIVERSITY
SCHOOL OF HEALTH SCIENCES
APPROVED
VALID [illegible]
01 MAY 2020
RESEARCH & ETHICS COMMITTEE
P. O. BOX 7072, KAMPALA

		4.Mostly<br>5.As much as I like	
1114	I get useful advice about important things in life	1.Not at all<br>2.Rarely<br>3.Somehow<br>4.Mostly<br>5.As much as I like	
1115	I get help when I needed transportation	1.Not at all<br>2.Rarely<br>3.Somehow<br>4.Mostly<br>5.As much as I like	
1116	I get help when I am sick in bed	1.Not at all<br>2.Rarely<br>3.Somehow<br>4.Mostly<br>5.As much as I like	
1117	Do you have anybody you turn to for suggestions about how to deal with a family problem?	1.Not at all<br>2.Rarely<br>3.Somehow<br>4.Mostly<br>5.As much as I like	
1118	I get help with daily chores when I am sick?	1.Not at all<br>2.Rarely<br>3.Somehow<br>4.Mostly<br>5.As much as I like	
1119	I have someone who I can have a good time with.	1.Not at all<br>2.Rarely<br>3.Somehow<br>4.Mostly<br>5.As much as I like	

CHILD DEVELOPMENT (6 months)

Instruction: circle the responses from the given options.

MAKERERE UNIVERSITY
SCHOOL OF HEALTH SCIENCES
APPROVED
VALID UNTIL
01 MAY 2020
RESEARCH & ETHICS COMMITTEE
P. O. BOX 7072, KAMPALA

How often do you observe these…?

No	Questions	Response	Code
	Does your baby make high-pitched squeals?	1. Yes<br>2. No	
	When playing with sounds, does your baby make grunting, growling, or other deep-toned sounds?	1. Yes<br>2. No	
	If you call your baby when you are out of sight, does she look in the direction of your voice?	1. Yes<br>2. No	
	When a loud noise occurs, does your baby turn to see where the sound came from?	1. Yes<br>2. No	
	Does your baby make sounds like "da", "ga", "ka" and "ba"?	1. Yes<br>2. No	
	If you copy the sounds your baby makes, does your baby repeat the same sounds back to you?	1. Yes<br>2. No	
	While your baby is on his/her back, does your baby lift his leg high enough to see his/her feet?	1. Yes<br>2. No	
	When your baby is on her tummy, does he/she straighten both arms and push his/her whole chest off the bed or floor?	1. Yes<br>2. No	
	Does your baby roll from his/her back to his/her tummy, getting both arms out from under him/her/	1. Yes<br>2. No	
	When you put your baby on the floor, does he/she lean on his/her hands while sitting? (If he/she already sits up straight without leaning on her hands, mark "yes")	1. Yes<br>2. No	
	If you hold both hands just to balance your baby, does he/she support his/her own weight while standing?	1. Yes<br>2. No	
	Does your baby get into a crawling position by getting up on his/her hands and knee?	1. Yes<br>2. No	
	Does your baby grab a toy you offer and look at it, wave about it, or chew it for about 1 minute?	1. Yes<br>2. No	
	Does your baby reach for or grasp a toy using both hands at once?	1. Yes<br>2. No	

MAKERERE UNIVERSITY
SCHOOL OF HEALTH SCIENCES
APPROVED
VALID [illegible]
01 MAY 2020
RESEARCH & ETHICS COMMITTEE
P. O. BOX 7072 KAMPALA

	finger or hand? (If he/she already picks up a small object the size of a pea, mark "yes")	2. No	
	Does your baby pick up a small toy, holding it in the center of her hand with her fingers around it?	1. Yes<br>2. No	
	Does your baby successfully pick up a crumb by using his/her thumb and all of his/her fingers in a raking motion, even if he/she isn't able to pick it up? (if he/she already picks up a crumb, mark "yes")	1. Yes<br>2. No /	
	Does your baby pick up a small toy with only one hand?	1. Yes<br>2. No	
	When in front of a large mirror, does your baby smile or coo at himself/herself?	1. Yes<br>2. No	
	Does your baby act differently toward strangers than he/she does with you and other familiar people? (Reactions to strangers may include staring, frowning, withdrawing, or crying)	1. Yes<br>2. No /	
	While lying on his/her back, does your baby play by grabbing his/her foot?	1. Yes<br>2. No	
	When in front of a large mirror, does your baby reach out to pat the mirror?	1. Yes<br>2. No	
	While your baby is on his/her back, does he/she put his/her foot in his/her mouth?	1. Yes<br>2. No	
	Does your baby try to get a toy that is out of reach? (he/she may roll, pivot, on his/her tummy, or crawl to get it)	1. Yes<br>2. No	

SECTION 12: CHILD DEVELOPMENT (9 months)

MAKERERE UNIVERSITY
SCHOOL OF HEALTH SCIENCES
APPROVED
VALID UNTIL
01 MAY 2020
RESEARCH & ETHICS COMMITTEE
P. O. BOX 7072, KAMPALA

Instruction: circle the responses from the given options.

How often do you observe these…?

No	Questions	Response	Code
	Does your baby make sounds like "da", "ga", "ka" and "ba"?	1. Yes	

How often do you observe these…?

No	Questions	Response	Code
	Does your baby make high-pitched squeals?	*1.* Yes<br>2. No	
	When playing with sounds, does your baby make grunting, growling, or other deep-toned sounds?	*1.* Yes<br>2. No	
	If you call your baby when you are out of sight, does she look in the direction of your voice?	*1.* Yes<br>2. No	
	When a loud noise occurs, does your baby turn to see where the sound came from?	*1.*Yes<br>2. No	
	Does your baby make sounds like "da", "ga", "ka" and "ba"?	*1.* Yes<br>2. No	
	If you copy the sounds your baby makes, does your baby repeat the same sounds back to you?	*1.* Yes<br>2. No /	
	While your baby is on his/her back, does your baby lift his leg high enough to see his/her feet?	*1.* Yes<br>2. No	
	When your baby is on her tummy, does he/she straighten both arms and push his/her whole chest off the bed or floor?	*1.* Yes<br>2. No	
	Does your baby roll from his/her back to his/her tummy, getting both arms out from under him/her/	*1.* Yes<br>2. No	
	When you put your baby on the floor, does he/she lean on his/her hands while sitting? (If he/she already sits up straight without leaning on her hands, mark "yes")	*1.* Yes<br>2. No	
	If you hold both hands just to balance your baby, does he/she support his/her own weight while standing?	*1.* Yes<br>2. No	
	Does your baby get into a crawling position by getting up on his/her hands and knee?	*1.* Yes<br>2. No	
	Does your baby grab a toy you offer and look at it, wave about it, or chew it for about 1 minute?	*1.* Yes<br>2. No	
	Does your baby reach for or grasp a toy using both hands at once?	*1.* Yes<br>2. No	
	Does your baby reach for a crumb and touch it with his/her	*1.* Yes	

MAKERERE UNIVERSITY
SCHOOL OF HEALTH SCIENCES
APPROVED
VALID UNTIL
01 MAY 2020
RESEARCH & ETHICS COMMITTEE
P.O. BOX 7072, KAMPALA

		2. No	
	If you copy the sounds your baby makes, does your baby repeat the same sounds back to you?	1. Yes<br>2. No	
	Does your baby make two similar sounds like "ba-ba", "da-da", or "ga-ga"? (sound does not mean anything)	1. Yes<br>2. No	
	If you ask your baby to, does he/she play at least one nursery game even if you don't show him/her the activity yourself (eg: "bye-bye", "بييكابوو", "التصفيق يديك", etc.)	1. Yes<br>2. No	
	Does your baby follow one sample command, such as "come here", "give it to me", or "put it back", without using gestures?	1. Yes<br>2. No	
	Does your baby say three words, such as "mama", "dada", and "baba" (a "word" is a sound or sounds your baby says consistently to mean someone or something)?	1. Yes<br>2. No	
	If you hold both hands just to balance your baby, does he/she support his/her own weight while standing?	1. Yes<br>2. No	
	When sitting on the floor, does your baby sit up straight for several minutes without using his/her hands for support?	1. Yes<br>2. No	
	When you stand your baby next to furniture or the crib rail, does she hold on without leaning her chest against the furniture for support?	1. Yes<br>2. No	
	While holding onto furniture, does your baby bend down and pick up a toy from the floor and then return to a standing position?	1. Yes<br>2. No	
	While holding onto furniture, does your baby lower himself/herself with control (without falling or flopping down)?	1. Yes<br>2. No	
	Does your baby walk beside furniture while holding on with only one hand?	1. Yes<br>2. No	
	Does your baby pick up a small toy with only one hand?	1. Yes<br>2. No	

MAKERERE UNIVERSITY
SCHOOL OF HEALTH SCIENCES
APPROVED
VALID UNTIL
01 MAY 2020
RESEARCH & ETHICS COMMITTEE
P. O. BOX 7072, KAMPALA

	Does your baby successfully pick up a crumb by using his/her thumb and all of his/her fingers in a raking motion (if he/she already picks up a crumb, mark "yes")	1. Yes<br>2. No	
	Does your baby pick up a small toy with the tips of his thumb and fingers?	1. Yes<br>2. No	
	After one or two tries, does your baby pick up a piece of string with his/her first finger and thumb?	1. Yes<br>2. No	
	Does your baby pick up a crumb with the tips of his/her thumb and a finger? He may rest his/her arm or hand on the table while doing it	1. Yes<br>2. No	
	Does your baby put a small toy down, without dropping it, and then take her hand off the toy?	1. Yes<br>2. No	
	While your baby is in his/her back, does she put her foot in his/her mouth?	1. Yes<br>2. No	
	Does your baby drink water or juice from a cup while you hold it?	1. Yes<br>2. No	
	Does your baby feed himself/herself a cookie? (or something similar)	1. Yes<br>2. No	
	When you hold out your hand and ask for his/her toy, does your baby offer it to you even if he/she doesn't let go of it? (if she already let's go of the toy into your hand, mark "yes")	1. Yes<br>2. No	
	When you dress your baby, does he/she push his arm through a sleeve once his/her arm is started in the hole of the sleeve?	1. Yes<br>2. No	
	When you hold out your hand and ask for her toy, does your baby let go of it into your hand?	1. Yes<br>2. No	

MAKERERE UNIVERSITY
SCHOOL OF HEALTH SCIENCES
APPROVED
VALID UNTIL
01 MAY 2020
RESEARCH & ETHICS COMMITTEE
P. O. BOX 7072, KAMPALA

HHID________ FATHERID__________

Peer groups to improve feeding practices and reduce malnutrition among child refugees in post-emergency settlements in Uganda.

FATHERS QUESTIONNAIRE

Information about survey questionnaire

Task	Date	Time start	Time end	Responsible	Remark
Survey					
Data entry					
Data entry confirmation					

Information about household

Item	Name	ID
Interviewer		
Adjumani		
Village		
Cohort		
Household ID		
Father		
Baby		

When the survey is done, please remove this first page and keep it where it in the locked box to ensure the anonymity of the household.

MAKERERE UNIVERSITY
SCHOOL OF HEALTH SCIENCES
APPROVED
VALID UNTIL
01 MAY 2020
RESEARCH & ETHICS COMMITTEE
P. O. BOX 7072, KAMPALA

HHID________ FATHERID__________

SECTION 1: COMPLEMENTARY FEEDING KNOWLEDGE

Instruction: circle the responses from the given option.

No	Questions	Response	Code
301F	Where did you receive information about complementary feeding? (Circle all that apply)	1. Health/community workers<br>2. Family members<br>3. Media<br>4. Other<br>5. Nowhere	
302F	Other than breastmilk, what infants should be given during the first 6 months?	1. Hot water<br>2. Sugary water<br>3. Nothing, breastmilk only<br>4. Soft foods<br>5. I don't know	
303F	When should breastfeeding stop?	1. 6 months<br>2. 12 months (1 year)<br>3. 18 months (1 year and half)<br>4. 2 years or more<br>5. I don't know	
304F	At what age should an infant be introduced to complementary feeding?	1. < 4 months<br>2. 4 - 6 months<br>3. 6-8 months<br>4. > 8 months<br>5. I don't know	
305F	Should a child be looked at in the eyes during feeding?	1. Yes<br>2. No	
306F	Should a child be forced to finish the plate when you feed him/her?	1. Yes<br>2. No	
307F	Should a child be talked to when you feed him/her?	1. Yes<br>2. No	
308F	Should a child be rushed to eat fast during feeding?	1. Yes<br>2. No	
309F	What should a 6 months old child be feed?	1. Soft foods (mashed or puree)<br>2. Semi-solid foods (watery rice)<br>3. Family foods<br>4. I don't know	
310F	How many times a week does an infant need to consume meat, poultry, and/or fish?	1. Once a week<br>2. Three times a week<br>3. Every day<br>4. They can't eat these	
311F	How many times a week does an infant need to consume eggs?	1. Once a week<br>2. Three times a week<br>3. Every day<br>4. They can't eat these	
312F	How often should a 6 – 8 months child fed in a day?	1. Once<br>2. 2 – 3 times	

MAKERERE UNIVERSITY
SCHOOL OF HEALTH SCIENCES
APPROVED
VALID [illegible]
01 MAY 2020
RESEARCH & ETHICS COMMITTEE
P. O. BOX 7072, KAMPALA

		3. 4 – 5 times<br>4. I don't know	
313F	How often should a 9 – 11 months child be fed in a day?	1. 2 times<br>2. 3 – 4 times<br>3. 5 – 6 times<br>4. I don't know	
314F	When can a child eat family foods without modification?	1. 6 months<br>2. 12 months<br>3. 18 months<br>4. I don't know	

MAKERERE UNIVERSITY
SCHOOL OF HEALTH SCIENCES
APPRO[illegible]
VA[illegible]
01 MAY 2020
RESEARCH & ETHICS COMMITTEE
P. O. BOX 7072, KAMPALA

HHID________ FATHERID__________

Section 2: WATER, SANITATION, AND HYGIENE KNOWLEDGE

Instruction: circle the responses from the given options.

No	Questions	Response	Code
501F	What is the safest source of drinking water for children?	1. Rivers and lakes<br>2. Tube wells<br>3. Rainwater<br>4. Public tap water<br>5. I don't know	
502F	What should be done before giving water for children?	1. Nothing<br>2. Warm it up<br>3. Boil<br>4. Add *water guard*or other safe water treatment agents<br>5. I don't know	
503F	How should drinking water be stored?	1. Covered<br>2. Not covered<br>3. Not covered in a clean area<br>4. I don't know	
504F	What should be cleaned before feeding your baby?	1. The caregiver's hands only<br>2. The caregiver's hands and the baby's hands<br>3. The caregiver's hands, the baby's hands and the utensils<br>4. Nothing<br>5. I don't know	
505F	Should you wash your hands before preparing food?	1. Yes<br>2. No	
507F	Should you wash your hands after cleaning the child's bottom?	1. Yes<br>2. No	
508F	Should you wash your hands after defecation?	1. Yes<br>2. No	

Are these statements regarding children's hygiene important?

No	Questions	Response	Code
509F	Children need to have a clean space to crawl	1. Yes<br>2. No	
510F	Flies and mosquitoes should be avoided in the house	1. Yes<br>2. No	
511F	Chickens should not be kept in areas where children play	1. Yes<br>2. No	
512F	Children should sleep under bed nets	1. Yes	

MAKERERE UNIVERSITY
SCHOOL OF HEALTH SCIENCES
APPROVED
VALID UNTIL
01 MAY 2020
RESEARCH & ETHICS COMMITTEE
P. O. BOX 7072, KAMPALA

		2. No	
513F	Children's feces should be removed safely	1. Yes<br>2. No	
514F	If yes, how?		

SECTION 3: CHILD DEVELOPMENT KNOWLEDGE

Instruction: circle the responses from the given option.

Are these statements regarding children important?

No	Questions	Response	Code
701F	Mothers need to talk to their infants	1. Yes<br>2. No	
702F	Mothers need to play with their children at least once a day	1. Yes<br>2. No	
703F	Mothers need to spend time in learning activities with their infants	1. Yes<br>2. No	
704F	Fathers need to be talk to their infants	1. Yes<br>2. No	
705F	Fathers need to play with their children at least once a day	1. Yes<br>2. No	
706F	Fathers need to spend time in learning activities with their infants	1. Yes<br>2. No	

SECTION 4: SOCIAL SUPPORT

Instruction: circle the responses from the given option.

No	Questions	Response	Code
1101	Who should generally support mothers the most in their life?	1. Mother or mother-in-law<br>2. Husband<br>3. Siblings<br>4. Friend<br>5. Other (specify):	
1102	Mothers should visit with friends and relatives	1. Not at all<br>2. Rarely<br>3. Somehow<br>4. Mostly<br>5. As much as she likes	
1103	Fathers should help around the house	1. Not at all<br>2. Rarely<br>3. Somehow<br>4. Mostly<br>5. As much as she likes	
1104	Fathers should support mothers with money in case of	1. Not at all	

MAKERERE UNIVERSITY
SCHOOL OF HEALTH SCIENCES
APPROVED
VALID UNTIL
01 MAY 2020
RESEARCH & ETHICS COMMITTEE
P. O. BOX 7072, KAMPALA

	an emergency	2. Rarely<br>3. Somehow<br>4. Mostly<br>5. As much as she likes	
1105	Mothers should get praise for a good job done/completed	1. Not at all<br>2. Rarely<br>3. Somehow<br>4. Mostly<br>5. As much as she likes	
1106	Fathers should give love and affection to their wives	1. Not at all<br>2. Rarely<br>3. Somehow<br>4. Mostly<br>5. As much as she likes	
1107	Mothers should be able to get telephone calls from people checking on them	1. Not at all<br>2. Rarely<br>3. Somehow<br>4. Mostly<br>5. As much as she likes	
1108	Mothers should be able to get visits from people checking on them and see if they are fine	1. Not at all<br>2. Rarely<br>3. Somehow<br>4. Mostly<br>5. As much as she likes	
1109	Mothers should get chances to talk to someone about problems at work or with their household	1. Not at all<br>2. Rarely<br>3. Somehow<br>4. Mostly<br>5. As much as she likes	
1110	Mothers should get chances to talk to someone they trust about their personal problems	1. Not at all<br>2. Rarely<br>3. Somehow<br>4. Mostly<br>5. As much as she likes	
1112	Fathers should talk about to their wives about money matters	1. Not at all<br>2. Rarely<br>3. Somehow<br>4. Mostly<br>5. As much as she likes	
1113	Mothers should be able to get invitations to go out and do things with other people	1. Not at all<br>2. Rarely<br>3. Somehow<br>4. Mostly<br>5. As much as she likes	
1114	Mothers should be able to get useful advice about important things in life	1. Not at all<br>2. Rarely	

MAKERERE UNIVERSITY
SCHOOL OF HEALTH SCIENCES
APPROVED
VALID UNTIL
01 MAY 2020
RESEARCH & ETHICS COMMITTEE
P. O. BOX 7072, KAMPALA

		3. Somehow<br>4. Mostly<br>5. As much as she likes	
1115	Fathers should support their wives with transportation when they need it	1. Not at all<br>2. Rarely<br>3. Somehow<br>4. Mostly<br>5. As much as she likes	
1116	Fathers should support their wives when they are sick in bed	1. Not at all<br>2. Rarely<br>3. Somehow<br>4. Mostly<br>5. As much as she likes	
1117	Mothers need somebody to turn to for suggestions about how to deal with a family problem?	1. Not at all<br>2. Rarely<br>3. Somehow<br>4. Mostly<br>5. As much as she likes	
1118	Fathers should help with daily chores when the spouse is sick?	1. Not at all<br>2. Rarely<br>3. Somehow<br>4. Mostly<br>5. As much as she likes	
1119	Mothers should have someone who they can have a good time with?	1. Not at all<br>2. Rarely<br>3. Somehow<br>4. Mostly<br>5. As much as she likes	

MAKERERE UNIVERSITY
SCHOOL OF HEALTH SCIENCES
APPROVED
VALI[illegible]
01 MAY 2020
RESEARCH & ETHICS COMMITTEE
P. O. BOX 7072, KAMPALA

Focus Group Discussions and Key Informant Interviews

Peer groups to improve feeding practices and child nutrition in post-emergency settlements in the West Nile region in Uganda.

Mothers Focus Group Discussions

Social support practices

1. Who takes care of a mother and the newborn baby after she has given birth? Follow up question: How long after childbirth does the mother stay at home with the child?

2. Is it important to you to be able to interact with friends, family or other groups of people?

3. Are there things your family or friends do, or could do for you to make you feel that they are important or useful to your life?

4. If you had any deep concerns/problems, who would you depend on? Elaborate why?

5. If you wanted to meet or visit other people, are you able to do that easily? If not, what would make it difficult to do so?

6. Do you feel that you are understood by friends and family? How important is that to you that you are understood?

Infant and young child feeding practices

1. If you had questions about breastfeeding your child, who would you ask? Please think of all the people you could go to for advice.

2. In your community, what are infants fed immediately after birth? Who feeds infants immediately after birth? Follow up: Is this a common practice?

3. Generally, what may you consider when feeding your less than a six-month child?

4. What do you consider in order to give your child complementary foods? How soon infants can they begin eating complementary foods? Why? What food would you feed a young child (maybe ask before they can walk?) Have you heard something about foods that a child cannot eat? (Probe eating eggs or other animal source food)

MAKERERE UNIVERSITY
SCHOOL OF HEALTH SCIENCES
APPROVED
VALID UNTIL
01 MAY 2020
RESEARCH & ETHICS COMMITTEE
P. O. BOX 7072, KAMPALA

5. In your household, what is the priority order of being served meals? Why? At what age would a child first taste regular family food?

6. How do you feed i) a less than six months baby that is sick? What do you feed them? Why?

a. Follow-up question: ii) more than six months baby that is sick? What do you feed them? Why?

7. How do people and your environment affect how you feed your child?

8. How many times per day do you cook complementary food? *Probe about how do they store the rest of the food if they only cooked once?*

What do you think are the benefits of breastfeeding?

9. At what age should children stop breastfeeding?

10. At what age should children start eating food?

11. What are some reasons children are given food before six months old?

12. What might a parent give a child to eat before they are six months old?

13. What are some reasons why mothers do not breastfeed their children?

14. Where do you get water to prepare meals for your child?

Paternal involvement in child feeding and care

1. Does your husband do activities which contribute to child feeding? (probe for examples)

2. If a husband/partner is not involved in activities towards child feeding, why do you think that is so?

3. How do you feel if your partner/husband helps with doing household chores?

4. How would you prefer that your husband/ partner support you in household chores (probe towards child feeding)?

5. How would your husband feel about you talking to friends, your family members or joining a social group in your community?

MAKERERE UNIVERSITY
SCHOOL OF HEALTH SCIENCES
APPROVED
VALID UNTIL
01 MAY 2020
RESEARCH & ETHICS COMMITTEE
P. O. BOX 7072, KAMPALA

6. If your husband were more involved in childcare (feeding, bathing, playing, etc.), what would the other fathers or his friends think/say?

7. If your husband were more involved in childcare (feeding, bathing, playing, etc.), what would the other mothers in the community think/say?

WASH practices

1. How important is it for you to raise your child in a clean environment? Why?

2. If you have questions about sanitation and hygiene regarding child feeding, who do you ask? Why?

3. If you wanted to have better hygiene practices, what would make it difficult to do that?

Child home stimulation

1. What do you do when you spend time with your child?

2. Is it important to you to find time to play/talk to your child? Why?

3. If you wanted to play and talk to your child more, what prevents you from doing so?

Mental health

1. What kind of feelings do mothers in your community have after giving birth?
2. Who do mothers talk to when they feel sad or upset?
a. Why?
3. How do mothers in the community respond to problems or stress?
4. Where would mothers in your community get help when they are stressed?
5. What ways do you think the community can help mothers feel less stressed?

Fathers Focus Group Discussions

Social support practices

1. Who takes care of a mother and the newborn baby after the mother has given birth?

MAKERERE UNIVERSITY
SCHOOL OF HEALTH SCIENCES
APPROVED
VALID UNTIL
01 MAY 2020
RESEARCH & ETHICS COMMITTEE
P. O. BOX 7072, KAMPALA

How long after childbirth does the mother stay at home with the child?

2. What would you do for your wife to make her feel that you are important or useful to her life?

3. How do you feel about your wife meeting or visiting other people?

4. Do you think it is important for your wife to interact with friends, family or other groups of people?

5. How would you feel about your wife sharing any deep concerns/problems with other trusted people? Elaborate why?

6. How do people and your environment affect how you feed your child?

Infant and young child feeding practices

1. When should complementary feeding of a child begin? What should they be fed? Why?

2. If you had questions about complementary feeding for your child who would you ask? Please give as many details as possible.

3. In your household, what is the priority order of being served meals? Why?

4. In your household, who decides what the child is fed? Why?

5. What may be the reason(s) your wife will not exclusively breastfeed for the first 6 months?

6. Are there reasons that it would be hard to begin feeding your infant complementary foods when they are six months old? Have you heard of foods that a child should not eat? What are the reasons?

7. How do you feed a1 less than six months baby that is sick?

    a. Follow-up question: 2 more than six months baby that is sick?

8. If you wanted to be more involved in feeding your child, would it be difficult to do so? Why or why not?

MAKERERE UNIVERSITY
SCHOOL OF HEALTH SCIENCES
APPROVED
VALID UNTIL
01 MAY 2020
RESEARCH & ETHICS COMMITTEE
P. O. BOX 7072, KAMPALA

9. What do you think are the benefits of breastfeeding?

10. What do you feel is your role in supporting mothers who are breastfeeding?

11. How do fathers in your community support breastfeeding mothers?

12. At what age should children stop breastfeeding?

13. At what age should children start eating food?

14. At what age can a young child have juice?

Paternal involvement in child feeding and care

1. Do you contribute towards child feeding? What do you do?

2. How do you feel about getting more involved in child feeding?

3. How do you feel about helping with household chores?

4. Is there a way a husband can support his wife with house chores? (probe towards child feeding, elaborate answers)?

5. How do you feel about your wife talking to friends, family members or other social groups in your community?

6. If you were more involved in childcare (feeding, bathing, playing, etc.), what would the other fathers think/say?

Child home stimulation

1. Is it important for you to find time to play/talk to your child? Why?

2. If you wanted to play and talk to your child more, what prevents you from doing so?

Mental health

1. What feelings do fathers have after a new child is born?

2. Who do fathers talk to when they feel sad or upset?

MAKERERE UNIVERSITY
SCHOOL OF HEALTH SCIENCES
APPROVED
VALID UNTIL
01 MAY 2020
RESEARCH & ETHICS COMMITTEE
P. O. BOX 7072, KAMPALA

3. How do fathers in the community respond to problems or stress?

4. What resources are in your community to help fathers when they are stressed?

5. What ways do you think the community can help fathers feel less stressed?

Key informant interviews

Social support practices

1. If parents had deep concerns or problems, who would they depend on or go to for help?

2. In your community, what are infants fed immediately after birth? Who feeds infants immediately after birth? Follow up: Is this a common practice?

3. Who takes care of the mother and the newborn baby after childbirth?

4. Is it easy or difficult for parents to get support/assistance from other people? (probe for financial, social, child care, etc.) Why?

5. Do fathers in your community get engaged in household chores and child feeding? Why or why not?

Infant and young child feeding practices

1. If parents have questions on child feeding practices, who would they ask?

2. Are there foods a young child should or cannot eat?

   a. Any religious restrictions or cultural taboos?

3. What resources are available for parents if they have questions about child feeding?

4. In a household, who usually provides or brings or buys the food?

5. In a household, what is the priority order among family members of being served meals in a household? Why?

6. In a household, who usually prepares food to be eaten? Who usually decides what to give the child? Why?

7. Is there something that would make it easier for parents to improve what their children eat? What makes it difficult?

MAKERERE UNIVERSITY
SCHOOL OF HEALTH SCIENCES
APPROVED
VALID UNTIL
01 MAY 2020
RESEARCH & ETHICS COMMITTEE
P. O. BOX 7072, KAMPALA

8. If parents wanted to have better hygiene practices, what would make it difficult to do that?

Paternal involvement in child feeding practices

1. What would women and other community members say if fathers were seen cooking food? Feeding their child? What would his male friends say?

2. What would community members say if fathers were seen doing household chores?

3. If fathers are not involved in activities of child feeding and household chores, are there reasons?

4. In the community, are there foods you have heard should not be fed to children? If so, what foods are those? *Are there specific age categories for the foods*?

5. What foods are usually fed to infants for their first complementary foods? Why?

Child home stimulation

1. Is it important for mothers to find time to play/talk to their child? Why?

2. Is it important for fathers to find time to play/talk to their child? Why?

Mental health

1. What feelings do parents have after a new child is born?
2. Who do parents talk to when they feel sad or upset?
3. How do parents in the community respond to problems or stress?
4. Would parents be willing to ask for help when they are stressed?
5. What resources are in your community to help parents when they are stressed?
6. What ways do you think the community can help parents feel less stressed?
7. What do you see as your role in helping community members overcome stress?

Reaching the Community

1. What would be the most effective way to reach/involve mothers?

MAKERERE UNIVERSITY
SCHOOL OF HEALTH SCIENCES
APPROVED
VALID UNTIL
01 MAY 2020
RESEARCH & ETHICS COMMITTEE
P. O. BOX 7072, KAMPALA

2. What would be the most effective way to reach/involve fathers?

3. Do many of the households have their extended family with them?

4. What would be the most effective way to teach mothers and fathers?

5. Is it appropriate to have a care group of fathers and mothers combined? If not, why?

6. Do most families have working mobile phones? Kept by father or mother? Is there a charge to receive text messages?

7. Is there a local radio station that broadcasts in Dinka or Arabic?

8. Is there a schedule for government health campaigns?

9. In your experience, what are difficulties our research team should avoid in interacting with the community

MAKERERE UNIVERSITY
SCHOOL OF HEALTH SCIENCES
APPROVED
VALID [illegible]
01 MAY 2020
RESEARCH & ETHICS COMMITTEE
P. O. BOX 7072, KAMPALA

مجموعات النظراء لتحسين ممارسات التغذية والحد من سوء التغذية بين الأطفال اللاجئين في المستوطنات التي تلي حالات الطوارئ في أوغندا.

الاماستبيان S

معلومات حول استبيان الاستطلاع

ملاحظه	المسؤوله عن	نهاية الوقت	بداية الوقت	تاريخ	المهمه
					الدراسهالاستقصائيه
					إدخال البيانات
					تاكيد إدخال البيانات

معلومات عن الاسره

معرف	اسم	البند
		المقابله
		ادجوماني
		قرية
		الفوج
		الهوية المنزلية
		الام
		الطفل

عند الانتهاء من المسح، يرجى أزاله هذه الصفحة الاولي والاحتفاظ بها حيث في المربع مقفلة لضمان عدم الكشف عن هويه الاسره المعيشية.

MAKERERE UNIVERSITY
SCHOOL OF HEALTH SCIENCES
APPROVED
VALID [illegible]
01 MAY 2020
RESEARCH & ETHICS COMMITTEE
P. O. BOX 7072, KAMPALA

القسم 1: الخصائص الاجتماعية والديمغرافية

التعليمات: دائره الردود من الخيار المعطي والكتابة إذا أعطيت اي فكره أو أجابه أخرى.

Nبني مصفر	الاسئله	استجابه	ملاحظه
101	العمر		
102	ما هو الاحتلال الرئيسي الخاص بك؟	ربه منزل<br>المزارعين<br>موظف في المكتب (حكومي أو غير حكومي)<br>آخرين	
103	ما هو مستواك التعليمي الأعلى؟	الاميين<br>التعليم غير النظامي<br>التعليم الرسمي _ _ السنهات<br>التعليم العالي	
104	من هو راس أسرتك؟	الاب<br>الام (نفسك)<br>غير ذلك (يرجى التحديد) __	
105	كم عدد الافراد الذين يعيشون في منزلك بشكل دائم؟ (حجم العائلة)		
106	ما هو دينك؟		
107	هل منزلك الزراعية الخاصة الأراضي؟	نعم<br>لا	
108	ما هو حجم أرضك؟ (أضافه وحده)		
109	هل عائلتك لديها اي الماشية؟	نعم<br>لا	
110	ما هي أنواع وكم العديد من الماشية؟<br>(وضع الأرقام بعد كل الحيوانية)	الثور<br>البقر<br>الماعز<br>الاغنام<br>الدجاجه<br>الاخرين	
111	ما هو المصدر الرئيسي لمياه الشرب الخاصة بك؟	الأنهار والبحيرات<br>أنبوب الآبار<br>مياه الامطار<br>مياه الصنبور العامة<br>لا أعرف	
112	كم من الوقت هو المشي إلى مصدر المياه؟		
113	من المسؤول عن جلب المياه؟	الام<br>الاب<br>الابنة/الابن<br>غير ذلك (يرجى التحديد) __	
114	ما هو مرفق الصرف الصحي لديك؟	التهويه مرحاض الحفرة المحسن<br>الدائم المراحيض	

MAKERERE UNIVERSITY
SCHOOL OF HEALTH SCIENCES
APPROVED
VALID UNTIL
01 MAY 2020
RESEARCH & ETHICS COMMITTEE
P.O. BOX 7072 KAMPALA

		المراحيض المؤقتة<br>المراحيض المجتمعية<br>اي<br>غير ذلك (يرجى التحديد) __	
114a	إذا كانت المراحيض، هل تشاركها مع الأسر الأخرى؟	نعم<br>لا	
114b	إذا كانت الاجابه بنعم، مع عدد الأسر الأخرى التي تشترك في المراحيض؟		
115	كم كان عمرك عندما أنجبت لأول مره؟		
116	كم عدد الأطفال الذين يعيشون لديك؟		
117	لهذا الحمل الأخير، كم عدد الزيارات السابقة للولادة التي حضرتها؟		
118	لهذه الولادة الاخيره، أين سلمت؟	المستشفي<br>المركز المحلي للصحة العامة<br>المركز الصحي الخاص<br>المنزل<br>غير ذلك (يرجى التحديد) __	
119	لهذه الولادة الاخيره، كم عدد فحوصات ما بعد الولادة التي ذهبت اليها؟		
120	لهذه الولادة الاخيره، كم كان حجم طفلك؟	صغيره جدا<br>أصغر من المتوسط<br>متوسط<br>أكبر من المتوسط<br>كبير جدا<br>لا أعرف	
121	هل كان طفلك الأخير مصابا بالإسهال مؤخرا؟	لا<br>نعم، في الساعات ال 24 الاخيره<br>نعم، في الأسبوعين الماضيين<br>لا أعرف	
122	هل كان طفلك الأخير قد سعال مؤخرا؟	لا<br>نعم، في الساعات ال 24 الاخيره<br>نعم، في الأسبوعين الماضيين<br>لا أعرف	
123	هل كان طفلك الأخير مصابا بالحمى مؤخرا؟	لا<br>نعم، في الساعات ال 24 الاخيره<br>نعم، في الأسبوعين الماضيين<br>لا أعرف	
124	في الأسبوع الماضي، كم مره استمعت للراديو؟	انا لم استمع إلى الراديو<br>مره أو مرتين<br>تقريبا كل يوم	
125	في الأسبوع الماضي، كم مره قرات الصحيفة؟	انا لم أقرا الصحيفة	

MAKERERE UNIVERSITY
SCHOOL OF HEALTH SCIENCES
APPROVED
VALID UNTIL
01 MAY 2020
RESEARCH & ETHICS COMMITTEE
P. O. BOX 7072, KAMPALA

		مره أو مرتين<br>تقريبا كل يوم	
126	في الأسبوع الماضي، كم مره شاهدت التلفاز؟	لم أشاهد التلفاز<br>مره أو مرتين<br>تقريبا كل يوم	
127	هل تملك أسرتك أيا من هذه العناصر؟	المحمول<br>الدراجات<br>فانوس/مصباح يدوي<br>راديو<br>دراجة ناريه<br>عربه الثور	
128	كم عدد الغرف التي لديك في منزلك؟		
129	ما هو الطابق الخاص بك مصنوعة من؟	الطين<br>الاسمنت<br>الخشب<br>البقرة الروث مسحه<br>الاخري	
130	ما نوع المنزل الذي تعيش فيه؟	منزل خشبي<br>طين منزل<br>منزل من الطوب<br>ماوي مؤقت (صفائح بلاستيكية)<br>الاخري	
131	منذ متى وأنت في منطقه غرب النيل؟	اقل أو سنه واحده<br>سنتان<br>3 سنوات<br>4 سنوات<br>5 سنوات أو أكثر	
132	ما هي المنظمات التي تفاعلت معها فيما يتعلق بالصحة؟	ابدا<br>تنظيم المجتمع المحلي<br>المنظمات غير الحكومية<br>الوكالة الحكومية<br>المفوضيهأو وكاله الأمم المتحدة<br>أخرى يعين	
133	ما هي المنظمات التي تفاعلت معها فيما يتعلق بالمياه والصرف الصحي والنظافة؟	ابدا<br>تنظيم المجتمع المحلي<br>المنظمات غير الحكومية<br>الوكالة الحكومية<br>المفوضيهأو وكاله الأمم المتحدة<br>أخرى يعين	
134	ما هي المنظمات التي تفاعلت معها فيما يتعلق بالغذاء والتغذية؟	ابدا<br>تنظيم المجتمع المحلي<br>المنظمات غير الحكومية<br>الوكالة الحكومية<br>المفوضيهأو وكاله الأمم المتحدة<br>أخرى يعين	
135	من السؤال 132-134، منذ متى تعرضت	كتابه سنوات من المشاركة هنا	

MAKERERE UNIVERSITY
SCHOOL OF HEALTH SCIENCES
APPROVED
VALID UNTIL
01 MAY 2020
RESEARCH & ETHICS COMMITTEE
P. O. BOX 7072, KAMPALA

	....<br>..<br>..	لهذه البرامج.	

القسم 2: مقياس الوصول إلى انعدام الأمن الغذائي للأسر المعيشية (hfias)

التعليمات: دائره الاستجابات من الخيار المعطي.

التعليمات البرمجيه	استجابه	الاسئله	Nبني مصفر
	0 = لا (تخطي إلى 203)<br>1 = نعم	في الأسابيع الاربعه الماضية، هل كنت قلقا من ان أسرتك لن يكون لديها ما يكفي من الطعام؟	201
	1 = نادرا (مره أو مرتين في الأسابيع الاربعه الماضية)<br>2 = أحيانا (ثلاث إلى عشر مرات في الأسابيع الاربعه الماضية)<br>3 = في كثير من الأحيان (أكثر من عشر مرات في الأسابيع الاربعه الماضية)	كم مره حدث هذا؟	202
	0 = لا (تخطي إلى 205)<br>1 = نعم	في الأسابيع الاربعه الماضية، هل كنت أنت أو اي فرد من افراد الاسره المعيشية غير قادر علي تناول أنواع الاطعمه التي تفضلها بسبب نقص الموارد؟	203
	1 = نادرا (مره أو مرتين في الأسابيع الاربعه الماضية)<br>2 = أحيانا (ثلاث إلى عشر مرات في الأسابيع الاربعه الماضية)<br>3 = في كثير من الأحيان (أكثر من عشر مرات في الأسابيع الاربعه الماضية)	كم مره حدث هذا؟	204
	0 = لا (تخطي إلى 207)<br>1 = نعم	في الأسابيع الاربعه الماضية، هل يجب عليك أو على اي فرد من افراد الاسره تناول مجموعه محدوده من الاطعمه بسبب نقص الموارد؟	205
	1 = نادرا (مره أو مرتين في الأسابيع الاربعه الماضية)<br>2 = أحيانا (ثلاث إلى عشر مرات في الأسابيع الاربعه الماضية)	كم مره حدث هذا؟	206

MAKERERE UNIVERSITY
SCHOOL OF HEALTH SCIENCES
APPROVED
VALID UNTIL
01 MAY 2020
RESEARCH & ETHICS COMMITTEE
P. O. BOX 7072, KAMPALA

		3 = في كثير من الأحيان (أكثر من عشر مرات في الأسابيع الاربعه الماضية)	
207	في الأسابيع الاربعه الماضية، هل أنت أو اي فرد من افراد الاسره المعيشية يجب ان ياكل بعض الاطعمه التي كنت حقا لا تريد ان تاكل بسبب نقص الموارد للحصول على أنواع أخرى من الغذاء؟	0 = لا (تخطي إلى 209)<br>1 = نعم	
208	كم مره حدث هذا؟	1 = نادرا (مره أو مرتين في الأسابيع الاربعه الماضية)<br>2 = أحيانا (ثلاث إلى عشر مرات في الأسابيع الاربعه الماضية)<br>3 = في كثير من الأحيان (أكثر من عشر مرات في الأسابيع الاربعه الماضية)	
209	في الأسابيع الاربعهالماضية، هل أنت أو اي فرد من افراد الاسره المعيشية يجب ان ياكل وجبه أصغر مما كنت تشعر أنك بحاجه لأنه لم يكن هناك ما يكفي من الطعام؟	لا (تخطي إلى 211) = 0<br>نعم = 1	
210	كم مره حدث هذا؟	1 = نادرا (مره أو مرتين في الأسابيع الاربعه الماضية)<br>2 = أحيانا (ثلاث إلى عشر مرات في الأسابيع الاربعه الماضية)<br>3 = في كثير من الأحيان (أكثر من عشر مرات في الأسابيع الاربعه الماضية)	
211	في الأسابيع الاربعه الماضية، هل لديك أو اي عضو آخر في الاسره تناول وجبات اقل في يوم واحد لأنه لم يكن هناك ما يكفي من الطعام؟	0 = لا (تخطي إلى 213)<br>1 = نعم	
212	كم مره حدث هذا؟	1 = نادرا (مره أو مرتين في الأسابيع الاربعه الماضية)<br>2 = أحيانا (ثلاث إلى عشر مرات في الأسابيع الاربعه الماضية)<br>3 = في كثير من الأحيان (أكثر من عشر مرات في الأسابيع الاربعه الماضية)	
213	في الأسابيع الاربعه الماضية، لم يكن هناك اي طعام للأكل من اي نوع في منزلك بسبب نقص الموارد للحصول على الغذاء؟	0 = لا (تخطي إلى 215)<br>1 = نعم	

MAKERERE UNIVERSITY
SCHOOL OF HEALTH SCIENCES
APPROVED
VALID [illegible]
01 MAY 2020
RESEARCH & ETHICS COMMITTEE
P. O. BOX 7072, KAMPALA

214	كم مره حدث هذا؟	1 = نادرا (مره أو مرتين في الأسابيع الاربعه الماضية)<br>2 = أحيانا (ثلاث إلى عشر مرات في الأسابيع الاربعه الماضية)<br>3 = في كثير من الأحيان (أكثر من عشر مرات في الأسابيع الاربعه الماضية)	
215	في الأسابيع الاربعهالماضية، هل ذهبت أنت أو اي فرد من افراد الاسره إلى النوم في الليل جائعا لأنه لم يكن هناك ما يكفي من الطعام؟	لا (تخطي إلى 217) = 0<br>نعم = 1	
216	كم مره حدث هذا؟	1 = نادرا (مره أو مرتين في الأسابيع الاربعه الماضية)<br>)<br>2 = أحيانا (ثلاث إلى عشر مرات في الأسابيع الاربعه الماضية)<br>3 = في كثير من الأحيان (أكثر من عشر مرات في الأسابيع الاربعه الماضية)	
217	في الأسابيع الاربعهالماضية، هل ذهبت أنت أو اي فرد من افراد الاسره المعيشية طوال النهار والليل دون تناول اي شيء لأنه لم يكن هناك ما يكفي من الطعام؟	0 = لا<br>1 = نعم	
218	كم مره حدث هذا؟	1 = نادرا (مره أو مرتين في الأسابيع الاربعه الماضية)<br>2 = أحيانا (ثلاث إلى عشر مرات في الأسابيع الاربعه الماضية)<br>3 = في كثير من الأحيان (أكثر من عشر مرات في الأسابيع الاربعه الماضية)	
219	كم مضي على إنتاج الأرز العام الماضي؟		

القسم 3: المعارف التكميلية للتغذية

التعليمات: دائره ردود من الخيار المعطي.

Nبني مصفر	الاسئله	استجابه	التعليمات البرمجيه
301F	أين تلقيت المعلوماتيةأيون عن التغذية التكميلية؟ (دائره كل ما ينطبق)	العاملون في الصحة/المجتمع<br>افراد الاسره<br>وسائل الاعلام<br>الاخري<br>مكان	
302F	بخلاف حليب الثدي، ما الرضع ان يكون اعطاءnخلال	الماء الساخن	

MAKERERE UNIVERSITY
SCHOOL OF HEALTH SCIENCES
APPROVED
VALID UNTIL
01 MAY 2020
RESEARCH & ETHICS COMMITTEE
P. O. BOX 7072, KAMPALA

	الأشهر السنة الاولي؟	المياه السكرية<br>لا شيء، حليب الثدي فقط<br>الاطعمه الطرية<br>لا أعرف	
303F	متى يجب ان تتوقف الرضاعة الطبيعية؟	6 أشهر<br>12 أشهر (سنه واحده)<br>18 شهرا (1 سنه ونصف)<br>2 سنوات أو أكثر<br>لا أعرف	
304F	في اي سن ينبغي إدخال الرضيع إلى التغذية التكميلية؟	اقل من 4 أشهر<br>4 – 6 أشهر<br>6 – 8 أشهر<br>أكثر من 8 أشهر<br>لا أعرف	
305F	هل ينبغي ان ينظر إلى الطفل في العينين اثناء الرضاعة؟	نعم<br>لا	
306F	هل يجب إجبار الطفل علي إنهاء الصحن عندما تطعمه؟	نعم<br>لا	
307F	هل يجب ان يتحدث الطفل عندما تطعمه؟	نعم<br>لا	
308F	هل يجب الإسراع بتناول الطعام اثناء الرضاعة؟	نعم<br>لا	
309F	ما الذي يجب ان a الطفل البالغ من العمر 6 أشهر يكون تغذيه؟	الاطعمه اللينة (المهروسة أو هريس)<br>الاطعمه شبه الصلبة<br>الاطعمه العائلية<br>لا أعرف	
310F	كم مره في الأسبوع لا يحتاج الرضيع للاستهلاك اللحوم والدواجن، و/أو الاسماك؟	مره في الأسبوع<br>ثلاث مرات في الأسبوع<br>اليوميه<br>لا يمكنهم أكل هذه	
311F	كم عدد المرات في الأسبوع التي يحتاج فيها الرضيع إلى استهلاك البيض؟	مره في الأسبوع<br>ثلاث مرات في الأسبوع<br>اليوميه<br>لا يمكنهم أكل هذه	
312F	كم مره ينبغي 6a – 8 أشهر الطفل تغذيه في اليوم؟	مره واحده<br>2 -3 مرات<br>4 -5 مرات<br>لا أعرف	
313F	كم مره ينبغي 9 – 11 شهرا الطفل يتم تغذيه في اليوم؟	2 مرات<br>3 – 4 مرات<br>5 – 6 مرات<br>لا أعرف	
314F	متى يمكن للطفل ان ياكلالاطعمه العائلية دون تعديل؟	6 أشهر<br>12 شهرا<br>18 أشهر<br>لا أعرف	

MAKERERE UNIVERSITY
SCHOOL OF HEALTH SCIENCES
APPROVED
VALID UNTIL
01 MAY 2020
RESEARCH & ETHICS COMMITTEE
P. O. BOX 7072, KAMPALA

القسم 4: ممارسات التغذية التكميلية

تعليمات: أسال الاسئله التالية إلى الام (أو مقدم الرعاية) عن طفلهم الذين تتراوح أعمارهم بين 6-23 شهرا. جميع الاسئله تتعلق بذلك الطفل.

Nبني مصفر	الاسئله	استجابه	ملاحظات
401	ما هو عيد ميلاده؟<br><br>إذا كان المجيب لا يعرف تاريخ الميلاد بالبالضبط، أسال: هلهل لديك بطاقة صحية/تطعيم مع تاريخ الميلاد المسجل؟اوالتيthl/بطاقة التطعيم/وثيقة رسميه والمدعي عليه يؤكد صحة المعلومات، وتسجيل تاريخ الميلاد كما هو موثق علي البطاقة. أيضا، تسجيل وزن الولادة إذا ما أشير اليه.		
402	كم شهرا من عمر طفلك؟		
403	التحقق من التناسق (تقويم الاحداث، بطاقة الميلاد)		
404	هل كان طفلكالرضاعة الطبيعية أمس خلال النهار أو في الليل؟		
405a	وبعد ذلك، أود ان اسالك عن بعض السوائل التي قد يكون طفلك قد حصل عليها بالأمس اثناء النهار أو في الليل.<br>وقال انه/لديها اي...:<br><br>ماء عادي؟	نعم<br>لا	
405b	صيغه الرضع مثل نان1, نان2, نوتريشيا،غاليا، الخ. ؟	نعم<br>لا	
405c	الحليب مثل المعلبة،مسحوق، أو الحليب الحيواني الطازج؟	نعم<br>لا	

MAKERERE UNIVERSITY
SCHOOL OF HEALTH SCIENCES
APPROVED
VAL[illegible]
01 MAY 2020
RESEARCH & ETHICS COMMITTEE
P. O. BOX 7072, KAMPALA

405d	عصير أو عصير المشروبات؟	نعم<br>لا	
		نعم<br>لا	
405f	اللبن؟	نعم<br>لا	
405g	عصيدة رقيقه؟	نعم<br>لا	
405h	اي سوائل أخرى؟ تحديد	نعم<br>لا	

MAKERERE UNIVERSITY
SCHOOL OF HEALTH SCIENCES
APPROVED
VALID UNTIL
01 MAY 2020
RESEARCH & ETHICS COMMITTEE
P. O. BOX 7072, KAMPALA

414	يرجى وصف كل ما ياكله طفلك بالأمس خلال النهار أو الليل، سواء في المنزل أو خارج المنزل. لكل وجبه، ضع النقاط النقطية.<br>ا) التفكير في متى (اسم) استيقظ لأول مره أمس. هل أكل (اسم) اي شيء في ذلك الوقت؟ إذا كانت الاجابه بنعم: من فضلك قل لي كل شيء (اسم) أكلت في ذلك الوقت. اي شيء آخر؟ حتى المدعي عليه لا يقول شيئا آخر إذا كان الأمر لا، فتابع السؤال ب).<br>__________<br>__________<br>__________<br>__________<br>__________<br>__________<br>ب) ما الذي فعلاته بعد ذلك؟ هل أكل اي شيء في ذلك الوقت؟<br>إذا كان الجواب نعم: من فضلك قل لي كل شيء انه/انها أكلت في ذلك الوقت. اي شيء آخر؟ حتى المدعي عليه لا يقول شيئا آخر<br>__________<br>__________<br>__________<br>__________<br>كرر السؤال ب) أعلاه حتى يقول المجيب ان الطفل ذهب إلى النوم حتى اليوم التالي.<br>__________<br>__________<br>__________<br>__________

MAKERERE UNIVERSITY
SCHOOL OF HEALTH SCIENCES
APPROVED
VALI[illegible]
01 MAY 2020
RESEARCH & ETHICS COMMITTEE
P. O. BOX 7072, KAMPALA

	______________________________________<br><br>إذا ذكر المجيب الاطباق المختلطة مثل عصيدة، صلصة أو الحساء، والتحقيق:<br><br>ج) ما هي المكونات التي كانت في ذلك (طبق مختلط)؟ اي شيء آخر؟ حتى المدعي عليه لا يقول شيئا آخر<br><br>______________________________________<br>______________________________________<br>______________________________________<br>______________________________________<br>______________________________________<br>______________________________________

القسم 5: المياه والصرف الصحي والمعرفة الصحية

التعليمات: دائره الاستجابات من الخيارات المعطية

Nبني مصفر	الاسئله	استجابه	التعليمات البرمجيه
501F	ما هو صندوق الاماناتسانت مصدر مياه الشرب للأطفال؟	*الأنهار والبحيرات*<br>*أنبوب الآبار*<br>*مياه الامطار*<br>*مياه الصنبور العامة*<br>*لا أعرف*	
502F	ما يجب ان يكون منلا قبل إعطاء الماء للأطفال؟	*شيء*<br>*تسخينه*<br>*يغلي*<br>*اضافه المياه الحرس أو غيرها من وكلاء*	

MAKERERE UNIVERSITY
SCHOOL OF HEALTH SCIENCES
APPROVED
VALID UNTIL
01 MAY 2020
RESEARCH & ETHICS COMMITTEE
P. O. BOX 7072, KAMPALA

		معالجه المياه الامنه<br>لا أعرف	
503F	كيف ينبغي تخزين مياه الشرب؟	تغطيه<br>غير مشمولة<br>غير مغطاة في منطقه نظيفه<br>لا أعرف	
504F	ما الذي يجب ان يكون نظيفهو قبل إطعام طفلك؟	يد مقدم الرعاية فقط<br>ايدي مقدم الرعاية وأيدي الطفل<br>ايدي مقدم الرعاية، وأيدي الطفل والأواني<br>شيء<br>لا أعرف	
505F	Sهولد تغسل يديك بالصدارة اعداد الطعام؟	نعم	
507F	Sهولد تغسل يديك بعد تنظيف قاع الطفل؟	نعم<br>لا	
508F	Sهولد تغسل يديك بعد التغوط؟	نعم<br>لا	

هل هذه التصريحات المتعلقة بنظافة الأطفال مهمة؟

Nبني مصفر	بيان	استجابه	التعليمات البرمجيه
509F	يحتاج الأطفال إلى مساحة نظيفه لتتبع الزحف	نعم<br>لا	
510F	يجب تجنب الذباب والبعوض في المنزل	نعم<br>لا	
511F	الدجاجs لا ينبغي ان تبقي في المناطق التي يلعب فيها الأطفال	نعم<br>لا	
512F	يجب ان ينام الأطفال تحت ناموسيات السرير	نعم<br>لا	
513F	يجب أزاله براز الأطفال بأمان	نعم<br>لا	
514F	إذا كان الجواب نعم، كيف؟		

القسم 6: المياه والصرف الصحي والممارسات الصحية

تعليمات: مراقبه الشيكات الموضعية وتحيط علما وفقا لملاحظه (دائره ما ينطبق).

Nبني مصفر	بقعه	استجابه	التعليمات البرمجيه
601	الام نظيفه	نعم<br>لا	

MAKERERE UNIVERSITY
SCHOOL OF HEALTH SCIENCES
APPROVED
VALID
01 MAY 2020
RESEARCH & ETHICS COMMITTEE
P. O. BOX 7072, KAMPALA

602	تنظيف الأطفال	نعم<br>لا	
603	حفاضات/تنظيف القاع	نعم<br>لا	
604	مجمع نظيف	نعم<br>لا	
605	براز الدواجن في المنزل	نعم<br>لا	
606	براز الطفل في المنزل	نعم<br>لا	
607	المياه الواقفة في المناطق المحيطة	نعم<br>لا	
608	أواني غير مغسولة	نعم<br>لا	
609	مياه الشرب مغطاة	نعم<br>لا	
610	اجتاحت البيت	نعم<br>لا	
611	حاويه القمامة في المنزل<br>اجتاحت البيت	نعم<br>لا	

ال فرع7: المعارف الخاصة بنماء الطفل

التعليمات: دائره ردود من الخيار المعطي.

هل هذه التصريحات المتعلقة بالأطفال مهمة؟

Nبني مصفر	بيان	استجابه	التعليمات البرمجيه
701F	الأمهات بحاجه إلى التحدث مع أطفالهن الرضع	نعم<br>لا	
702F	الأمهات بحاجه إلى اللعب مع أطفالهن مره واحده على الأقل في اليوم	نعم<br>لا	
703F	تحتاج الأمهات إلى قضاء بعض الوقت في أنشطه التعلم مع أطفالهن الرضع	نعم<br>لا	
704F	الاباء بحاجه إلى التحدث مع أطفالهم	نعم<br>لا	

MAKERERE UNIVERSITY
SCHOOL OF HEALTH SCIENCES
APPROVED
VALID [illegible]
01 MAY 2020
RESEARCH & ETHICS COMMITTEE
P. O. BOX 7072, KAMPALA

705F	الاباء بحاجه إلى اللعب مع أطفالهم مره واحده على الأقل في اليوم	نعم<br>لا	
706F	يحتاج الاباء لقضاء بعض الوقت في أنشطه التعلم مع أطفالهم	نعم<br>لا	

القسم 8: تحفيز منزل الطفل

التعليمات: دائره الاستجابات من الخيارات المعطية.

Nبني مصفر	الاسئله	استجابه	التعليمات البرمجيه
801	ويبدو ان الأطفال يطالبون بالاهتمام عندما يكون إباؤهم مشغولين، ويقومون بالعمل المنزلي،على سبيل المثال. هل تستجيب عاده لطلب طفلك على الاهتمام اثناء العمل؟	نعم<br>لا	
802	هل فعل طفلك اي شيء في الأسبوع الأخير الذي يسرك كثيرا؟	نعم<br>لا	
803	إذا كان الجواب نعم،ماذا؟	نعم<br>لا	
804a	ما هي أنواع الأشياء التي تلعبها مع طفلك؟<br>اللعب التي أدلى بها الكبار	نعم<br>لا	
804b	الكائنات المنزلية	نعم<br>لا	
804c	مواد من خارج المنزل	نعم<br>لا	
804d	اللعب التي تجعل الضوضاء	نعم<br>لا	
804e	ألعاب لبناء الأشياء	نعم<br>لا	
804f	لعب للتظاهر مثل الدمى	نعم<br>لا	
804g	غير ذلك (يرجى التحديد)		
805a	في الأسبوع الماضي،على كم من الأيام لم الكبار في الاسره القيام بما يلي مع طفلك؟ (حدد الرقم)<br><br>قراءه الكتب أو إلقاء نظره على الكتب المصورة		

MAKERERE UNIVERSITY
SCHOOL OF HEALTH SCIENCES
APPROVED
VALID ...
01 MAY 2020
RESEARCH & ETHICS COMMITTEE
P. O. BOX 7072, KAMPALA

805b	اخبار القصص		
805c	أغني أغنيات		
805d	الذهاب إلى السوق أو المتجر، أو زيارة خارج المنزل		
805e	اللعب		
805f	قضاء بعض الوقت في أنشطه التعلم مثل تسميه الكائنات		
805g	الجلوس مع الطفل خلال الوجبة الرئيسية من اليوم		
805h	التحدث اثناء الوجبات		
806	كيف تعرفين عندما يكون طفلك جائعا؟<br>دائره كل ما ينطبق	صرخات<br>يسال عن الطعام،والنقاط، أو يستخدم الإيماءات (ولكن لا يبكي)<br>غير ذلك (يرجى التحديد) ____	
807	عندما تخدم طعام طفلك، كيف يتمّ تقديمه؟	وعاء منفصل<br>لوحه الاسره المشتركة أو المشتركة<br>لم يبدا الطفل في تناول الاطعمه الأخرى	
808	ماذا تفعل عاده للحصول على طفلك للأكل؟<br>دائره كل ما ينطبق	شيء<br>أخبر الطفل ان ياكل<br>تشجيع،الثناء، اللعب أو عقد<br>إعطاء أنواع أخرى من المواد الغذائية<br>القوة أو التهديد أو الضرب<br>غير ذلك (يرجى التحديد) ___	

القسم 9: مشاركه الأب في رعاية الطفل

التعليمات: دائره الاستجابات من الخيارات المعطية

كم مره زوجك/شريكك...؟

Nبني مصفر	الاسئله	استجابه	التعليمات البرمجيه

MAKERERE UNIVERSITY
SCHOOL OF HEALTH SCIENCES
APPROVED
VALID UNTIL
01 MAY 2020
RESEARCH & ETHICS COMMITTEE
P. O. BOX 7072, KAMPALA

901	المساهمة بالمال لدعم الطفل بانتظام، ودفع ثمن الطعام	ابدا<br>احيانا<br>دائما تقريبا	
903	خذ الطفل إلى مركز الرعاية الصحية منذ ولادته، وحده أو معك	ابدا<br>احيانا<br>دائما تقريبا	
903	اللعب والتحدث مع الطفل يوميا	ابدا<br>احيانا<br>دائما تقريبا	
904	أطعم واعتني بالطفل يوميا تقريبا	ابدا<br>احيانا<br>دائما تقريبا	
905	عقد وحمل الطفل يوميا	ابدا<br>احيانا<br>دائما تقريبا	
905	تعليم الأشياء للطفل	ابدا<br>احيانا<br>دائما تقريبا	
906	اعتني بالطفل عندما تكون مشغولا	ابدا<br>احيانا<br>دائما تقريبا	
907	نصيحة لك بشأن الأمور المتعلقة بالطفل	ابدا<br>احيانا<br>دائما تقريبا	

القسم 10: الصحة العقلية

.التعليمات: دائره الاستجابات من الخيارات المعطية

Nبني مصفر	الاسئله	استجابه	التعليمات البرمجيه
1001	هل غالبا ما يكون لديك صداع؟	نعم<br>لا	

MAKERERE UNIVERSITY
SCHOOL OF HEALTH SCIENCES
APPROVED
VALID UNTIL
01 MAY 2020
RESEARCH & ETHICS COMMITTEE
P. O. BOX 7072, KAMPALA

1002	هل شهيتك ضعيفه؟	نعم<br>لا	
1003	هل تنام بشكل سيء؟	نعم<br>لا	
1004	هل أنت خائف بسهوله؟	نعم<br>لا	
1005	هل تهز يديك؟	نعم<br>لا	
1006	هل تشعر بالعصبية أو التوتر أو القلق؟	نعم<br>لا	
1007	هل الهضم الخاص بك الفقراء؟	نعم<br>لا	
1008	هل لديك مشكله في التفكير بوضوح؟	نعم<br>لا	
1009	هل تشعر بالتعاسة؟	نعم<br>لا	
1010	هل تبكي أكثر من المعتاد؟	نعم<br>لا	
1011	هل تجد صعوبة في الاستمتاع بأنشطكاليومية؟	نعم<br>لا	
1012	هل تجد صعوبة في اتخاذ القرارات؟	نعم<br>لا	
1013	هل عملك اليومي يعاني؟	نعم<br>لا	
1014	هل أنت غير قادر علي لعب دور مفيد في الحياة؟	نعم<br>لا	
1015	هل فقدت الاهتمام بالأشياء؟	نعم<br>لا	
1016	هل تشعر بانك شخص عديم القيمة؟	نعم<br>لا	
1017	هل تشعر بالتعب طوال الوقت؟	نعم<br>لا	

MAKERERE UNIVERSITY
SCHOOL OF HEALTH SCIENCES
APPROVED
VALID [illegible]
01 MAY 2020
RESEARCH & ETHICS COMMITTEE
P. O. BOX 7072, KAMPALA

	نعم<br>لا	هل أنت متعب بسهوله؟	1018
	نعم<br>لا	هل لديك مشاعر غير مريحه في معدتك؟	1019

الفرع 11: الدعم الاجتماعي

التعليمات: دائره الاستجابات من الخيارات المعطية.

التعليمات البرمجيه	استجابه	الاسئله	Nبني مصفر
	الام أو الام في القانون<br>الزوج<br>الإخوة والأخوات<br>صديق<br>غير ذلك (يرجى التحديد)	من الذي يدعمك بشكل عام في حياتك؟	1101
	عفوًا<br>نادرا ما<br>بطريقه ما<br>معظمها<br>بقدر ما I مثل	انا بزيارة مع الأصدقاء والأقارب	1102
	عفوًا<br>نادرا ما<br>بطريقه ما<br>معظمها<br>بقدر ما I مثلمثل	احصل على مساعده في جميع انحاء المنزل	1103
	عفوًا<br>نادرا ما<br>بطريقه ما<br>معظمها<br>بقدر ما I مثلمثل	احصل على مساعده بالمال في حاله الطوارئ	1104
	عفوًا<br>نادرا ما<br>بطريقه ما<br>معظمها<br>بقدر ما I مثلمثل	انا الحصول على الثناء على عمل جيد القيام به/الانتهاء	1105

MAKERERE UNIVERSITY
SCHOOL OF HEALTH SCIENCES
APPROVED
VALID UNTIL
01 MAY 2020
RESEARCH & ETHICS COMMITTEE
P. O. BOX 7072, KAMPALA

1106	لدي أشخاص يهتمون بما يحدث لي	عفوًا<br>نادرا ما<br>بطريقه ما<br>معظمها<br>بقدر ما I مثلمثل	
1107	احصل على الحب والمودة المطلوبين بشده	عفوًا<br>نادرا ما<br>بطريقه ما<br>معظمها<br>بقدر ما I مثلمثل	
1108	احصل على مكالمات هاتفيه من أشخاص اعرفهم	عفوًا<br>نادرا ما<br>بطريقه ما<br>معظمها<br>بقدر ما I مثلمثل	
1109	احصل على زيارة الناس للاطمئنان على لي ومعرفه ما إذا كنت بخير	عفوًا<br>نادرا ما<br>بطريقه ما<br>معظمها<br>بقدر ما I مثلمثل	
1110	احصل على فرص للتحدث مع شخص ما حول المشاكل في العمل أو مع الاعمال المنزلية بلدي	عفوًا<br>نادرا ما<br>بطريقه ما<br>معظمها<br>بقدر ما I مثلمثل	
1111	احصل على فرص للتحدث مع شخص أثق به في مشاكلي الشخصية	عفوًا<br>نادرا ما<br>بطريقه ما<br>معظمها<br>بقدر ما I مثلمثل	
1112	احصل على فرص للحديث عن المسائل المالية	عفوًا<br>نادرا ما<br>بطريقه ما<br>معظمها<br>بقدر ما I مثلمثل	

MAKERERE UNIVERSITY
SCHOOL OF HEALTH SCIENCES
APPROVED
VALID UNTIL
01 MAY 2020
RESEARCH & ETHICS COMMITTEE
P. O. BOX 7072, KAMPALA

1113	احصل على دعوات للخروج والقيام بأشياء مع أشخاص آخرين	عفوًا<br>نادرا ما<br>بطريقه ما<br>معظمها<br>بقدر ما I مثلمثل	
1114	احصل علىنصيحةمفيده حول الأشياء الهامة في الحياة	عفوًا<br>نادرا ما<br>بطريقه ما<br>معظمها<br>بقدر ما I مثلمثل	
1115	احصل على المساعدة عندما احتجت للنقل	عفوًا<br>نادرا ما<br>بطريقه ما<br>معظمها<br>بقدر ما I مثلمثل	
1116	احصل على المساعدة عندما أكون مريضا في السرير	عفوًا<br>نادرا ما<br>بطريقه ما<br>معظمها<br>بقدر ما I مثلمثل	
1117	هل لديك اي شخص يمكنك اللجوء اليه للحصول على اقتراحات حول كيفية التعامل مع مشكله عائليه؟	عفوًا<br>نادرا ما<br>بطريقه ما<br>معظمها<br>بقدر ما I مثلمثل	
1118	احصل على المساعدة في الاعمال اليومية عندما أكون مريضا؟	عفوًا<br>نادرا ما<br>بطريقه ما<br>معظمها<br>بقدر ما I مثلمثل	
1119	لدي شخص يمكنني الحصول على وقت جيد معه؟	عفوًا<br>نادرا ما<br>بطريقه ما<br>معظمها<br>بقدر ما I مثلمثل	

MAKERERE UNIVERSITY
SCHOOL OF HEALTH SCIENCES
APPROVED
VALID UNTIL
01 MAY 2020
RESEARCH & ETHICS COMMITTEE
P. O. BOX 7072, KAMPALA

نماء الطفل (6 أشهر)

التعليمات: دائره الاستجابات من الخيارات المعطية.

كم مره لاحظت هذه...؟

Nبني مصفر	الاسئله	استجابه	التعليمات البرمجيه
	هل يصنع طفلك ممسحة عاليهالنبرة؟	نعم<br>لا	
	عند اللعب مع الأصوات، هل طفلك يجعل الشخير،الهدر، أو غيرها من الأصوات العميقة منغم؟	نعم<br>لا	
	إذا اتصلت بطفلك عندما تكون بعيدا عن الأنظار، هل تنظر في اتجاه صوتك؟	نعم<br>لا	
	عندما تحدث ضوضاء عاليه، هل يتحول طفلك لرؤية من أين جاء الصوت؟	نعم<br>لا	
	هل طفلك يجعل الأصوات مثل "دا"، "ga"، "كا" و "با" ؟	نعم<br>لا	
	إذا قمت بنسخ الأصوات التي يصنعها طفلك، هل يعيد طفلك نفس الأصوات اليك؟	نعم<br>لا	
	في حين ان طفلك علىظهره، هل يرفع طفلك ساقه بدرجه كافيه لرؤية قدميه؟	نعم<br>لا	
	عندما يكون طفلك علىبطنها، هل يقوم بتصويب الذراعين ودفع صدره كله من السرير أو الأرض؟	نعم<br>لا	
	هل لفه طفلك من ظهره إلى البطن له/لها، والحصول على كل من الذراعين من تحت له/لها/	نعم<br>لا	
	عند وضع طفلك علىالأرض، هل هو/هي العجاف على يديه/لها اثناء الجلوس؟ (إذا كان يجلس بالفعل على التوالي دون ان يميل علىيديها، علامة "نعم")	نعم<br>لا	
	إذا كنت تحمل كلتا اليدين فقط لتحقيق التوازن بين طفلك، فهل يدعم وزنه الخاص اثناء الوقوف؟	نعم<br>لا	
	هل يدخل طفلك في وضعيه الزحف عن طريق الاستيقاظ على يديه وركبته؟	نعم<br>لا	
	هل يقوم طفلك بالإمساك باللعبة التي تقدمها والنظر اليها أو التلويح بها أو مضغها لمده دقيقه واحده؟	نعم<br>لا	

MAKERERE UNIVERSITY
SCHOOL OF HEALTH SCIENCES
APPROVED
VALID UNTIL
01 MAY 2020
RESEARCH & ETHICS COMMITTEE
P. O. BOX 7072, KAMPALA

	نعم<br>لا	إذا قمت بنسخ الأصوات التي يصنعها طفلك، هل يعيد طفلك نفس الأصوات اليك؟	
	نعم<br>لا	هل يصنع طفلك صوتين متشابهين مثل "بابا" أو "دا-دا" أو "ga-ga"؟ (الصوت لا يعني اي شيء)	
	نعم<br>لا	إذا سالت طفلك، هل يلعب لعبه حضانة واحده على الأقل حتى لو لم تظهر له النشاط بنفسك (على سبيل المثال: "وداعا"، "بييكابو"، "صفق بيديك"، الخ)	
	نعم<br>لا	هل يتبع طفلك أمر عينه واحده، مثل "تعال هنا"، "أعطه لي"، أو "أعده"، دون استخدام الإيماءات؟	
	نعم<br>لا	هل يقول طفلك ثلاث كلمات، مثل "ماما"، "دادا"، و "بابا" (a "كلمه" هو صوت أو أصوات طفلك يقول باستمرار يعني شخص ما أو شيء من هذا القبيل)	
	نعم<br>لا	إذا كنت تحمل كلتا اليدين فقط لتحقيق التوازن بين طفلك، فهل يدعم وزنه الخاص اثناء الوقوف؟	
	نعم<br>لا	عندما تجلس علىالأرض، هل الطفل الخاص بك الجلوس على التوالي لعده دقائق دون استخدام يديه/لها للحصول علىالدعم؟	
	نعم<br>لا	عندما كنت حاملا طفلك بجانب الأثاث أو السكك الحديدية سرير، فهل تمسك بدون يميل صدرها ضد الأثاث للحصول علىالدعم؟	
	نعم<br>لا	بينما التمسك الأثاث، هل طفلك ينحني والتقاط لعبه من الأرض ومن ثم العودة إلى موقف دائم؟	
	نعم<br>لا	بينما التمسك الأثاث، هل طفلك اقل نفسه/نفسها مع السيطرة (دون السقوط أو التخبط)؟	
	نعم<br>لا	هل يمشي طفلك بجانب الأثاث اثناء الإمساك بيد واحده فقط؟	
	نعم<br>لا	هل يلتقط طفلك لعبه صغيره بيد واحده فقط؟	
	نعم<br>لا	هل طفلك يلتقط بنجاح فتات باستخدام الإبهام له/لها وجميع أصابعه/لها في حركه التكتل (إذا كان/انها تلتقط بالفعل كسره خبز، علامة "نعم")	
	نعم<br>لا	هل يلتقط طفلك لعبه صغيره مع نصائح إبهامه وأصابعه؟	
	نعم<br>لا	بعد محاولة واحده أو اثنتين، هل يلتقط طفلك قطعه من الخيط مع اصبعه الأول وإبهامه؟	
	نعم<br>لا	هل يلتقط طفلك فتات الخبز مع نصائح إبهامه واصبعه؟ وقال انه قد يستريح له/لها الذراع أو اليد على الطاولة اثناء القيام بذلك	

MAKERERE UNIVERSITY
SCHOOL OF HEALTH SCIENCES
APPROVED
VALID UNTIL
01 MAY 2020
RESEARCH & ETHICS COMMITTEE
P. O. BOX 7072, KAMPALA

	نعم<br>لا	هل وضع طفلك لعبه صغيره أسفل، دون ان تسقط، ومن ثم تاخذ يدها من لعبه؟	
	نعم<br>لا	بينما طفلك في ظهره، هل وضعت قدمها في فمه؟	
	نعم<br>لا	هل يشرب طفلك الماء أو العصير من الكوب اثناء الحمل؟	
	نعم<br>لا	هل يطعم طفلك نفسه كعكه؟ (أو شيء من هذا القبيل)	
	نعم<br>لا	عندما كنت تحمل يدك ونسأل عن له/لها لعبه، هل يقدم لك طفلك حتى إذا كان/انها لا تدع الذهاب منه؟ (إذا كانت بالفعل دعوانا نذهب من لعبه في يدك، علامة "نعم")	
	نعم<br>لا	عندما تلبسين طفلك، هل يدفع ذراعه من خلال الأكمام بمجرد ان يبدا ذراعه في ثقب الأكمام؟	
	نعم<br>لا	عندما تمسك يدك وتسال عن لعبتها، هل يترك طفلك الأمر في يدك؟	

MAKERERE UNIVERSITY
SCHOOL OF HEALTH SCIENCES
APPROVED
VALID UNTIL
01 MAY 2020
RESEARCH & ETHICS COMMITTEE
P. O. BOX 7072, KAMPALA

مناقشات مجموعه التركيز ومقابلات المخبر الرئيسي

مجموعات النظراء لتحسين ممارسات التغذية وتغذيه الأطفال في المستوطنات التي تلي حالات الطوارئ في منطقه غرب النيل في أوغندا.

مناقشات مجموعه الأمهات التركيز

ممارسات الدعم الاجتماعي

1. من يعتني بالأم والمولود الوليد بعد ولادتها؟ متابعه السؤال: كم من الوقت بعد الولادة هل الام البقاء في المنزل مع الطفل؟
2. هل من المهم بالنسبة لك ان تكون قادرا على التفاعل مع الأصدقاء أو العائلة أو مجموعات أخرى من الناس؟
3. هل هناك أشياء عائلتك أو أصدقائك القيام به، أو يمكن ان تفعله بالنسبة لك لتجعلك تشعر بأنها مهمة أو مفيدهلحياتك؟
4. إذا كان لديك اي مخاوف/مشاكل عميقة، من الذي كنت تعتمد علي؟ توضيح السبب؟
5. إذا كنت ترغب في التقاء أو زيارة أشخاص آخرين، هل أنت قادر علي القيام بذلك بسهوله؟ وإذا لم يكن الأمر كذلك، فما الذي يجعل من الصعب القيام بذلك؟
6. هل تشعر بانك تفهم من قبل الأصدقاء والعائلة؟ ما مدي اهميه ذلك بالنسبة لك ان كنت فهمت؟

ممارسات تغذيه الرضع وصغار الأطفال

1. إذا كان لديك اسئله حول الرضاعة الطبيعية طفلك، الذي كنت أسال؟ يرجى التفكير في جميع الناس يمكنك الذهاب إلى للحصول على المشورة.
2. في مجتمعك، ما هي تغذيه الرضع بعد الولادة مباشره؟ من يطعم الرضع بعد الولادة مباشره؟ المتابعة: هل هذه ممارسه شائعه؟
3. عموما، ما الذي قد تفكر به عند إطعام طفلك اقل من سته أشهر؟
4. ما رأيك من أجل إعطاء طفلك الاطعمهالتكميلية؟ متى يمكنهم البدء؟ ما هي الاطعمه التي يمكنك إطعام طفل صغير (ربما نسأل قبل ان يتمكنوا من المشي؟) هل سمعت شيئا عن الاطعمه التي لا يمكن للطفل ان ياكلها؟ (المسبار تناول البيض أو غيرها من المواد الغذائية مصدر الحيوانية)
5. في منزلك، ما هو ترتيب الاولويه لتقديم وجبات الطعام؟ لم؟ في اي سن سيكون الطفل أولا تذوق الطعام العائلي العادي؟
6. كيف تطعمني) اقل من سته أشهر طفل مريض؟
   a. سؤال المتابعة: 2) أكثر من سته أشهر طفل مريض؟
7. كيف يؤثر الناس وبيئتك علىكيفية إطعام طفلك؟
8. كم عدد المرات التي تطبخ فيها الاغذيه التكميلية في اليوم الواحد؟

MAKERERE UNIVERSITY
SCHOOL OF HEALTH SCIENCES
APPROVED
VALID UNTIL
01 MAY 2020
RESEARCH & ETHICS COMMITTEE
P. O. BOX 7072, KAMPALA

مشاركه الأب في تغذيه الأطفال ورعايتهم

1. هل يقوم زوجك بأنشطه تساهم في تغذيه الأطفال؟ (التحقيق للحصول على أمثله)
2. إذا كان الزوج/الشريك لا يشارك في الانشطه الرامية إلى تغذيه الطفل، لماذا تعتقد ان هذا هو الحال؟
3. كيف تشعر إذا كان شريكك/الزوج يساعد في القيام بالاعمالالمنزلية؟
4. كيف تفضل ان يدعمك زوجك/شريكك في الاعمال المنزلية (التحقيق نحو تغذيه الطفل)؟
5. كيف سيشعر زوجك بالتحدث إلى الأصدقاء أو افراد عائلتك أو الانضمام إلى مجموعه اجتماعيه في مجتمعك؟

مناقشات مجموعه الاباء التركيز

ات الدعم الاجتماعي

1. كم من الوقت بعد الولادة هل الام البقاء في المنزل مع الطفل؟
2. ماذا ستفعل لزوجتك لتجعلها تشعر بانك مهم أو مفيد لحياتها؟
3. هل تعتقد انه من المهم لزوجتك ان تتفاعل مع الأصدقاء أو العائلة أو مجموعات أخرى من الناس؟
4. كيف تشعر بشأن زوجتك تقاسم اي مخاوف عميقة/مشاكل مع غيرهم من الناس موثوق بها؟ شرح لماذا
5. كيف تشعر حيال اجتماع زوجتك أو زيارة أشخاص آخرين؟
6. من يعتني بالأم والمولود الوليد بعد ولادة الام؟
7. كيف يؤثر الناس وبيئتك علىكيفية إطعام طفلك؟

ممارسات تغذيه الرضع وصغار الأطفال

1. متى ينبغي ان تبدأ التغذية التكميلية للطفل؟ ما الذي يجب تغذيته؟
2. .إذا كان لديك اسئله حول التغذية التكميلية لطفلك الذي تسال؟ يرجى إعطاء أكبر قدر ممكن من التفاصيل
3. في منزلك، ما هو ترتيب الاولويه لتقديم وجبات الطعام؟ لم؟
4. في منزلك، من يقرر ما الذي يتغذى عليه الطفل؟ لم؟
5. بشكل عام، ما هي الأمور التي قد تجعل من الصعب على زوجتك ان ترضع طفلك بشكل حصري حتى يبلغ من العمر ستة أشهر؟
6. هل هناك أسباب لأنه سيكون من الصعب البدء في إطعام الأطفال الرضع المكملات الغذائية الخاصة بك عندما تكون سته أشهر من العمر؟ هل سمعت عن الاطعمه التي لا ينبغي للطفل ان ياكل؟ ما هي الأسباب؟
7. اقل من سته أشهر طفل مريض؟ a1 كيف يمكنك إطعام
   a. *سؤال المتابعة: 2 أكثر من سته أشهر طفل مريض؟*
8. إذا كنت تريد ان تكون أكثر مشاركه في إطعام طفلك، هل سيكون من الصعب القيام بذلك؟ لماذا أو لماذا لا؟

مشاركه الأب في تغذيه الأطفال ورعايتهم

1. هل تساهم في تغذيه الأطفال؟ ماذا تفعل؟
2. كيف تشعر حيال التورط في إطعام الأطفال؟
3. كيف تشعر حيال المساعدة في الاعمال المنزلية؟
4. هل هناك طريقه يمكن للزوج ان يدعم زوجته بالاعمالالمنزلية؟ (التحقيق نحو تغذيه الطفل،والاجوبه التفصيلية)؟

MAKERERE UNIVERSITY
SCHOOL OF HEALTH SCIENCES
APPROVED
VALID
01 MAY 2020
RESEARCH & ETHICS COMMITTEE
P. O. BOX 7072 KAMPALA

5. ما هو شعورك تجاه زوجتك التي تتحدث إلى الأصدقاء أو افراد العائلة أو المجموعات الاجتماعية الأخرى في مجتمعك؟

MAKERERE UNIVERSITY
SCHOOL OF HEALTH SCIENCES
APPROVED
VALID UNTIL
01 MAY 2020
RESEARCH & ETHICS COMMITTEE
P. O. BOX 7072, KAMPALA

مقابلات المخبر الرئيسية

ممارسات الدعم الاجتماعي

1. إذا كان الاباء والأمهات لديهم مخاوف عميقة أو مشاكل، الذين يعتمدون على أو الذهاب للحصول علىالمساعدة؟
2. في مجتمعك، ما هي تغذيه الرضع بعد الولادة مباشره؟ من يطعم الرضع بعد الولادة مباشره؟ المتابعة: هل هذه ممارسه شائعه؟
3. من يعتني بالأم والمولود الوليد بعد الولادة قد حدث؟
4. هل من السهل أو الصعب على الاباء الاعتماد على أشخاص آخرين للحصول علىالمساعدة؟ (التحقيق في المالية والاجتماعية ورعاية الطفل،الخ) لم؟
5. هل ينخرط الاباء في مجتمعك في الاعمال المنزلية وتغذيه الأطفال؟

ممارسات تغذيه الرضع وصغار الأطفال

1. إذا كان الاباء لديهم اسئله حول ممارسات تغذيه الأطفال، فمن سيسألون؟
2. في الاسرهالمعيشية، الذي عاده ما يقدم أو يجلب أو يشتري الطعام؟
3. في الاسرهالمعيشية، ما هو الأمر الذي يحظى بالاولويه بين افراد العائلة لتقديم وجبات الطعام في منزل؟ لم؟
4. في الاسره المعيشية التي تحضر الطعام عاده للأكل؟ الذي عاده ما يقرر ما لإعطاء الطفل؟
5. هل هناك شيء من شانه ان يجعل من الأسهل للإباء والأمهات لتحسين ما ياكلأطفالهم؟ ما الذي يجعل الأمر صعبا؟

مشاركه الأب في ممارسات تغذيه الأطفال

1. ما الذي ستقوله النساء وغيرهن من أعضاء المجتمع إذا كان الاباء يرون الطعام؟ إطعام طفلهم؟ ماذا سيقول الأصدقاء الذكور؟
2. ما الذي سيقوله أعضاء المجتمع إذا كان الاباء يرون يقومون باعمالمنزليه؟
3. وإذا كان الاباء لا يشاركون في أنشطه تغذيه الأطفال والاعمال المنزلية، فهل هذه هي الأسباب؟
4. في المجتمع، هل هناك الاطعمه التي سمعت لا ينبغي إطعامها للأطفال؟ إذا كان الأمر كذلك، ما هي تلك الاطعمه؟
5. ما هي الاطعمه التي تتغذي عاده على الرضع لأول أغذيهتكميليه؟

الوصول إلى المجتمع

1. ما هي الطريقة الأكثر فعاليه للوصول إلى/اشراك الأمهات؟
2. ما هي الطريقة الأكثر فعاليه للوصول إلى/اشراك الاباء؟
3. هل العديد من الأسر المعيشية لديها عائلتها الممتدة معهم؟
4. ما هي الطريقة الأكثر فعاليه لتعليم الأمهات؟
5. هل تعمل معظم العائلات على الهواتف المحمولة؟ احتفظ بها من قبل الأب أو الام؟ هل هناك رسوم لتلقي الرسائل النصية؟
6. هل هناك محطه إذاعيه محليه تبث في الدينكا أو العربية؟
7. هل هناك جدول زمني للحملات الصحية الحكومية؟
8. في تجربتك، ما هي الصعوبات التي يجب ان يتجنبها فريق البحث لدينا في التفاعل مع المجتمع

MAKERERE UNIVERSITY
SCHOOL OF HEALTH SCIENCES
APPROVED
VALID UNTIL
01 MAY 2020
RESEARCH & ETHICS COMMITTEE
P. O. BOX 7072, KAMPALA

HHID__________ FATHERID__________

مجموعات النظراء لتحسين ممارسات التغذية والحد من سوء التغذية بين الأطفال اللاجئين في المستوطنات التي تلي حالات الطوارئ في أوغندا.

استبيان الاباء

معلومات حول استبيان الاستطلاع

ملاحظه	المسؤوله عن	نهاية الوقت	بداية الوقت	تاريخ	المهمه
					الدراسهالاستقصائيه
					إدخال البيانات
					تاكيد إدخال البيانات

معلومات عن الاسره

معرف	اسم	البند
		المقابله
		ادجوماني
		قرية
		الفوج
		الهوية المنزلية
		الاب
		الطفل

عند الانتهاء من المسح، يرجى أزاله هذه الصفحة الاولي والاحتفاظ بها حيث في المربع مقفلة لضمان عدم الكشف عن هويه الاسره المعيشية.

MAKERERE UNIVERSITY
SCHOOL OF HEALTH SCIENCES
APPROVED
VALID UNTIL
01 MAY 2020
RESEARCH & ETHICS COMMITTEE
P. O. BOX 7072, KAMPALA

HHID________ FATHERID__________

القسم 1: المعارف التكميلية للتغذية

التعليمات: دائره ردود من الخيار المعطي.

Nبني مصفر	الاسئله	استجابه	التعليمات البرمجيه
301F	أين تلقيت المعلوماتيةأيون عن التغذية التكميلية؟ (دائره كل ما ينطبق)	العاملون في الصحة/المجتمع<br>افراد الاسره<br>وسائل الاعلام<br>الاخري<br>مكان	
302F	بخلاف حليب الثدي، ما الرضع ان يكون اعطاءn خلال الأشهر الستة الاولي؟	الماء الساخن<br>المياه السكرية<br>لا شيء، حليب الثدي فقط<br>*الاطعمه الطرية*<br>*لا أعرف*	
303F	متى يجب ان تتوقف الرضاعة الطبيعية؟	*6 أشهر*<br>*12 أشهر (سنه واحده)*<br>*18 شهرا (1 سنه ونصف)*<br>*2 سنوات أو أكثر*<br>*لا أعرف*	
304F	في اي سن ينبغي إدخال الرضيع إلى التغذية التكميلية؟	*اقل من 4 أشهر*<br>*4 – 6 أشهر*<br>*6 – 8 أشهر*<br>*أكثر من 8 اشهر*<br>*لا أعرف*	
305F	هل ينبغي ان ينظر إلى الطفل في العينين اثناء الرضاعة؟	نعم<br>لا	
306F	هل يجب إجبار الطفل علي إنهاء الصحن عندما تطعمه؟	نعم	

MAKERERE UNIVERSITY
SCHOOL OF HEALTH SCIENCES
APPROVED
VALID [illegible]
01 MAY 2020
RESEARCH & ETHICS COMMITTEE
P. O. BOX 7072, KAMPALA

		لا	
307F	هل يجب ان يتحدث الطفل عندما تطعمه؟	نعم<br><br>لا	
308F	هل يجب الإسراع بتناول الطعام اثناء الرضاعة؟	نعم<br><br>لا	
309F	ما الذي يجب ان a الطفل البالغ من العمر 6 أشهر يكون تغذيه؟	الاطعمه اللينة (المهروسة أو هريس)<br><br>الاطعمه شبه الصلبة<br><br>الاطعمه العائلية<br><br>لا أعرف	
310F	كم مره في الأسبوع لا يحتاج الرضيع للاستهلاك اللحوم والدواجن، و/أو الاسماك؟	مره في الأسبوع<br><br>ثلاث مرات في الأسبوع<br><br>اليوميه<br><br>لا يمكنهم أكل هذه	
311F	كم عدد المرات في الأسبوع التي يحتاج فيها الرضيع إلى استهلاك البيض؟	مره في الأسبوع<br><br>ثلاث مرات في الأسبوع<br><br>اليوميه<br><br>لا يمكنهم أكل هذه	
312F	كم مره ينبغي 6a – 8 أشهر الطفل تغذيه في اليوم؟	مره واحده<br><br>2 -3 مرات<br><br>4 -5 مرات<br><br>لا أعرف	
313F	كم مره ينبغي 9 – 11 شهرا الطفل يتم تغذيه في اليوم؟	2 مرات<br><br>3 – 4 مرات<br><br>5 – 6 مرات<br><br>لا أعرف	
314F	متى يمكن للطفل ان ياكلالاطعمه العائلية دون تعديل?	6 أشهر<br><br>12 شهرا	

MAKERERE UNIVERSITY
SCHOOL OF HEALTH SCIENCES
APPROVED
VALID
01 MAY 2020
RESEARCH & ETHICS COMMITTEE
P. O. BOX 7072, KAMPALA

		18 أشهر<br>لا أعرف	

MAKERERE UNIVERSITY
SCHOOL OF HEALTH SCIENCES
APPROVED
VALID [illegible]
01 MAY 2020
RESEARCH & ETHICS COMMITTEE
P. O. BOX 7072, KAMPALA

HHID________ FATHERID__________

الباب 2: المياه والصرف الصحي والمعرفة الصحية

التعليمات: دائره الاستجابات من الخيارات المعطية

Nبني مصفر	الاسئله	استجابه	التعليمات البرمجيه
501F	ما هو صندوق الاماناتسانت مصدر مياه الشرب للأطفال؟	*الأنهار والبحيرات*<br>*أنبوب الآبار*<br>*مياه الامطار*<br>*مياه الصنبور العامة*<br>*لا أعرف*	
502F	ما يجب ان يكون منلا قبل إعطاء الماء للأطفال؟	شيء<br>تسخينه<br>*يغلي*<br>*اضافه المياه الحرس أو غيرها من وكلاء معالجه المياه الامنه*<br>*لا أعرف*	
503F	كيف ينبغي تخزين مياه الشرب؟	*تغطيه*<br>غير مشمولة<br>غير *مغطاة في منطقه نظيفه*<br>*لا أعرف*	
504F	ما الذي يجب ان يكون نظيفهو قبل إطعام طفلك؟	يد مقدم *الرعاية فقط*<br>ايدي مقدم *الرعاية وأيدي الطفل*<br>ايدي مقدم *الرعاية، وأيدي الطفل والأواني*<br>شيء<br>*لا أعرف*	
505F	Sهولد تغسل يديك بالصدارة اعداد الطعام؟	نعم<br>لا	
507F	Sهولد تغسل يديك بعد تنظيف قاع الطفل؟	نعم	

MAKERERE UNIVERSITY
SCHOOL OF HEALTH SCIENCES
APPROVED
VALID [illegible]
01 MAY 2020
RESEARCH & ETHICS COMMITTEE
P. O. BOX 7072, KAMPALA

		لا	
508F	Sهولد تغسل يديك بعد التغوط؟	نعم<br>لا	

هل هذه التصريحات المتعلقة بنظافة الأطفال مهمة؟

Nبني مصفر	بيان	استجابه	التعليمات البرمجيه
509F	يحتاج الأطفال إلى مساحة نظيفه لتتبع الزحف	نعم<br>لا	
510F	يجب تجنب الذباب والبعوض في المنزل	نعم<br>لا	
511F	الدجاجs لا ينبغي ان تبقي في المناطق التي يلعب فيها الأطفال	نعم<br>لا	
512F	يجب ان ينام الأطفال تحت ناموسيات السرير	نعم<br>لا	
513F	يجب أزاله براز الأطفال بأمان	نعم<br>لا	
514F	إذا كان الجواب نعم،كيف؟		

الفرع 3: المعارف الخاصة بنماء الطفل

التعليمات: دائره ردود من الخيار المعطي.

هل هذه التصريحات المتعلقة بالأطفال مهمة؟

Nبني مصفر	بيان	استجابه	التعليمات البرمجيه
701F	الأمهات بحاجه إلى التحدث مع أطفالهن الرضع	نعم	

MAKERERE UNIVERSITY
SCHOOL OF HEALTH SCIENCES
APPROVED
VA[illegible]
01 MAY 2020
RESEARCH & ETHICS COMMITTEE
P. O. BOX 7072, KAMPALA

			لا	
702F	الأمهات بحاجه إلى اللعب مع أطفالهن مره واحده على الأقل في اليوم		نعم<br>لا	
703F	تحتاج الأمهات إلى قضاء بعض الوقت في أنشطه التعلم مع أطفالهن الرضع		نعم<br>لا	
704F	الاباء بحاجه إلى التحدث مع أطفالهم		نعم<br>لا	
705F	الاباء بحاجه إلى اللعب مع أطفالهم مره واحده على الأقل في اليوم		نعم<br>لا	
706F	يحتاج الاباء لقضاء بعض الوقت في أنشطه التعلم مع أطفالهم		نعم<br>لا	

القسم 4: الدعم الاجتماعي

التعليمات: دائره الاستجابات من الخيار المعطي.

Nبني مصفر	الاسئله	استجابه	التعليمات البرمجيه
1101	من ان دعم عموما الامهات الأكثر في هذه الحياة؟	الام أو الام في القانون<br>الزوج<br>الإخوة والأخوات<br>صديق<br>غير ذلك (يرجى التحديد)	
1102	يجب على الأمهات زيارة الأصدقاء والأقارب	عفوًا<br>نادرا ما<br>بطريقه ما<br>معظمها	

MAKERERE UNIVERSITY
SCHOOL OF HEALTH SCIENCES
APPROVED
VALID UNTIL
01 MAY 2020
RESEARCH & ETHICS COMMITTEE
P. O. BOX 7072, KAMPALA

HHID__________ FATHERID____________

		بقدر ما تحب	
1103	يجب ان يساعد الاباء في جميع انحاء المنزل	عفوًا<br>نادرا ما<br>بطريقه ما<br>معظمها<br>بقدر ما تحب	
1104	يجب على الاباء دعم الأمهات بالمال في حاله الطوارئ	عفوًا<br>نادرا ما<br>بطريقه ما<br>معظمها<br>بقدر ما تحب	
1105	Mothers should get praise for a good job done/completed<br>يجب على الأمهات الحصول على الثناء لعمل جيد القيام به/الانتهاء	عفوًا<br>نادرا ما<br>بطريقه ما<br>معظمها<br>بقدر ما تحب	
1106	يجب على الاباء إعطاء الحب والمودة لزوجهاتهم	عفوًا<br>نادرا ما<br>بطريقه ما<br>معظمها<br>بقدر ما تحب	
1107	يجب ان تكون الأمهات قادرات على الحصول على مكالمات هاتفيه من الأشخاص الذين يفحصونها	عفوًا<br>نادرا ما<br>بطريقه ما<br>معظمها<br>بقدر ما تحب	

MAKERERE UNIVERSITY
SCHOOL OF HEALTH SCIENCES
APPROVED
VALID [illegible]
01 MAY 2020
RESEARCH & ETHICS COMMITTEE
P. O. BOX 7072, KAMPALA

1108	وينبغي ان تكون الأمهات قادرات على الحصول على زيارات من الناس التحقق منها ومعرفه ما إذا كانت على ما يرام	عفوًا<br><br>نادرا ما<br><br>بطريقه ما<br><br>معظمها<br><br>بقدر ما تحب	
1109	يجب على الأمهات الحصول على فرص للتحدث مع شخص ما حول المشاكل في العمل أو مع أسرتهم	عفوًا<br><br>نادرا ما<br><br>بطريقه ما<br><br>معظمها<br><br>بقدر ما تحب	
1110	يجب على الأمهات الحصول على فرص للتحدث مع شخص يثقون به حول مشاكلهم الشخصية	عفوًا<br><br>نادرا ما<br><br>بطريقه ما<br><br>معظمها<br><br>بقدر ما تحب	
1112	يجب على الاباء التحدث عن زوجاتهم حول المسائل المالية	عفوًا<br><br>نادرا ما<br><br>بطريقه ما<br><br>معظمها<br><br>بقدر ما تحب	
1113	يجب ان تكون الأمهات قادرات على الحصول على دعوات للخروج والقيام بأشياء مع أشخاص آخرين	عفوًا<br><br>نادرا ما<br><br>بطريقه ما<br><br>معظمها<br><br>بقدر ما تحب	
1114	يجب ان تكون الأمهات قادرات على	عفوًا	

MAKERERE UNIVERSITY
SCHOOL OF HEALTH SCIENCES
APPROVED
VALID UNTIL
01 MAY 2020
RESEARCH & ETHICS COMMITTEE
P. O. BOX 7072, KAMPALA

	الحصول على نصائح مفيده حول الأشياء الهامة في الحياة	نادرا ما<br>بطريقه ما<br>معظمها<br>بقدر ما تحب	
1115	يجب على الاباء دعم زوجاتهم مع النقل عندما يحتاجون اليها	عفوًا<br>نادرا ما<br>بطريقه ما<br>معظمها<br>بقدر ما تحب	
1116	يجب على الاباء ان يدعموا أزواجهم عندما يمرضون في السرير	عفوًا<br>نادرا ما<br>بطريقه ما<br>معظمها<br>بقدر ما تحب	
1117	الأمهات بحاجه إلى بعضالجسم لتتحول إلى للحصول على اقتراحات حول كيفية التعامل مع الاسرهالمشكله؟	عفوًا<br>نادرا ما<br>بطريقه ما<br>معظمها<br>بقدر ما تحب	
1118	وينبغي ان الاباء حelp مع الاعمال اليومية عندما الزوج هو المرضي؟	عفوًا<br>نادرا ما<br>بطريقه ما<br>معظمها<br>بقدر ما تحب	
1119	يجب ان يكون لدي الأمهات شخص ما من هم يمكن ان يكون وقتا طيبا مع؟	عفوًا	

MAKERERE UNIVERSITY
SCHOOL OF HEALTH SCIENCES
APPROVED
VALID [illegible]
01 MAY 2020
RESEARCH & ETHICS COMMITTEE
P. O. BOX 7072, KAMPALA

HHID__________ FATHERID____________

		نادرا ما<br>بطريقه ما<br>معظمها<br>بقدر ما تحب	

MAKERERE UNIVERSITY
SCHOOL OF HEALTH SCIENCES
APPROVED
VALID [illegible]
01 MAY 2020
RESEARCH & ETHICS COMMITTEE
P. O. BOX 7072, KAMPALA

HAND WASHING PROMOTES GOOD HEALTH

Our hands constantly get in touch with dirt and germs as we work or interact with other people. You and your family members need to wash hands frequently to keep away germs and prevent illnesses such as diarrhea.

When to wash hands

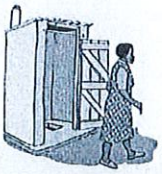

After visiting the toilet or pit latrine.

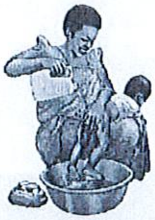

After cleaning baby's faeces or changing baby's diaper/ napkin

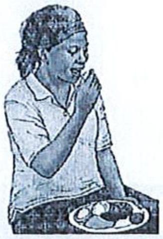

Before preparing food, before eating and after eating

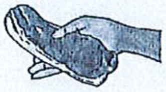

After handling blood, raw meat or uncooked eggs

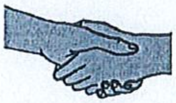

After shaking hands

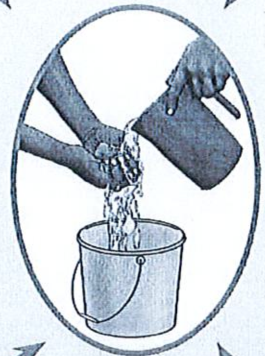

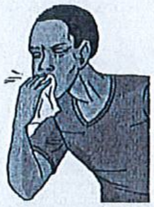

After sneezing, coughing or blowing your nose

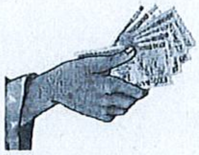

After touching/ counting money. Think about how many people touch money.

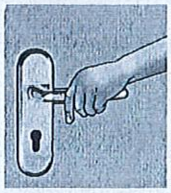

After handling surfaces that many people touch such as doorknobs

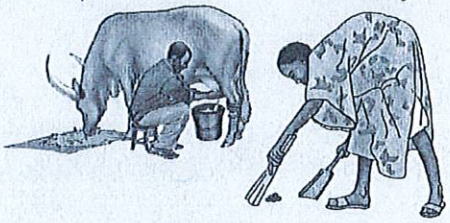

After touching/ cleaning animals, animal waste or garbage.

MAKERERE UNIVERSITY
SCHOOL OF HEALTH SCIENCES
APPROVED
VALID UNTIL
01 MAY 2020
RESEARCH & ETHICS COMMITTEE
P. O. BOX 7072, KAMPALA

What you need for hand washing:

1. Soap or ash.
2. Clean running water facility such as tippy tap or water tap placed where everyone can reach . It is better for each home to have more than one hand washing facility : one near the latrine and another near the kitchen.

How to wash hands

To wash your hands clean, use the steps below:

- Wet your hands with water.

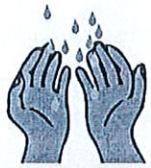

- Put soap or ash on the hands.

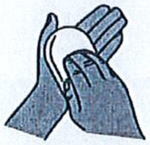

- Rub the palms, back of hands, between fingers, under finger nails and wrists repeatedly. Give special attention to the area between fingers and under the nails. This is where most germs hide.

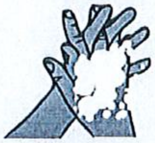

- Rinse hands until all the soap or ash is off.

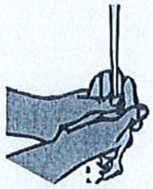

- Dry your hands by shaking them dry. Do not use public towels to dry your hands.

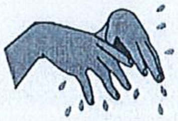

Teach all your family members including children about how and when to wash hands to protect them from diseases such as diarrhea.

USAID FROM THE AMERICAN PEOPLE

Community Connector
The Integrated Nutrition and Agriculture Project

REPUBLIC OF UGANDA

unicef

MAKERERE UNIVERSITY
SCHOOL OF HEALTH SCIENCES
APPROVED
VALID UNTIL
01 MAY 2020
RESEARCH & ETHICS COMMITTEE
P. O. BOX 7072, KAMPALA

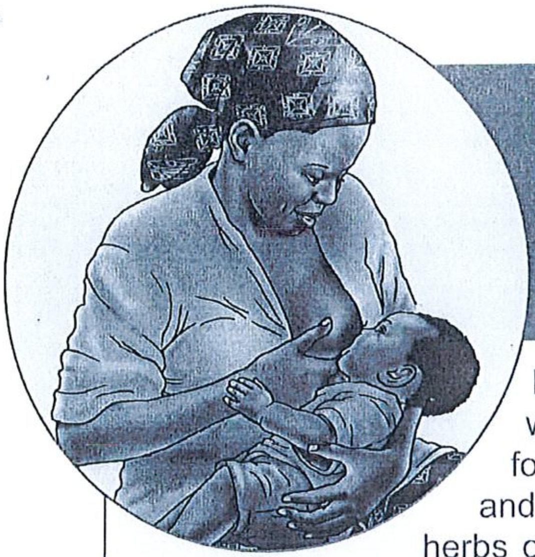

Did you know that babies between 0 - 6 months need to be fed on ONLY BREAST MILK?

Breast milk is the best food for babies who are 0 - 6 months old. It has all the food values a baby needs to grow well and healthy. Water, glucose, gripe water, herbs or tinned milk can make the baby get diseases like diarrhea which can lead death.

Benefits of giving only breast milk:

1. Breast milk has all food values for the baby.
2. It is free.
3. It is clean.
4. It is always there for the baby.
5. It has food and water to satisfy the baby for the first six months.
6. It takes away thirst.
7. It protects baby from diseases.
8. It makes mother and baby bond and strengthens their relationship.

Note: Mothers need to breastfeed the babies even when they think their breast milk is little breast milk or it is not there at all. The more a mother breastfeeds, the more breast milk is made.

When to start breastfeeding?

Mothers need to start breastfeeding the baby within the first hour after birth. Continue to breastfeed until the baby is six months old. Baby should be breast-fed day and night for as many times as the baby needs.

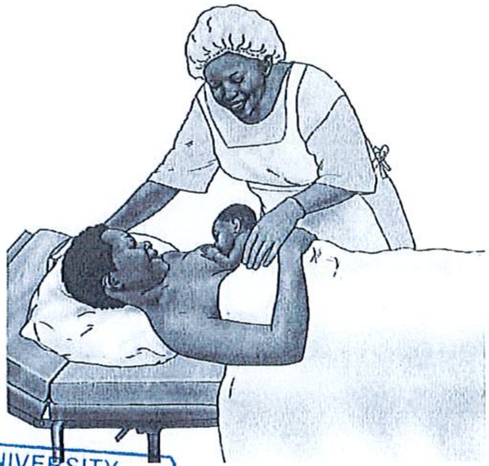

MAKERERE UNIVERSITY
SCHOOL OF HEALTH SCIENCES
APPROVED
VALID UNTIL
01 MAY 2020
RESEARCH & ETHICS COMMITTEE
P. O. BOX 7072, KAMPALA

How to breast feed

Both mother and baby need to be relaxed to breastfeed well.

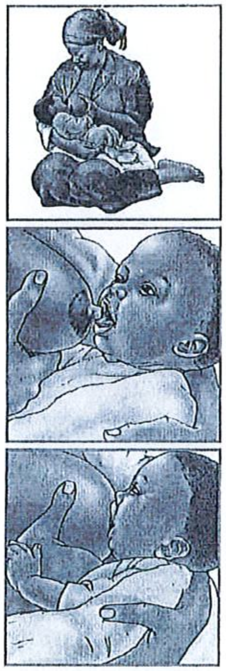

1. The mother should sit in a comfortable upright position and place the baby on her laps.

2. She should hold the baby close to her and using her hand and thumb, direct the breast towards the baby's mouth.

3. Press the dark part of the breast to prevent the breast from blocking baby's nose.

Signs that baby is well attached to breast:

1. Mouth is wide open.
2. Chin is touching the breast.
3. More of the dark part of the breast is showing above than below the nipple.
4. Baby's lower lip is turned out.

MAKERERE UNIVERSITY
SCHOOL OF HEALTH SCIENCES
APPROVED
VALID UNTIL
01 MAY 2020
RESEARCH & ETHICS COMMITTEE
P. O. BOX 7072, KAMPALA

Signs that baby is getting enough milk:

1. Baby urinates many times during the day and at night.
2. Urine has water-like colour.
3. Urine does not have strong smell.
4. Baby is growing bigger and looking healthy.

NOTE: Breast-feeding mothers should eat more meals and snacks in order to produce enough breast milk.

Child Nutrition

Give your baby ONLY breastmilk for 6 months; Do not give anything else, NOT even water. The breastmilk is all the baby needs and contains all nutrients.

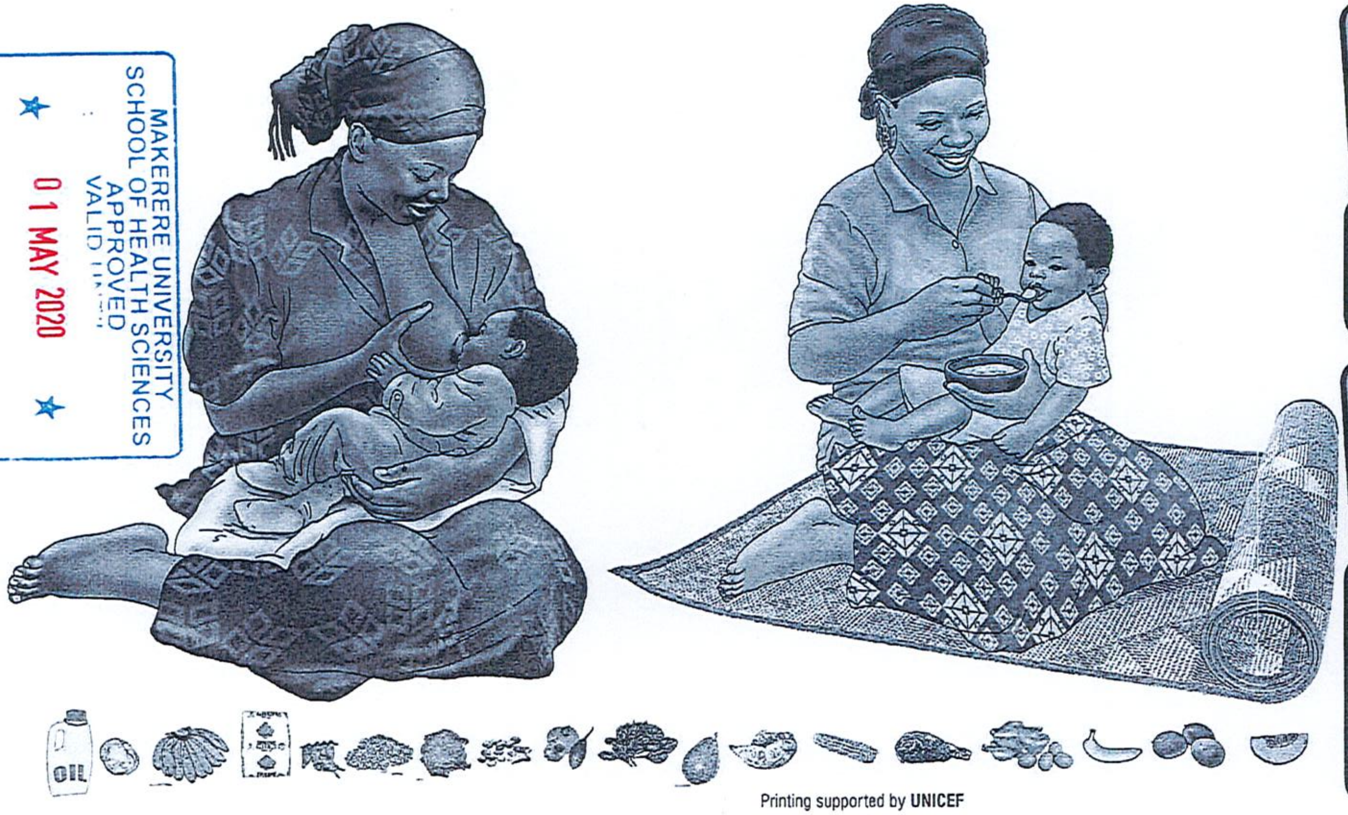

MAKERERE UNIVERSITY
SCHOOL OF HEALTH SCIENCES
APPROVED
VALID UNTIL
01 MAY 2020
RESEARCH & ETHICS COMMITTEE
P. O. BOX 7072, KAMPALA

IMPORTANT TO NOTE:

- Exclusively breastfeed your baby for 6 months; Breast milk is the best start for your baby
- Do not give anything else, not even water or other liquids as this reduces breast milk production and intake leading to poor growth.
- Breast feed the baby often, at least ten times day and night.
- Starting at six months your baby needs other foods in addition to breast milk. Feed your baby 2 to 3 times a day.
- Continue breast feeding your baby on demand, day and night till 2 years as breast milk continues to be the most important part of your baby's diet.

Printing supported by UNICEF

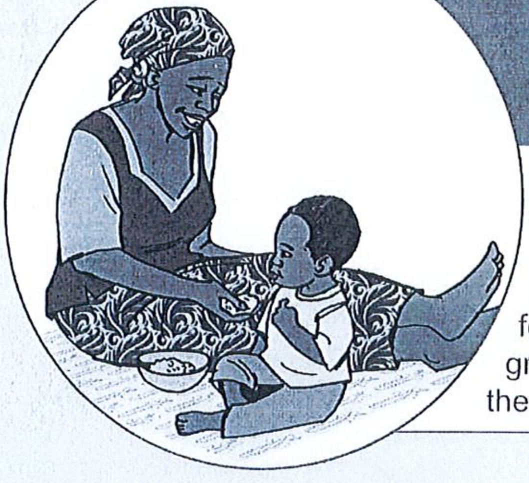

FOOD FOR 6 TO 24 MONTHS OLD CHILDREN

At six months, parents and other care givers like you need to start giving the baby other nutritious drinks and foods. But mothers must continue breast feeding the baby. This helps the child to grow well and start standing and walking at the right age.

How to feed a 6 -24 months old child:

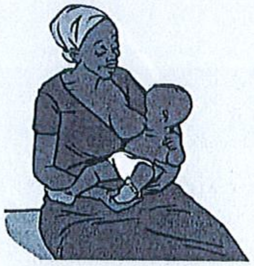

- Between six and nine months the baby should be breastfed first and then given other nutritious soft foods.
- The child should be started on soft foods and fruits.
- The child should be given at least three small main meals and three snacks in between meals daily.
- Ensure the baby has enough food for his or her small size.
- Meal should be made from a mixture of different foods mashed together.
- Feed the child on different foods at each meal every day.
- Prepare porridge from maize, millet and sorghum flour.

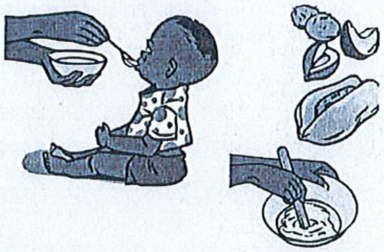

Food nutrients need to be improved by adding:

- Little sugar and oil/ blue band margarine/ butter in porridge or mashed foods.
- A spoonful of groundnut paste in baby porridge and mashed food.
- A spoonful of mashed avocado into the baby food.
- A spoonful of mukene flour.
- A spoonful of mashed beans, milled beans, chickpeas, milled pumpkin, or greens.
- Give molted porridge from fermented cereals (ferment or germinate millet, sorghum or maize seeds before milling into flour).

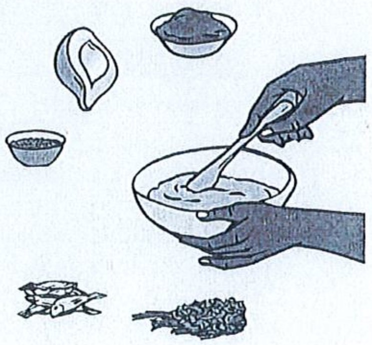

MAKERERE UNIVERSITY
SCHOOL OF HEALTH SCIENCES
APPROVED
VALID UNTIL
01 MAY 2020
RESEARCH & ETHICS COMMITTEE
P. O. BOX 7072, KAMPALA

Food preparation

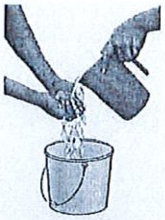

- Anyone preparing the food must keep good hygiene while preparing food for young children. It helps to prevent diseases like worms and diarrhea.
- Wash hands before preparing the food
- Cover the food when cooking to avoid making the food unclean.
- Serve the food on a clean plate and drinks in clean cups.
- Wash hands before serving the food.
- Wash hands before giving the baby food.
- Keep the food in a clean and covered container.

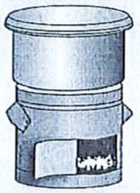

How to prepare a meal from beans, meat and matooke mixture.

Ingredients:

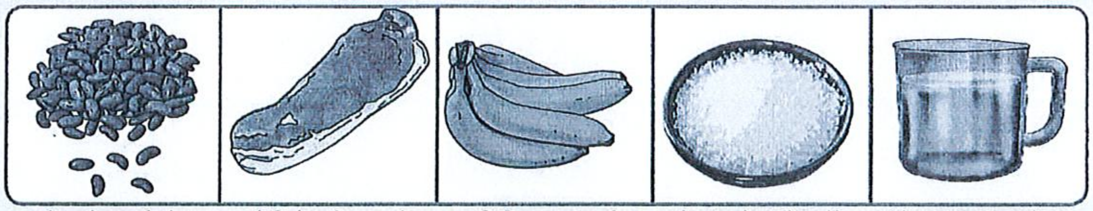

1 palm of dry beans or peas(90 g)	1 fist of meat (60 g)	3 fingers of matooke (300-500 g)	1 pinch of salt	½mug of water (250 ml)

METHOD

1. Measure the dry beans and soak overnight or for about 6 hours. Remove the skins and wash them. The skinned beans having expanded, now becomes 2 palmful.
2. Scrape the meat, mix it with clean water which had been boiled and cooled,in a container and dry to separate the particles of the meat.
3. Peel the matooke, cut them into small pieces and wash them.
4. Mix the skinned beans, scrapped meat, pieces of matoke, water and salt into a clean saucepan. Cover and steam for 3 hours. When the food is ready, mash it.
5. Feed the child when food is still hot.

Note: Irish potatoes, sweet potatoes, cassava, yams can be prepared the same way as matooke. Dry peas can be prepared the same way as dry beans.

USAID
FROM THE AMERICAN PEOPLE
Community Connector
The Integrated Nutrition and Agriculture Project
REPUBLIC OF UGANDA
unicef

MAKERERE UNIVERSITY
SCHOOL OF HEALTH SCIENCES
APPROVED
VALID UNTIL
01 MAY 2020
RESEARCH & ETHICS COMMITTEE
P. O. BOX 7072, KAMPALA
