# Supplementary material for: Care groups in an integrated nutrition education intervention improved infant growth among South Sudanese refugees in Uganda’s West Nile post-emergency settlements: A cluster randomized trial
Source: PLoS One. 2024 Mar 15;19(3):e0300334. doi: 10.1371/journal.pone.0300334 (PMC10942045; doi:10.1371/journal.pone.0300334)
Supplement: S2 File — (DOCX) [file pone.0300334.s006.docx]

Supplementary materials

| S1a Table: Tests of within-subjects effects on infant length-for-age z-scores (LAZ)^a^ | | | | | | |
| --- | --- | --- | --- | --- | --- | --- |
| **Variable**^⸷^ | Type III SS | df | Mean square | F | sig. |  |
| Study arm*social support score | 139.27 | 6.00 | 23.21 | 28.91 | <0.001 |  |
| Study arm | 76.02 | 4.00 | 19.00 | 24.75 | <0.001 |  |
| Social support score | 19.16 | 2.00 | 9.58 | 12.12 | <0.001 |  |
| Infant age | 0.37 | 2.00 | 0.19 | 0.23 | 0.791 |  |
| Supports mother the most | 5.81 | 2.00 | 2.91 | 3.67 | 0.026 |  |
| Number of living children | 1.17 | 2.00 | 0.59 | 0.74 | 0.477 |  |
| Mother’s height | 2.36 | 2.00 | 1.18 | 1.49 | 0.226 |  |
| Socioeconomic status | 0.84 | 2.00 | 0.42 | 0.53 | 0.590 |  |
| Religion | 0.45 | 2.00 | 0.23 | 0.29 | 0.750 |  |
| Child sex | 4.74 | 2.00 | 2.37 | 2.99 | 0.051 |  |
| Childbirth weight | 2.92 | 2.00 | 1.46 | 1.85 | 0.159 |  |
| Ethnicity | 10.96 | 2.00 | 5.48 | 7.25 | 0.010 |  |
| Error (Time) | 443.57 | 560.00 | 0.79 |  |  |  |
| ^a^ Sphericity assumed; ^⸷^ within-subjects effects assessed with time effect; SS sum of squares; df degrees of freedom; ^*^Interaction | | | | | | |

| S1b Table: Tests of within-subjects effects on infant weight-for-age z-scores (WAZ)^†^ | | | | | |
| --- | --- | --- | --- | --- | --- |
| **Variable**^⸷^ | Type III SS | df | Mean square | F | sig. |
| Study arm*social support score | 36.73 | 5.76 | 6.38 | 12.70 | <0.001 |
| Study arm | 20.63 | 3.85 | 5.16 | 10.32 | <0.001 |
| Social support score | 2.00 | 1.88 | 1.06 | 2.12 | 0.160 |
| Infant age | 0.71 | 1.93 | 0.37 | 0.73 | 0.476 |
| Supports mother the most | 0.72 | 1.93 | 0.38 | 0.75 | 0.469 |
| Number of living children | 0.85 | 1.93 | 0.44 | 0.88 | 0.411 |
| Mother’s height | 0.15 | 1.93 | 0.08 | 0.15 | 0.852 |
| Socioeconomic status | 0.15 | 1.93 | 0.08 | 0.16 | 0.846 |
| Religion | 0.17 | 1.93 | 0.09 | 0.17 | 0.834 |
| Child sex | 1.82 | 1.93 | 0.95 | 1.88 | 0.155 |
| Childbirth weight | 2.67 | 1.93 | 1.39 | 2.76 | 0.066 |
| Ethnicity | 2.96 | 1.93 | 1.55 | 0.05 | 0.011 |
| Error (Time) | 270.70 | 539.38 | 0.50 |  |  |
| ^†^ Greenhouse-Geisser correction; ^⸷^ within-subjects effects assessed with time effect; SS sum of squares; df degrees of freedom; ^*^Interaction | | | | | |

| S1c Table: Tests of within-subjects effects on infant weight-for-length z-scores (WLZ)^†^ | | | | | |
| --- | --- | --- | --- | --- | --- |
| **Variable**^⸷^ | Type III SS | df | Mean square | F | sig. |
| Study arm*social support score | 18.24 | 5.28 | 3.46 | 3.38 | 0.004 |
| Study arm | 11.86 | 3.52 | 3.38 | 3.24 | 0.016 |
| Social support score | 3.06 | 1.75 | 1.74 | 1.67 | 0.193 |
| Infant age | 0.39 | 1.76 | 0.22 | 0.21 | 0.780 |
| Supports mother the most | 3.34 | 1.76 | 1.90 | 1.84 | 0.165 |
| Number of living children | 0.48 | 1.76 | 0.27 | 0.26 | 0.741 |
| Mother’s height | 0.02 | 1.76 | 0.01 | 0.01 | 0.984 |
| Socioeconomic status | 0.45 | 1.76 | 0.26 | 0.25 | 0.752 |
| Religion | 1.39 | 1.76 | 0.79 | 0.76 | 0.451 |
| Child sex | 0.24 | 1.76 | 0.14 | 0.13 | 0.849 |
| Childbirth weight | 0.85 | 1.82 | 0.47 | 0.46 | 0.610 |
| Ethnicity | 0.28 | 1.77 | 0.16 | 0.15 | 0.838 |
| Error (Time) | 509.25 | 492.47 | 1.03 |  |  |
| ^†^ Greenhouse-Geisser correction; ^⸷^ within-subjects effects assessed with time effect; SS sum of squares; df degrees of freedom; ^*^Interaction | | | | | |

| S2a Table: Association among study arms and maternal social support in the Adjumani post-emergency settlements | | | | |
| --- | --- | --- | --- | --- |
| **Groups** | **df** | **MS** | **F** | ***p*-value** |
| Between groups | 2 | 25076.53 | 373.17 | <0.001 |
| Within groups | 1170 | 67.2 |  |  |
| Total | 1172 |  |  |  |

| S2b Table: Pairwise comparisons of study arm and maternal social support in Adjumani post-emergency settlements | | | |  |  |
| --- | --- | --- | --- | --- | --- |
| **(I) Group** | **(J) Group** | ***MD* (I-J)** | **Std. Error** | **95% CI** | |
|  |  |  |  | **Lower Bound** | **Upper Bound** |
| Control | Mothers-only | -8.8*** | 1.2 | -11.7 | -5.9 |
|  | Parents-combined | -17.1*** | 1.3 | -20.0 | -14.1 |
| Mothers-only | Control | 8.8*** | 1.2 | 5.9 | 11.7 |
|  | Parents-combined | -8.3*** | 1.1 | -10.9 | -5.7 |
| Parents-combined | Control | 17.1*** | 1.3 | 14.1 | 20.0 |
|  | Mothers-only | 8.3*** | 1.1 | 5.7 | 10.9 |
| *MD*-mean difference; Tukey Kramer correction applied for unequal sample sizes; ^*^*p* < .05, ^**^*p* < .01, ^***^*p* < .001 | | | | | |

| S3 Table: Determination of strength of association and effects size | | | | |
| --- | --- | --- | --- | --- |
| Measure Formula | Measure description | Study arm*social support for infant LAZ | Study arm*social support for infant WAZ | Study arm*social support for infant WLZ |
| $\hat{\omega}_{Y\vert A\cdot B}^{2}=\frac{\frac{p-1}{np}\left( MS\mathrm{Treat}-MSE \right)}{\frac{p-1}{np}\left( MS\mathrm{Treat}-MSE \right)+MSE}$ | Measure of association | 0.850 | 0.723 | 0.341 |
| $\hat{f}_{*}=\sqrt{\frac{\hat{\omega}_{Y\vert*}^{2}}{1-\hat{\omega}_{Y\vert*}^{2}}}$ | Effects size | 2.38 | 1.62 | 0.719 |
| $\hat{\omega}_{Y\vert A\cdot B}^{2}$ strength of association; $\hat{f}_{*}$ effects size; p, study arms; n, number of levels(time); MSTreat mean square treatment(interaction); MSE mean square error ^*^Interaction | | | | |

| S4 Table: Questions used to measure social support | |
| --- | --- |
|  | **Emotional/informational support^‡^** |
|  | Someone you can count on to listen to you when you need to talk |
|  | Someone to give you information to help you understand a situation |
|  | Someone to give you good advice about a crisis |
|  | Someone to confide in or talk to about yourself or your problems |
|  | Someone whose advice you really want/need |
|  | Someone to share your most private worries/concerns and fears with |
|  | Someone to turn to for suggestions about how to deal with a personal problem |
|  | Someone who understands your problems |
|  | **Tangible support^‡^** |
|  | Someone to help you if you were confined to bed when sick |
|  | Someone to take you to the health facility if you needed it |
|  | Someone to prepare your meals if you were unable to do it yourself |
|  | Someone to help with daily chores if you were sick |
|  | **Affectionate support** |
|  | Someone who shows you affection and care |
|  | Someone to make you feel respected |
|  | Someone who hugs you/ embraces you |
|  | **Positive social interaction** |
|  | Someone to have a good time with |
|  | Someone to get together with for relaxation |
|  | Someone to do something enjoyable with |
|  | **Additional item^‡^** |
|  | Someone to do things with to help you get your mind off worries |
| **^‡^**Emphasis of the questions by the enumerator to the mother was made towards childcare and feeding, as well as mother’s health and nutrition in relation to the child’s wellbeing [47]. | |

S5 Table: Questions and key messages used in the intervention training modules

| Module 1:  Group Dynamics | A group are people who interact with one another and think of themselves as belonging together  *Key Messages*   - The members of the group have a strong sense of belonging to the group - The more the group members work and learn together, the greater the influence it would have on the members - Groups are the biggest part of life - Most work is done in and with groups - Knowing how and why groups function or fail to function is a key to success |
| --- | --- |
| Peer support groups | Ten to twenty people meet and interact every two weeks on issues of child and household health, and nutrition. These people have a sense of belonging or oneness  *What we need to know about this Care Group*  Its where we interact with other members on health and nutrition issues with expectations about other’s behavior  *Group Guidelines*   - It allows the expression of different views. - Participants listen to understand and gain insight. - Questions are asked from a position of curiosity. - Participants speak with free minds. - New information surfaces - Respect each other’s views - Give each one a chance to communicate; listen - No judging - No criticizing   *Know your group leader:*  If the selection is done, then introduce the leader to the other members. (this position is voluntary)  We need to elect/ select a group leader.  *Questions*   1. What do you think of the images you see on the charts in the context of our topic? 2. Why are groups important in the community? 3. What are some of the challenges groups face in the community? 4. How can we overcome these challenges? |
| Module 2:  Antenatal Care | *Prompting questions*   1. How does going for antenatal care help the mom and baby? 2. What are some reasons mothers do not go for antenatal care?   *Key messages*   - Mothers should go to the health center as soon as they know they are pregnant. - Mothers should go to the health center at least eight times while pregnant for antenatal care. |
| Early initiation of breastfeeding | *Prompting questions*   1. How soon after birth should mothers breastfeed their baby? 2. Why is breastfeeding right after birth important?   *Key messages*   - Before mothers deliver, they should tell the health care team that they want to breastfeed immediately after birth. - The mother should breastfeed the baby within one hour of giving birth. |
| Module 3:  Postnatal care | *Prompting question*   1. How often should parents bring their babies to the health center after birth? 2. Why is it important to take your baby to the health center after giving birth?   *Key messages*   - Go to the health center with your baby at one week, then at two weeks, and the six weeks after you give birth. - Take your baby immediately to a trained health worker or clinic if he/she refuses to eat and is very weak, if he/she is vomiting, or if he/she has diarrhea. |
| WASH (Mom and household) | *Prompting questions*   1. How do we know the water is safe to drink?   (Prompt to the need of protecting water because we can’t see germs)   1. Why do we need to keep latrines clean and covered?   *Key messages*   - Keep your drinking water in a clean and covered container, and place it in a clean and dry space - Keep your latrines clean, with no feces around, and covered at all times |
| Module 4:  Exclusive breastfeeding | *Prompting questions*   1. For how long should a baby be fed ONLY breastmilk? 2. Does breast milk provide all the baby needs for the first six months of life?   *Key messages*   - For the first six months, a baby should only receive breastmilk. - A baby should not have any other foods or liquids, even water before six months old. |
| Father involvement in breastfeeding | *Prompting questions*   1. What are ways that fathers can help breastfeeding mothers? 2. Do fathers in your community support breastfeeding mothers?   *Key messages*   - Fathers can support breastfeeding mothers by helping them with household duties. - Fathers can support breastfeeding mothers by encouraging and praising mothers for breastfeeding their babies. |
| Module 5:  Complimentary Feeding practices | *Prompting Questions*   1. When should children start being given soft foods? 2. What are some of the foods that we can start giving children?   *Key message*   - When a child is 6 months, start giving soft foods. - Give a balance of foods at 6 months, and continue breastfeeding for up to 2 years |
| Social Support | *Questions*   1. Why is it important for us to meet to discuss health and nutrition? 2. What can we do to ensure that we learn from one another?   *Key message*   - Care Group is about working together and supporting one another (being there for one another) - Together we can do better than when we are alone |
| Module 6:  Good Nutrition | *Prompting Questions*   1. Why is good nutrition important for growth? 2. What can we do to ensure we get good nutrition?   *Key messages*   - Good nutrition helps the growth, development, and healing of the body from sickness - Good nutrition helps makes energy in the body for movement and working |
| Social support and Kitchen Gardening | *Prompting Questions*   1. What are the kitchen gardens?   (Small gardens are set up on small pieces of land or soil in containers around the house)   1. What are the biggest challenges for families in creating kitchen gardens?   Probe the groups for answers (Lack of commitment, lack of seed, drought, lack of gardening tools ...)  *Key Messages*   - Through our Care Groups, we can be able to support one another in backyard farming. - Grow some vegetables around the home to improve the value of the food prepared for children. |
| Module 7:  Child development | *Prompting questions*   1. Why is a baby's development important? 2. What can babies learn from their parents?   *Key messages*   - A Baby's development is as important as a baby's health - Parents are the baby's first teachers |
| Child stimulation | *Prompting questions*   1. Why is talking and singing to babies important? 2. What do you do when your baby cries?   *Key messages*   - Talk and sing to your baby as often as you can - Comfort your baby when he or she cries |
| Module 8:  Exclusive breastfeeding | *Prompting questions*   1. In your community, what foods are good for breastfeeding mothers to eat? 2. How does a mother's eating affect her breast milk?   *Key messages*   - Breastfeeding mothers need to eat more food and a variety of food. - Healthy eating helps mothers produce enough breast milk for their babies. |
| WASH | *Prompting questions*   1. When do we need to wash our hands and how?   (Prompt to the need to wash hands even if they don’t look dirty, germs are invisible)   1. How do you keep your house and compound clean?   *Key messages*   - Wash your hands with clean water and soap (or ash) before preparing food, when cooking, before eating, before breastfeeding, after cleaning your child's bottom, and after defecating. - Clean your house and your compound at least once a day. |
| Module 9:  Feeding sick child | *Prompting Questions*   1. What can we feed a child when it is sick? 2. What are some of the signs we need to look out for to know that a child is sick?   *Key messages*   - Sick children under 6 months should continue to breastfeed more frequently - Take the child to the health center, and get advice from the health worker |
| Father involvement in childcare | *Prompting questions*   1. Why is it important that fathers help in taking care of the baby? 2. What can a father do to help the mother raise their baby?   *Key messages*   - Fathers and mothers are both responsible for raising their babies. - Fathers and mothers should agree on sharing household chores. |
| Module 10:  Father involvement in child stimulation | *Prompting questions*   1. What do fathers do when they are alone with their baby? 2. How often do fathers hold the baby?   *Key messages*   - Fathers need time alone (one-on-one) to talk and play with the baby. - Fathers should hold the baby at least once a day. |
| Household nutrition diversity | *Prompting Questions*   1. What are some of the challenges faced in the community toward good household nutrition? 2. How can we overcome the challenges of poor feeding in a home?   *Key Messages*   - Healthy food keeps a child and family members healthy, strong, and smart. - Eating more food in different colors means more nutrition helps protect a child and family members from sickness. |
| Module 11:  Child stimulation | *Prompting questions*   1. What do you do when you are alone with your baby? 2. What do you do when you see your baby move around and grab things?   *Key messages*   - Take time every day to play with your baby - Encourage your baby to move around on his own |
| Peer support | *Prompting Questions*   1. How can group meetings help fathers improve support for their families? 2. How can the community support fathers in taking care and providing for their family?   *Key Messages*   - In social groups, people get to exchange ideas and share experiences on best health practices - In social groups, people offer and receive support to help improve the health, and well-being, of their families. |
| Module 12:  Child WASH | *Prompting questions*   1. How do you keep the areas where your child plays clean and dry? 2. Why is it important to put your child’s feces in the latrine?   *Key messages*   - Always keep all areas where your child plays clean and dry - Always throw away your child's feces in the latrine |
| Continued breastfeeding | *Prompting questions*   1. In your community, for how long do most mothers breastfeed their baby? 2. What are some reasons that mothers stop breastfeeding?   *Key messages*   - Breastfeeding should continue until the child is at least two years old. - Breastmilk helps protect the child from becoming sick. |
| Module 13:  Feeding a Sick Child | *Prompting questions*   1. What foods should you give a sick child with dehydration? 2. Where can parents get support in case a child falls sick?   *Key messages*   - Take a child with danger signs (vomiting, diarrhea, fever, lack of appetite) to the Health Centre immediately. - Give any child (above 6 months) with dehydration or diarrhea oral rehydration salt (ORS) |
| Responsive Feeding | *Prompting questions*   1. Do you think babies can know when they are full or hungry? 2. How do you know when your baby likes or dislikes a food?   *Key messages*   - Make feeding times happy for the baby by being patient and making eye contact. - Give new foods many times, they may not like new foods in the first few tries but keep trying. |
| Module 14:  Child Feeding – Introduction of solid and semi-solid foods | *Prompting questions*   1. What foods do we give infants when they reach 6 months? 2. Why should mothers still breastfeed their babies even if they start to eat after 6 months?   *Key messages*   - Start giving your child foods like porridge, mashed banana, or mashed potato when he/she reaches 6 months. - Breastfeed first before giving other foods. |
| Complementary feeding (Father involvement) | *Prompting questions*   1. How can fathers help the mothers feed the baby? 2. What prevents fathers from helping the mothers feed the baby?   *Key messages*   - Fathers should help mothers find good food for their baby. - Fathers can help the mother feed the baby. |
| Module 15:  Complementary feeding | *Prompting questions*   1. What kind of foods should a baby older than 6 months eat? 2. Why is eating a colorful or a variety of food important for babies older than 6 months?   *Key messages*   - Provide a variety of colorful food for a child older than six months. - Give meat, chicken, fish, or eggs at least 3 times a week or every day if you can. |
| Complementary feeding (Cooking Demonstration-session) | Sample meals from local blended staples that may be used for the introduction of solid semi-solid and soft foods |
| Module 16:  Water, Sanitation, and Hygiene | *Prompting questions*   1. What needs to be cleaned before you feed your child? 2. What happens if you give your child leftover foods that were not covered?   *Key messages*   - Use clean water and soap (or ash) to clean your hands, your child's hands, and the utensils you use before feeding your child - Cover your food and heat leftovers before feeding to your child |
| Complementary feeding (Cooking Demonstration-session) | Sample meals from local blended staples that may be used for the introduction of solid semi-solid and soft foods |
| Module 17:  Complementary Feeding | *Prompting questions*   1. How many times in a day should children 6-8 months eat complementary food? 2. What prevents parents from feeding their children 2-3 times a day?   *Key messages*   - Breastfed babies 6-8 months old need to be fed food at least two times a day. - Babies 6-8 months old who are not breastfed need to be fed foods at least four times a day. |
| Complementary feeding (Cooking Demonstration-session) | Sample meals from local blended staples that may be used for the introduction of solid semi-solid and soft foods |
| Module 18:  Maternal mental health | *Prompting questions*   1. What do people in your community do when they feel sad or angry? 2. How are people who feel stressed supported by the community?   *Key messages*   - When people feel sad or angry, it’s important to talk to someone or get help.   Psychosocial resources in the community are there to help all people. |
| Complementary feeding (Cooking Demonstration-session) | Sample meals from local blended staples that may be used for the introduction of solid semi-solid and soft foods |
| Module 19:  Maternal mental health | *Prompting questions*   1. How does the community view people who have mental health issues? 2. What are some of the causes of mental health issues in your community?   *Key messages*   - Mental health is a health condition and is not the individual’s fault.   It is healthy and important for people to seek out help for mental health issues. |
| Complementary feeding (Cooking Demonstration-session) | Sample meals from local blended staples that may be used for the introduction of solid semi-solid and soft foods |
| Module 20:  Refresher training: Complementary feeding | *Prompting questions*   1. What kind of foods should a baby older than 6 months eat? 2. Why is eating a colorful or a variety of food important for babies older than 6 months?   *Key messages*   - Provide a variety of colorful food for a child older than six months. - Give meat, chicken, fish, or eggs at least 3 times a week or every day if you can. |
| Complementary feeding (Cooking Demonstration-session) | Sample meals from local blended staples that may be used for the introduction of solid semi-solid and soft foods |
| The pictures used in the training modules were adopted from UNICEF IYCF materials (35, 42-44). | |

S1 Fig: Study Gantt Chart
